# Live‐Cell RNA Imaging via Clickable Tri*PPP*ro Nucleotide Reporters

**DOI:** 10.1002/anie.202516613

**Published:** 2025-11-27

**Authors:** J. Iven H. Knaack, Eileen List, Dörte Stalling, Vincente T. Sterrenberg, Chris Meier, Hans‐Achim Wagenknecht, Jens B. Bosse

**Affiliations:** ^1^ Organic Chemistry Department of Chemistry Faculty of Sciences University of Hamburg Martin‐Luther‐King‐Platz 6 20146 Hamburg Germany; ^2^ Centre for Structural Systems Biology (CSSB) Notkestraße 85, Building 15 22607 Hamburg Germany; ^3^ Institute of Organic Chemistry Karlsruher Institute of Technology (KIT) Fritz‐Haber‐Weg 6 76131 Karlsruhe Germany; ^4^ Institute of Virology Hannover Medical School (MHH) 30625 Hannover Germany; ^5^ Leibniz Institute of Virology (LIV) 20251 Hamburg Germany; ^6^ Cluster of Excellence RESIST (EXC 2155) Hannover Medical School 30625 Hannover Germany

**Keywords:** Click‐chemistry, Live‐cell Imaging, Nucleotides, RNA Labeling, Tri*PPP*ro‐compounds

## Abstract

Understanding RNA synthesis and dynamics in cells requires efficient labeling strategies that are not only compatible with cellular environments but can be performed in living cells. We developed a robust, bio‐orthogonal approach for live‐cell RNA labeling using Tri*PPP*ro (triphosphate prodrug) chemistry. This strategy enables the intracellular delivery of sterically demanding nucleoside triphosphates modified with inverse electron‐demand Diels–Alder (IEDDA)‐reactive groups, specifically *trans*‐cyclooctene (2TCO*a*) and bicyclo[6.1.0]nonyne (BCN). Once hydrolyzed inside cells, these Tri*PPP*ro‐modified uridines and cytidines are metabolically incorporated into nascent RNA by endogenous RNA polymerases. Subsequent IEDDA reaction with a dual‐fluorogenic tetrazine‐cyanine styryl dye conjugate allows wash‐free, high‐contrast imaging of RNA synthesis in cells. We demonstrate efficient RNA labeling, including nucleolar localization and specificity for newly transcribed RNA, validated by transcriptional inhibition and colocalization with ribosomal RNA. Comparative analyses confirm that Tri*PPP*ro delivery surpasses conventional transporter‐based systems in both labeling efficiency and cellular compatibility. This platform offers a modular, non‐genetic, and highly specific method for real‐time RNA imaging, with broad applicability for RNA biology and antiviral research.

## Graphical abstract

The intracellular delivery of *trans*‐cyclooctene and bicyclo[6.1.0]nonyne as sterically demanding groups for the inverse electron‐demand Diels–Alder was achieved by the triphosphate prodrug (Tri*PPP*ro) strategy. Once hydrolyzed inside cells, the modified uridines and cytidines are metabolically incorporated into nascent RNA, are labelled by a dual‐fluorogenic tetrazine‐dye to allow wash‐free, high‐contrast imaging of RNA synthesis in cells.

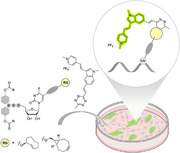

## Introduction

To gain a deeper understanding of cellular mechanisms, it is crucial to study biomolecules within their native cellular environment and elucidate their functions in biomolecular processes. However, the inherent complexity of cells makes this task challenging.^[^ ^1^ ^]^ Chemical biology aims to achieve this goal by utilizing bio‐orthogonal modification strategies.^[^ ^2^ ^]^ The fluorescence‐based labeling and imaging of nucleic acids represents a critical methodology for investigating the function and spatiotemporal dynamics of nucleic acids, especially RNA. In addition to their role in transcription and translation, non‐coding RNA performs essential catalytic and regulatory functions (e.g., microRNA, siRNA, tRNA, ncRNA, snRNA, eRNA, etc.)^[^ ^3^ ^]^ RNA in cells is conventionally labeled and imaged by fluorescence in situ hybridization (FISH)^[^ ^4^ ^]^ and copper‐catalyzed azide‐alkyne cycloaddition (CuAAC)^[^ ^5^ ^]^ which all involve extensive preparative steps and fixation of target cells. Live‐cell approaches such as MS2 tagging system,^[^ ^6^ ^]^ molecular beacons,^[^ ^7^ ^]^ hybridization‐sensitive nucleic acids,^[^ ^8^ ^]^ and aptamers^[^ ^9^, ^10^ ^]^ or CRISPR Cas13‐based imaging approaches^[^ ^11^ ^]^ label select RNA sequences and cannot be used to image overall RNA synthesis and trafficking. In contrast, bio‐orthogonal chemistry, when combined with metabolic labeling,^[^ ^12^, ^13^ ^]^ has the potential to label all newly synthesized RNA in living cells without the need for specific probes or complex genetic engineering approaches. It can be used in pulse‐chase protocols to follow RNA populations over time. Metabolic labeling of nucleic acids typically involves the incorporation of chemically modified nucleosides, which are enzymatically phosphorylated by endogenous kinases to form functional nucleoside triphosphates (NTPs). These NTPs are subsequently incorporated into newly synthesized RNA by RNA polymerases, enabling a range of analytical methods, including enrichment^[^ ^14^ ^]^ and visualization with a fluorescent probe.^[^ ^15^ ^]^ Unfortunately, high kinase substrate specificity often restricts the salvage pathway of modified nucleosides, causing inefficient phosphorylation and preventing RNA incorporation.^[^ ^16^, ^17^ ^]^ This drawback is especially acute for sterically demanding reporters that undergo fast bio‐orthogonal reactions. We recently demonstrated that the efficiency of metabolic labeling of DNA correlates with the size of the bio‐orthogonally reactive moiety.^[^ ^12^, ^17^ ^]^ Consequently, the practical application of nucleoside modifications is limited to a narrow range of very small functional groups with low reaction rate constants, as reactivity correlates with steric bulk.^[^ ^12^ ^]^ Beyond alkynyl‐modified nucleosides, such as 5‐ethynyl‐uridine (EU),^[^ ^5^ ^]^ azido‐modified nucleosides have also already been employed for intracellular RNA labeling by the copper‐catalyzed azide‐alkyne cycloaddition (CuAAC) and the strain‐promoted azide‐alkyne cycloaddition (SPAAC).^[^ ^18^, ^19^ ^]^

**Figure 1:**
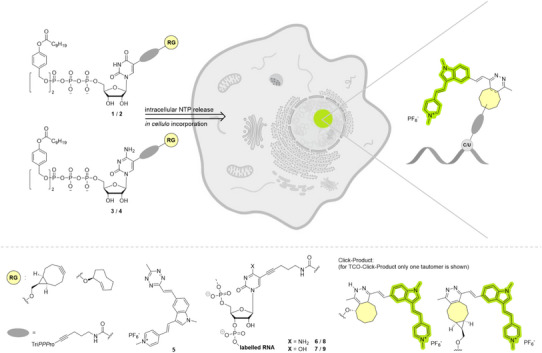
Overview of modified pronucleotide reporter (Tri*PPP*ro) used for imaging of newly synthesized cellular RNA in living cells. The Tri*PPP*ro delivery strategy involves intracellular hydrolysis of esterase‐sensitive lipophilic acyloxybenzyl (AB) protecting groups—a crucial modification for initial cell membrane permeability. The resulting nucleoside triphosphate (NTP) can then be incorporated into newly synthesized cellular RNA and subsequently labeled with a tetrazine‐fluorophore conjugate **5** in an IEDDA reaction. **1**, **3**, **6,** and **7** bearing a BCN‐modification. **2**, **4**, **8,** and **9** bearing 2TCOa as a reactive group (RG).

To overcome the cytotoxicity of copper(I) ions and the necessity for cell fixation, metal‐free alternatives, particularly the inverse electron‐demand Diels–Alder reaction (IEDDA), were introduced, thereby expanding the repertoire of RNA labeling techniques.^[^ ^20^ ^]^ In addition to its excellent orthogonality and biocompatibility, as well as its remarkably fast reaction rate constants, the IEDDA is attractive for fluorescence imaging if combined with fluorogenic tetrazine‐dye conjugates, allowing for wash‐free cell imaging with reduced unspecific background signal.^[^ ^21^, ^22^, ^23^, ^24^, ^25^ ^]^ Here, wash‐free refers to the fluorescence labeling step.^[^ ^21^ ^]^ The smallest chemical reporter known for the IEDDA reaction is the vinyl group in 5‐vinyl‐uridine (VU) and 7‐deaza‐7‐vinyl‐adenosine (VA), which has been previously utilized for metabolic labeling of RNA.^[^ ^26^ ^]^ However, the fastest IEDDA reactions are those with ring‐strained alkenes and alkynes, such as the bicyclo[6.1.0]nonyne (BCN) and *trans*‐cyclooctene (TCO).^[^ ^26^, ^27^ ^]^ Thus, reporters that give the best click rates are those least tolerated by the cellular kinase machinery. Several work‐arounds have been explored. Pre‐synthesized fluorescent NTPs or transporter‐assisted delivery (e.g., SNTT) bypass the kinase step but introduce new liabilities: constitutively fluorescent nucleotides raise background, and co‐administered transporters complicate protocols and can be cytotoxic if administered for too long.^[^ ^28^, ^29^, ^30^, ^31^, ^32^, ^33^, ^34^ ^]^ Moreover, no current method combines i) a transporter‐free route for TCO/BCN NTPs, ii) a dual fluorogenic turn‐on that avoids washing in the fluorescence labeling step, and iii) minimal cytotoxicity for RNA labeling. We recently introduced the Tri*PPP*ro (triphosphate‐prodrug) concept to metabolic labeling of nucleic acids, in which two esterase‐cleavable acyloxybenzyl (AB) groups mask the γ‐phosphate lypophilically, rendering the pronucleotide inherently cell‐permeable; enzymatic unmasking inside the cytosol liberates the active NTP, obviating any auxiliary transporter.^[^ ^35^, ^36^ ^]^ Here we combine the Tri*PPP*ro strategy with a next‐generation, dual‐fluorogenic tetrazine–cyanine‐styryl dye for RNA live‐cell imaging. In this system, Tri*PPP*ro ferries 2TCO*a*‐ and *endo*‐BCN‐modified uridine **1, 2** and cytidine **3, 4** analogues into living cells, where endogenous polymerases incorporate them into nascent RNA. The subsequent IEDDA click reaction with dye **5** triggers a two‐step fluorescence activation: i) the tetrazine quencher is converted into a non‐quenching pyridazine, and ii) nucleic‐acid binding further amplifies the signal, together producing bright, wash‐free fluorescence with low background. Using this integrated platform, which supplies the so‐far missing combination of bulky‐handle compatibility, transporter independence, and low background, we here demonstrate nascent RNA labeling in living cells (Figure 1).

## Results and Discussion

### Synthesis of 2TCO*a*‐ and *endo*‐BCN‐Modified Tri*PPP*ros and Triphosphates for RNA Labeling

We designed the RNA reporters based on the structure of the previously described 2′‐deoxycytidine analog^[^ ^36^ ^]^ and aimed to compare the efficiency of incorporating the modified cytidines and uridines. To anchor the IEDDA‐reactive bio‐orthogonal labeling site to the corresponding nucleotides, we introduced a universal 5‐amino‐1‐pentynyl linker to the nucleobase.^[^ ^37^ ^]^ This ensures a sufficient distance between the click functionality and the nucleic acid and is also known to be well accepted by various polymerases at the 5‐position of pyrimidines.^[^ ^38^, ^39^, ^40^, ^41^, ^42^, ^43^ ^]^ We employed the axial isomer of *trans*‐cyclooct‐2‐ene (2TCO*a*) and the *endo*‐isomer of bicyclo[6.1.0]non‐4‐yne (*endo‐*BCN) as IEDDA‐reactive reporters to enable a comprehensive comparison between these two frequently used clickable moieties. 2TCO*a* belongs to the well‐known *trans*‐cyclooctenes commonly used in IEDDA‐based bio‐orthogonal labeling. However, it exhibits a significantly improved stability against trans‐cis isomerization in the presence of biological thiols, while retaining high‐speed kinetics (*k*_2 _= 10^4^ M^−1^s^−1^) necessary for efficient labeling at biological concentrations.^[^ ^44^, ^45^ ^]^ Unfortunately, it is known that the click‐product of 2TCO*a* and 1,2,4,5‐tetrazines can be susceptible to a β‐elimination, contingent upon the tetrazines’ electronic structure, resulting in the dissociation of the coupled fluorophore from the nucleic acid host structure.^[^ ^46^, ^47^ ^]^ Further, the reaction between tetrazines and TCOs initially yields a non‐aromatic dihydropyridazine. It has been demonstrated that these dihydropyridazines may act as a quencher, resulting in the signal of fluorogenic turn‐on probes not being fully restored in some cases.^[^ ^48^ ^]^ BCN, on the other hand, is a versatile functional group that can undergo both IEDDA reactions with 1,2,4,5‐tetrazines and huisgen‐type azide‐alkyne cycloadditions.^[^ ^49^ ^]^ In contrast to TCOs, the reaction of strained alkynes and tetrazines directly gives the aromatic pyridazines, thus avoiding the possible quenching. However, the reaction rates with 3,6‐disubstituted methyl‐tetrazines are diminished, and the reduced stability of such strained alkynes should be considered.^[^ ^44^, ^50^ ^]^

**Scheme 1:**
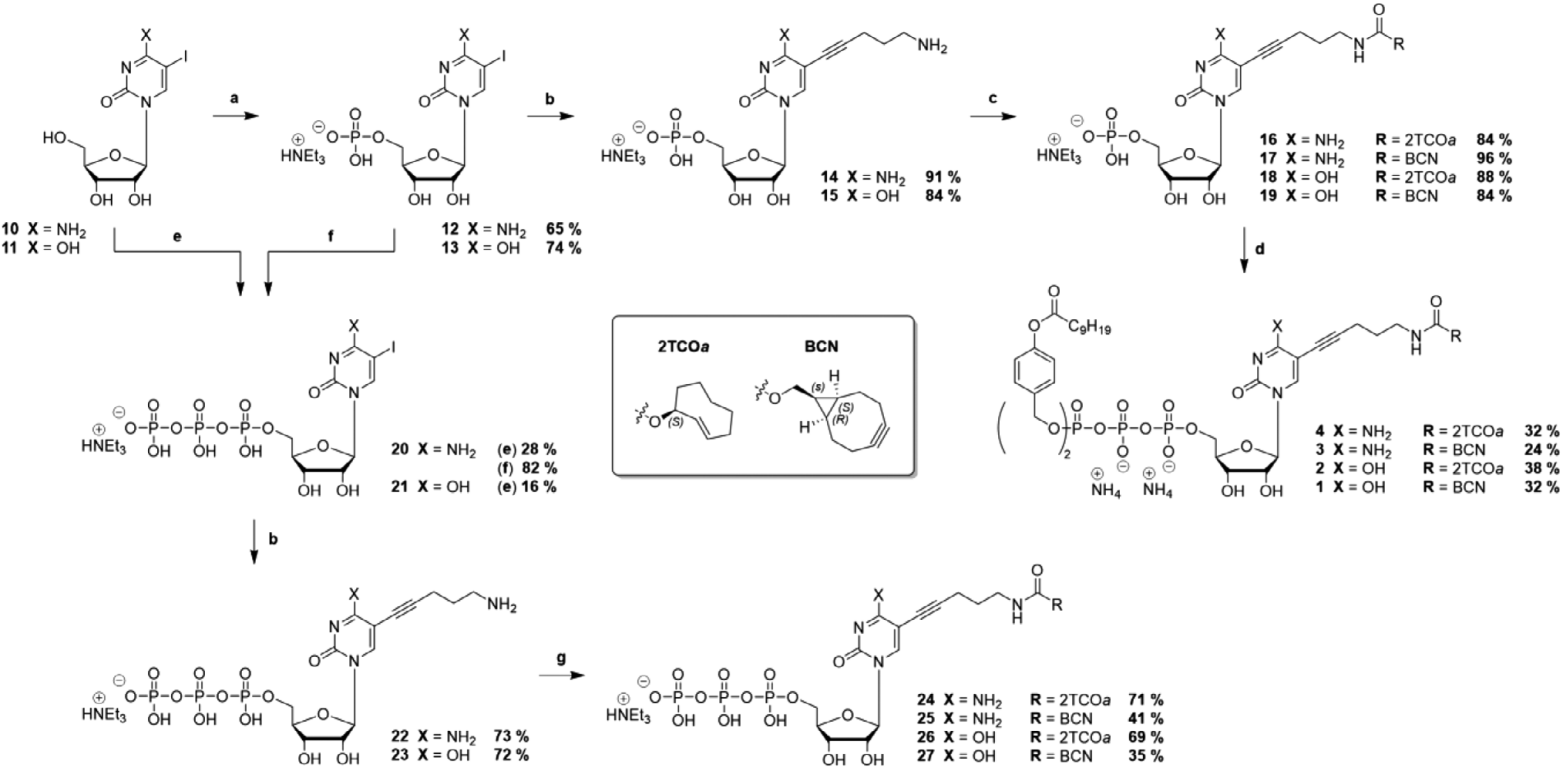
Synthesis of cytidine‐ and uridine‐based IEEDA‐reactive Tri*PPP*ro‐nucleotides **1**–**4** and triphosphates **24**–**27** for metabolic labeling of RNA. Reaction conditions: **a**. 4.8 eq. pyridine, 4.4 eq. POCl_3_, 2.0–2.5 eq. H_2_O, MeCN, 0 °C, 2–2.5 h **b**. 12 mol% tri(2‐furyl)phosphine, 5.4 mol% Pd_2_(dba)_3_, 15 mol% CuI, 7.0 eq. Et_3_N, 1.4 eq. pent‐4‐yn‐1‐amine, DMSO, 50 °C, 1 h, **c**. 1.15 eq. 2TCOa‐ or BCN‐NHS, 3.5 eq. Et_3_N, DMSO, rt, 1 h, **d**. (i) 1.0 eq. bis[C9AB]‐pyrophosphate, 5.0 eq. TFAA, 8.0 eq. Et_3_N, 0 °C – rt, 15 min, (ii) 5.0 eq. Et_3_N, 2.5 eq. NMI, DMF, rt, 15 min, (iii) 0.4–0.5 eq. monophosphate, rt, 2 h, **e**. (i) 1.2 eq. POCl_3_, TMP, 0 °C, (ii) 0.85 eq. (NBu_4_)_3_HP_2_O_7_, 6.0 eq. Bu_3_N, MeCN, 0 °C, 20 min, (iii) H_2_O, **f**. (i) 12 eq. Et_3_N, 10 eq. TFAA, MeCN, rt, 10 min, (ii) 10 eq. Et_3_N, 5.0 eq. NMI, MeCN, rt, 10 min, (iii) 3.0 eq. (NBu_4_)_3_HP_2_O_7_, MeCN, rt, 1 h, **g**. 1.15 eq. 2TCOa‐ or BCN‐NHS, 3.5 eq. Et_3_N, DMSO, 40 °C, 2 h.

The synthesis of the Tri*PPP*ro‐compounds started with the corresponding 5‐iodonucleosides **10** and **11**, which were selectively converted into their 5′‐*O*‐monophosphates **12** and **13** using a mixture of phosphoryl chloride, pyridine, and water in acetonitrile according to the Sowa & Ouchi protocol (Scheme 1).^[^ ^51^ ^]^ Subsequently, the nucleic acid linker was introduced under standard Sonogashira cross‐coupling conditions with the catalyst Pd_2_(dba)_3_, tri(2‐furyl)phosphine, and copper(I) iodide as co‐catalyst. At this point, protecting the primary amino function is unnecessary, but the free base can be used directly. The linker‐modified nucleotides **14** and **15** were isolated by RP_18_ flash column chromatography with a gradient of acetonitrile in 0.05 M TEAB buffer in very good to excellent yields of 84 % and 91 %. Buffer salts remaining from chromatography were removed by co‐evaporation of the obtained products with methanol and subsequent lyophilization. The dienophile motifs were then attached to the linker by reacting the amino‐functionalized monophosphates **14** and **15** with the commercially available NHS‐carbonates of 2TCO*a* and *endo*‐BCN. The Tri*PPP*ro reporters were then synthesized according to the previously published *H*‐phosphonate route.^[^ ^52^ ^]^ Accordingly, bis[C9AB]‐*H*‐phosphonate1 **35** was oxidatively chlorinated with NCS in acetonitrile at 50 °C and subsequently reacted with an excess of tetrabutylammonium dihydrogen phosphate. After removing all volatiles in an oil‐pump vacuum at room temperature, the corresponding pyrophosphate **36** was isolated by dissolving it in dichloromethane and subsequently washing with a 1 M aqueous ammonium acetate solution and water, using a centrifuge for faster phase separation. The synthesis typically yields more than 90%, and the obtained bis[C9AB]‐pyrophosphate **36** was used in the next step without further purification. It should be noted that the pyrophosphate is labile and, if not used directly, can be stored under a nitrogen atmosphere at ‐20 °C overnight or at ‐80 °C for up to three days without significant degradation. The coupling of the previously synthesized monophosphates **16**–**19** was finally carried out by the stepwise activation of the bis[C9AB]‐pyrophosphate **36** with trifluoroacetic anhydride (TFAA) and triethylamine, as well as *N*‐methylimidazole (NMI) and triethylamine, after which the monophosphates were added in one portion. The pronucleotides **1**–**4** were preliminarily purified by RP_18_ flash column chromatography and isolated with a sequence of ion exchange to ammonium counter ions (Dowex) and RP_18_ flash chromatography in 24%–38% yields. A complete conversion of the starting materials to the corresponding products could be observed by RP_18_ HPLC during the reaction, the moderate yields most probably originate from the elaborate isolation, as a repeated ion exchange is necessary to remove all unwanted counter‐ions.

To compare our approach with transporter‐based metabolic technique^[^ ^32^ ^]^ used in metabolic labeling of nucleic acid for the transmembrane delivery of fluorescent nucleotides and TCO‐ as well as BCN‐modified reporters for DNA labeling,^[^ ^33^, ^34^, ^53^, ^54^ ^]^ we also synthesized the corresponding triphosphates **24**–**27**. The 5‐iodonucleoside triphosphates **20** and **21** are available by either transforming the nucleosides directly in a Ludwig–Eckstein synthesis^[^ ^55^ ^]^ or via a coupling of the monophosphates **12** and **13** with tris(tetrabutylammonium) hydrogen pyrophosphate according to a modified procedure by Mohamady,^[^ ^56^ ^]^ with the latter usually leading to better yields. Analogously to the monophosphates, the linker was introduced to **20** and **21** via Sonogashira cross‐coupling, and the click motifs were attached to **22** and **23** via NHS‐chemistry. However, the last step needed elevated temperatures to complete the consumption of the starting material. Finally, we synthesized the green fluorescent tetrazine‐modified cyanine‐styryl dye **5** according to published protocols.^[^ ^57^ ^]^

### Dual Fluorogenic Activation and Kinetic Profiling of Tetrazine Click Reactions for Live‐Cell RNA Labeling

**Figure 2:**
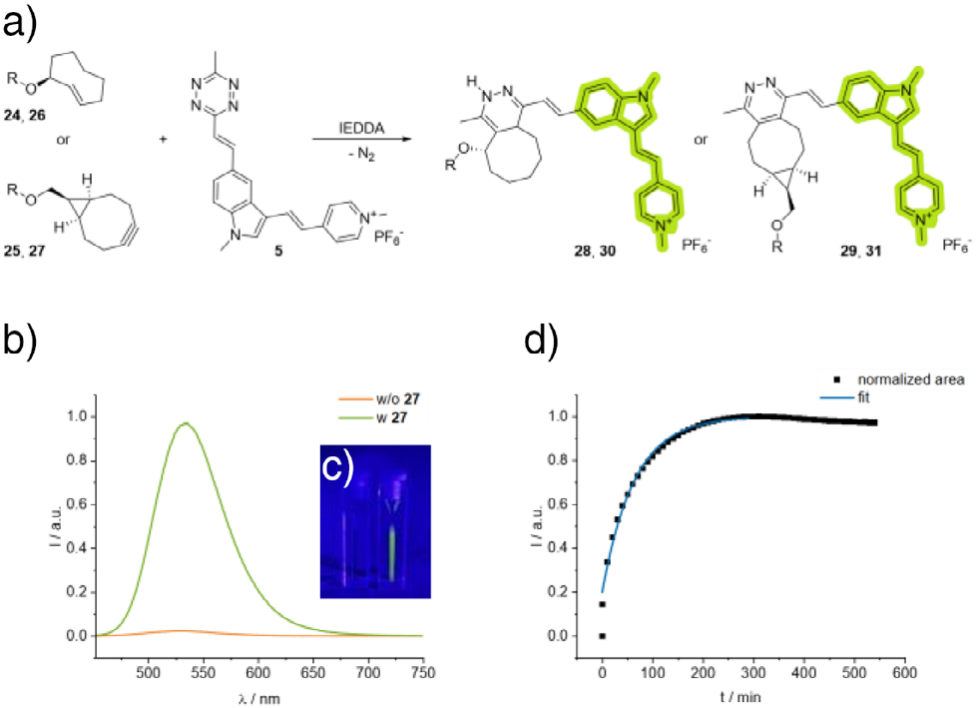
a) Schematic representation of the IEDDA reaction of *endo*‐BCN‐ and 2TCO*a*‐modified nucleoside triphosphates (NTP) **24**–**27** and the tetrazine‐fluorophore conjugate **5** under release of nitrogen. b) Increase of fluorescence intensity during the IEDDA reaction of BCN‐modified UTP **27** (200 µM, 10.0 eq.) and dye **5** (20 µM, 1.00 eq.) in water with 1% DMSO (*λ*_exc._= 437 nm, *λ*_emi._= 452–800 nm, slits: 2.5 nm, 20 °C). c) Visible turn‐on effect: **5** without click partner (left cuvette) and with click partner **27** (right cuvette). d) The kinetic plot displays the integrated fluorescence intensity as a function of time; exponential fit function *y* = *a* + *b* exp(‐*k*_obs_·x). R = CTP for **24**, **25**, **28,** and **29**. R = UTP for **26**, **27**, **30,** and **31**.

We have demonstrated previously that the conjugation of a cyanine‐styryl dye to a tetrazine results in a two‐factor fluorogenicity upon reaction with BCN‐modified nucleic acids.^[^ ^57^ ^]^ The observed two‐factor fluorogenicity results from both the conversion of the tetrazine to the pyridazine during the IEDDA reaction and sensitivity by non‐covalent interactions with nucleic acids.^[^ ^57^, ^58^ ^]^ The inherent improvement in signal‐to‐noise ratio makes this dye advantageous for live‐cell imaging and metabolic labeling, as it eliminates the requirement for washing steps before imaging. Accordingly, we used time‐dependent fluorescence spectroscopy to monitor the IEDDA reaction between the tetrazine‐dye conjugate **5** and the 2TCO*a*‐ and BCN‐modified parent nucleotides in aqueous solution (Figures 2 and S1–S4). These NTP reactions exhibited fluorescence turn‐on values ranging from 31 to 45, showing no significant difference between the respective reporters (Table 1). The higher intrinsic ring strain of 2TCO*a*, relative to BCN, culminates in a higher reactivity as a dienophile in IEDDA reactions.^[^ ^59^ ^]^ Consistently, the 2TCO*a*‐modified nucleotides **24** and **26** displayed slightly increased second‐order rate constants (Table 1). Based on our previous kinetic studies with DNA, which resulted in turn‐on values up to 560 and rate constants up to *k*_2_ = 284000 M^−1^s^−1^, we anticipate significantly higher fluorescence turn‐on values with single‐stranded and double‐stranded RNA.^[^ ^57^ ^]^ We postulate that the reaction of TCO‐ and BCN‐modified RNA with dye **5** should exhibit reaction kinetics in a similar range.

**Table 1:** Fluorescence turn‐on values and second‐order rate constants *k*_2_ for the IEDDA reaction of the nucleotides **24**–**27** with the tetrazine‐modified dye **5**.

NTP	Product	Fluorescence turn‐on	*k*_2_ (M^−1^s^−1^)
**24**	**28**	45	2.3
**26**	**30**	31	8.7
**25**	**29**	32	1.1
**27**	**31**	36	1.5

In contrast to the BCN‐modified analogues **25** and **27**, the kinetic profile of the 2TCO*a*‐modified compounds **24** and **26** exhibits two successive signal increases, with a transient decrease in fluorescence (Figures S3 and S4). The successive increase could be attributed to the two‐step mechanism of the IEDDA reaction between 2TCO*a* and dye **5**. Initially, a non‐aromatic dihydropyridazine intermediate is formed, which subsequently undergoes oxidative aromatization to the pyridazine product.^[^ ^27^ ^]^ Additionally, it is known that ß‐elimination can occur in association with 2TCO*a*,^[^ ^46^ ^]^ which likely accounts for the transient decrease in the kinetic profiles of nucleotides **24** and **26**, as dissociation of the fluorophore from the reporter moiety reduces the fluorescence turn‐on caused by steric bulk in close proximity. Once oxidation to the aromatic pyridazine has taken place, ß‐elimination is no longer possible, and partial quenching by the dihydropyridazine moiety is impeded, resulting in a further increase in the fluorescence signal. Mass analysis of the reaction mixture of **24** and **5** confirmed the formation of the elimination product as well as the formation of the aromatic pyridazine product **28**. Further, kinetic analysis of the click reaction between the non‐eliminating 4TCO and dye **5** revealed a two‐step increase in fluorescence without an intermediate decrease, further supporting our assumption (Figure S5).

Moreover, we tested the extent to which the previously described β‐elimination using the 2TCO*a* click‐motif plays a role.^[^ ^46^ ^]^ Therefore, we used the published fluorogenic coumarin probe **39**, which lights up upon elimination of the parent 7‐amino‐4‐methylcoumarin **37** from the TCO moiety. By reacting the 2TCO*a*‐coumarin with an excess of tetrazine **5** in PBS/DMSO, we could determine the extent of elimination to be around 5% after 3 h (Figure S7), suggesting that the electronic structure of our tetrazine‐dye conjugate does not strongly favor an unwanted dissociation of the fluorescent probe from the incorporated nucleotide reporters **2** and **4**. Based on our kinetic analyses, on the other hand, click‐products **28** and **30** display a fluorescence decrease of 5%–17%, which advocates for the coumarin probe **39** to underestimate the actual extent of β‐elimination. However, it should be considered that once the dihydropyridazine intermediate is oxidized within the cellular environment, elimination of the fluorophore is not possible. Furthermore, the dissociation of the cyanine‐styryl dye is accompanied by a decrease in fluorescence, thereby reducing the potential for off‐target labeling.

### Tri*PPP*ro Facilitates High‐Efficiency RNA Labeling

**Figure 3:**
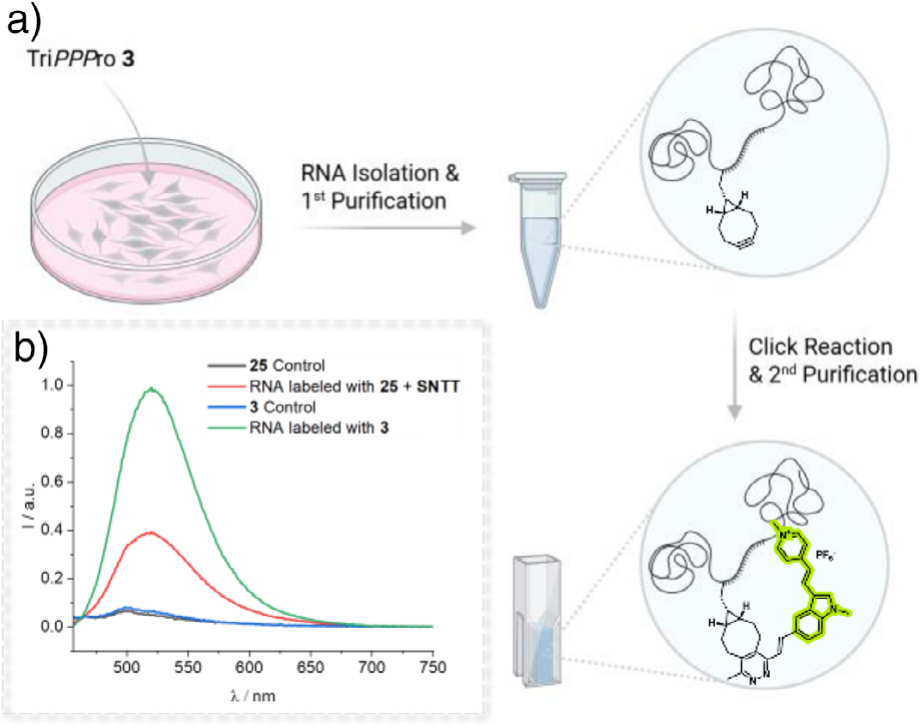
Fluorescence spectroscopic analysis of isolated RNA. a) Schematic workflow of the RNA isolation after treatment of Vero cells with 5 µM compound **3** in VP‐SFM at 37 °C for 4 h (Refreshment of Tri*PPP*ro solution after 2 h). Cells were lysed and RNA was extracted using TRIzol. RNA purification from aqueous phase after TRIzol extraction was performed using RNA Clean and Concentrator‐5 Kit. As a control group, RNA was extracted from Vero cells incubated in VP‐SFM. For the click reaction, isolated RNA was incubated with 5 µM of dye **5** at room temperature (rt) overnight (water with 1% DMSO) and subsequently purified using the same spin column‐based method. b) Fluorescence spectra of RNA‐labeled with compound **3** (green) or **25** and SNTT after click‐reaction with dye **5**, compared to unlabeled control RNA (black for **25 + SNTT**, blue for **3**). *λ*_exc_ = 437 nm, *λ*_em_ = 452–800 nm, 20 °C. Experiment was performed in duplicate. Created with BioRender.com.

Following the analysis of the click reaction, we evaluated the Tri*PPP*ro technique for its ability to enable metabolic incorporation of 2TCO*a*‐ and BCN‐modified nucleotide reporters into cellular RNA. Vero cells were incubated with 5 µM Tri*PPP*ro‐compound **3** for 4 h in serum‐free VP‐SFM to prevent premature cleavage of the AB‐masked phosphates by carboxyesterases or lipases. As a negative control, cells were treated with the corresponding amount of DMSO in VP‐SFM. No cytotoxicity was observed, in line with previous results from DNA labeling.^[^ ^36^ ^]^ Total RNA was extracted via TRIzol‐chloroform and purified using the Zymo Clean and Concentrator‐5 Kit (Figure 3a). The RNA was then subjected to an IEDDA click reaction with 5 µM dye **5** in water containing 1% DMSO and incubated overnight. After removal of unreacted dye through an additional purification step, fluorescence spectroscopy revealed a strong signal for RNA labeled with BCN‐modified compound **3**, confirming successful metabolic incorporation and demonstrating the effectiveness of the Tri*PPP*ro approach for RNA labeling (Figure 3b).

To compare Tri*PPP*ro against a transporter approach, we evaluated the SNTT system. Vero cells were treated with an equimolar solution of BCN‐modified nucleotide **25** and SNTT (60 µM) in tricine buffer for 10 min, as recommended to minimize cytotoxicity,^[^ ^32^ ^]^ followed by a 4‐h incubation in complete medium. RNA extraction and click labeling with dye **5** were performed as described above. While fluorescence spectroscopy confirmed successful incorporation of compound **25** via SNTT‐mediated delivery, the observed signal was markedly lower, indicating reduced labeling efficiency under these conditions (Figure 3b).

### Tri*PPP*ro with Dual‐Fluorogenic Dye Enables Low Background Labeling of Newly Synthesized RNA in Living Cells

**Figure 4:**
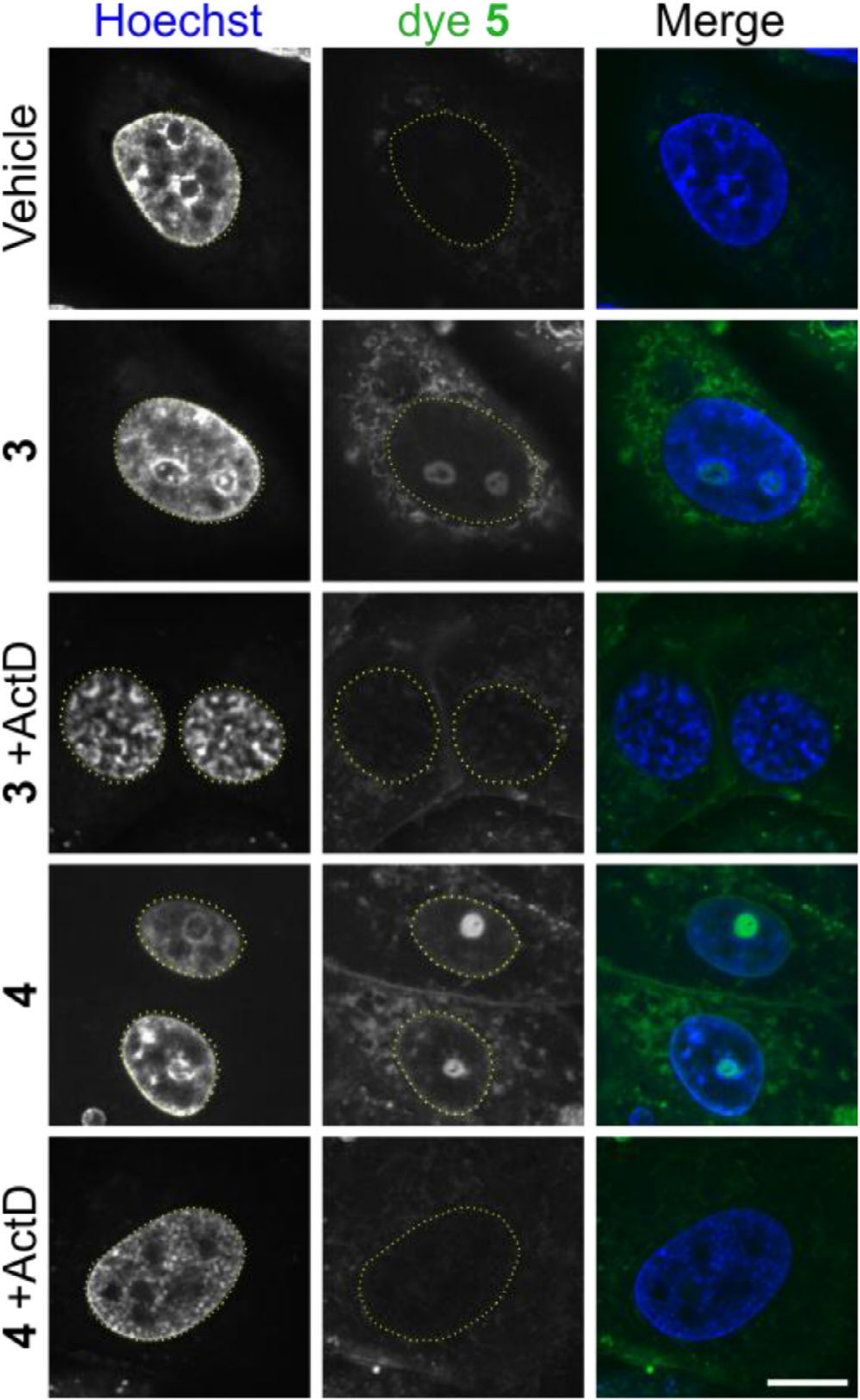
Detection of nascent RNA using *endo*‐BCN‐ and 2TCO*a*‐Tri*PPP*ro‐nucleotides 3 and 4. Vero cells were washed with VP‐SFM and incubated with 5 µM of the indicated Tri*PPP*ro nucleotide, with or without the transcription inhibitor actinomycin D (ActD). After labeling, cells were washed three times with VP‐SFM, and ActD was reapplied where indicated. After 30 min incubation with 5 µM tetrazine‐fluorophore conjugate **5** to visualize Tri*PPP*ro‐tagged RNA (green) and Hoechst (blue) to label nuclei, live‐cell imaging was performed by using a Nikon Ti2 spinning disk fluorescence microscope equipped with a 100 × Apo TIRF objective. Dye **5** was excited with the 488 nm laser line (80% intensity, 600 ms exposure time), while Hoechst was excited with the 405 nm laser line (50% intensity, 200 ms exposure time). Nuclear outlines are circled (yellow). Individual channels are shown in grayscale; the merged image is shown in color. Scalebar, 10 µm. Representative images of at least 2 biological replicates.

Next, we tested the efficacy of our Tri*PPP*ro RNA approach for labeling newly synthesized RNA in living cells. We first incubated Vero cells for 3 h with 5 µM 2TCO*a*‐ or BCN‐modified Tri*PPP*ro‐CTPs **3** and **4** in VP‐SFM. Unincorporated nucleotides were then washed away, and the tagged RNA was visualized with the fluorogenic tetrazine‐cyanine styryl dye **5**, while nuclei were counterstained with Hoechst 33 342. We found that cells exposed only to dye **5** displayed low background fluorescence, demonstrating minimal nonspecific labeling. By contrast, cells treated with either 2TCO*a*‐ or BCN‐Tri*PPP*ro‐CTPs **3** and **4** showed robust fluorescence confined to distinct intranuclear foci. Transcriptional inhibition with Actinomycin D (ActD) abolished this signal, confirming that it derives from nascent RNA (Figure 4).

We also used the Mitochondria‐specific probe MitoTracker and could confirm that the cytoplasmic Tri*PPP*ro‐signal colocalizes with mitochondrial labeling (Figure S8), indicating that the observed cytoplasmic signal is mainly due to labeled mitochondrial RNA. The weak residual cytoplasmic signal observed in the ActD‐treated control might be attributed to the residual accumulation of the positively charged dye in mitochondria due to the membrane potential or to incomplete inhibition of mitochondrial transcription^[^ ^60^ ^]^ (Figures 4 and S8). Labeled cellular RNA could be followed for at least 48 h using lattice light‐sheet microscopy, indicating that our labeling approach allowed long‐term tracking of long‐lived RNAs with minimal to no cytotoxicity (Figure S9).

**Figure 5:**
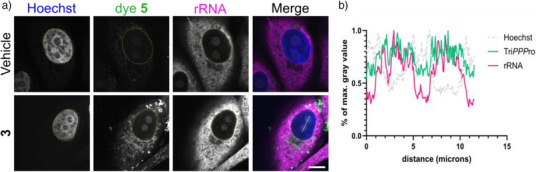
Nascent RNA labeling and ribosomal RNA (rRNA) colocalization in Vero cells. a) Confluent Vero cells were washed with VP‐SFM and incubated with 5 µM 2TCOa‐Tri*PPP*ro **3** for 3 h to tag newly synthesized RNA. Cells were subsequently washed three times in VP‐SFM and labeled with a 5 µM tetrazine‐fluorophore conjugate **5** (green) for 1 h, while simultaneously being stained with Hoechst (blue). Following fixation and permeabilization, rRNA was detected by immunofluorescence (magenta). Imaging was performed using a Nikon Ti2 spinning disk fluorescence microscope equipped with a 100 × Apo TIRF objective. Dye **5** was excited with the 488 nm laser line (80% intensity, 600 ms exposure time), Hoechst with the 405 nm laser line (50% intensity, 200 ms exposure time), and Alexa Fluor 647 with the 638 nm laser line (75% intensity, 200 ms exposure time). Nuclear outlines are circled (yellow). Individual channels are shown in greyscale, the merged image is shown in color. Scalebar, 10 µm. b) The line profile analysis (yellow line) illustrates the colocalization of nascent RNA (Tri*PPP*ro, green) with rRNA (magenta) signals in the nucleoli within the nucleus.

Colocalization with 5.8S RNA confirmed that intranuclear fluorescent foci correspond to nucleoli, an RNA‐rich nuclear compartment (Figure 5). This observation parallels earlier studies on 5‐ethynyl‐uridine,^[^ ^5^ ^]^ in which EU incorporation accumulated in nucleoli and overlapped with rRNA markers, further validating that our clickable nucleotides are efficiently incorporated into newly transcribed rRNA. Quantification of nucleolar signal intensities confirmed a significant increase for all Tri*PPP*ro derivatives (**1**–**4**) compared to vehicle controls, with no discernible differences among the individual analogues (Figures S10 and S11). We further investigated labeling dynamics of the metabolic incorporation to assess the efficiency of our approach in terms of cellular uptake and delivery of the nucleoside triphosphate reporters. Clear labeling of the nucleoli was already discernible after 30 min of incubation with Tri*PPP*ro **3**, yielding a significant fluorescence signal compared to the negative control (Figure S12). These findings indicate rapid transmembrane delivery and metabolic liberation of the reporter molecules using our Tri*PPP*ro approach, providing methodological flexibility for optimization to a wide range of biological questions. Additionally, we could show that our approach is also applicable in mammalian cell lines from different species (Figure S13). In contrast, SNTT‐mediated labeling, even with 60 µM of the BCN‐modified nucleotide **25** for the recommended time did not lead to any specific labeling with dye **5** (Figures S14A and S15), indicating that the proportion of incorporated BCN tag that remains accessible for the click reaction *in cellulo* is limited. Labeling of nascent RNA was only discernible when using a pre‐clicked NTP derivative **29** (Figure S14B), implying that the efficiency of fluorescence labeling is a product of both the cellular uptake and click labeling efficiency in the complex cellular environment.

Taken together, these results demonstrate that the clickable Tri*PPP*ro‐nucleotide analogs in conjunction with dual‐turn‐on dyes offer a robust, specific, and sensitive approach for visualizing and characterizing nascent RNA synthesis and their dynamics in live‐cell imaging applications, with strong potential for further biological and biochemical studies.

## Conclusion

We address three classic obstacles to live‐cell RNA imaging—delivery, specificity, and signal‐to‐noise—by combining transporter‐free nucleotide entry via the Tri*PPP*ro system, a highly modular chemical design, and a dual‐fluorogenic read‐out in a single workflow. Masking the triphosphate with biolabile acyloxybenzyl groups renders *endo*‐BCN‐ and 2TCO*a*‐modified nucleotides cell‐permeant; endogenous esterases then liberate the active NTP without the need for auxiliary transporters. Coupling these reporters with a red‐shifted tetrazine–cyanine‐styryl dye, applied here for the first time in live‐cell metabolic labeling, results in two independent fluorogenic switches—loss of tetrazine quenching and steric unquenching upon nucleic‐acid binding resulting in minimal fluorescence background. The result is a wash‐free, high‐contrast signal which is ideal for live‐cell imaging. Moreover, utilizing the green–red excitation window avoids the phototoxic 405 nm light required for intrinsically fluorescent base analogues. Because the masking group, the nucleobase, and the bioorthogonal handle can be exchanged independently, the platform is inherently modular and readily extendable to adenosine, guanosine, or emerging click motifs. Together, these attributes make this approach a genuinely practical tool for real‐time visualization of bulk RNA synthesis in living cells. Its efficiency, chemical flexibility, and clean imaging window promise straightforward extensions to diverse cellular models, and viral systems, opening new avenues for dissecting RNA dynamics with unparalleled spatiotemporal resolution.

## Supporting Information

The authors have cited additional references within the Supporting Information.^[^ ^36^, ^46^, ^51^, ^55^, ^56^, ^61^, ^62^ ^]^

All datasets generated and analyzed in this study are publicly available via the Zenodo repository. The complete dataset can be accessed via Zenodo using the following DOIs: 10.5281/zenodo.16411157 and 10.5281/zenodo.17288959.

## Conflict of Interests

The authors declare no conflict of interest.

## Supporting information



Supporting Information

## Data Availability

The data that support the findings of this study are openly available in Zenodo at 10.5281/zenodo.16411157 and 10.5281/zenodo.17288959, reference number 16411157.
