# Supplementary material for: Live‐Cell RNA Imaging via Clickable TriPPPro Nucleotide Reporters
Source: Angew Chem Int Ed Engl. 2025 Nov 27;65(2):e16613. doi: 10.1002/anie.202516613 (PMC12790367; doi:10.1002/anie.202516613)
Supplement: Supplementary file 1 — Supporting Information [file ANIE-65-e16613-s001.pdf]

Supporting Information

Table of Content

1. General Materials and Methods 4

1.1 Solvents and Reagents 4

1.2 Thin-Layer Chromatography (TLC) 4

1.3 Preparative Thin-Layer Chromatography (pTLC) 4

1.4 Column Chromatography 4

1.5 Flash Column Chromatography 4

1.6 Nuclear Magnetic Resonance Spectroscopy (NMR) 4

1.7 ESI Mass Spectrometry 4

1.8 MALDI Mass Spectrometry 5

1.9 Reversed Phase High-Performance Liquid Chromatography (RP_{18}-HPLC) 5

1.10 Ion Exchange with Dowex NH_4^+ 5

1.11 TEAB Buffer 5

1.12 Tricine Buffer 5

1.13 Determination of nucleic acid concentration 5

1.14 Fluorescence spectroscopy 5

1.15 UV/Vis Spectroscopy 6

1.16 Centrifugation 6

2. Synthesis 7

2.1 General Procedures 7

2.1.1 Synthesis of 5'-Monophosphates (General Procedure 1) 7

2.1.2 Synthesis of 5'-Triphosphates (General Procedure 2) 7

2.1.3 Linker Coupling (General Procedure 3) 7

2.1.4 Carbamate Formation (General Procedure 4) 8

2.1.5 Tri*PPP*ro Synthesis (General Procedure 5) 8

2.2 Synthesis of Tri*PPP*ros 9

2.2.1 Preparation of ICMP (12) 9

2.2.2 Preparation of IUMP (13) 9

2.2.3 Preparation of 5-phthalimidopent-1-yn (33) 10

2.2.4 Preparation of pent-4-yn-1-amine (34) 10

2.2.5 Preparation of AP-CMP (14) 11

2.2.6 Preparation of AP-UMP (15) 11

2.2.7 Preparation of 2TCO*a*-CMP (16) 12

2.2.8 Preparation of 2TCO*a*-UMP (18) 12

2.2.9 Preparation of BCN-CMP (17) 13

2.2.10 Preparation of BCN-UMP (19) 14
2.2.11 Preparation of Bis[C9AB]-pyrophosphate (36) 14
2.2.12 Preparation of Bis[C9AB]-2TCO*a*-CTP (4) 15
2.2.13 Preparation of Bis[C9AB]-2TCO*a*-UTP (2) 16
2.2.14 Preparation of Bis[C9AB]-BCN-CTP (3) 16
2.2.15 Preparation of Bis[C9AB]-BCN-UTP (1) 17
2.3 Synthesis of Triphosphates 19
2.3.1 Preparation of ICTP (20) 19
2.3.2 Preparation of IUTP (21) 20
2.3.3 Preparation of AP-CTP (22) 20
2.3.4 Preparation of AP-UTP (23) 21
2.3.5 Preparation of 2TCO*a*-CTP (24) 22
2.3.6 Preparation of 2TCO*a*-UTP (26) 22
2.3.7 Preparation of BCN-CTP (25) 23
2.3.8 Preparation of BCN-UTP (27) 23
2.4 Synthesis of TCO-Probe 24
2.4.1 Preparation of 7-isocyanato-4-methylcoumarin (38) 24
2.4.2 Preparation of 2TCO*a*(4-methylcoumarin-7-yl)carbamate (39) 24
3. Cell Experiments 26
3.1 Cell Culture 26
3.2 Procedures 26
3.2.1 Procedure for Isolation of RNA labeled with Tri*PPP*ro 3 and NTP 25 26
3.2.2 Procedure for the Click Reaction, following RNA-Isolation 26
3.3.3 Procedure for Fixed Cell Labeling with NTPs 26
3.3.4 Procedure for Fixed Cell Labeling with preclicked-NTPs 27
3.3.5 Procedure for Live Cell Labeling with Tri*PPP*ros 27
3.3.6 Procedure for rRNA Counterstain 28
3.3.7 Procedure for Live Cell Labeling with MitoTracker DeepRed™ 28
3.3.8 Procedure for Live Cell Labeling with Tri*PPP*ro vs. SNTT transporter 28
3.3.9 Procedure for Live Cell Labeling with Tri*PPP*ro with Lattice Lightsheet 28
3.3.10 Statistical analysis 28
4. Supplementary Figures 29
4.1 Turn-on values and kinetic studies 29
4.1.1 Click compound 31 (BCN-UTP 27 + 5) 30
4.1.2 Click compound 29 (BCN-CTP 25 + 5) 31
4.1.3 Click compound 30 (2TCO*a*-UTP 26 + 5) 32

4.1.4 Click compound 28 (2TCO*a*-CTP 24 + 5) 33
4.1.5 Click reaction 4TCO-NHS-carbonate + 5 34
4.2 Elimination Assay 34
4.4 Cell Images 36
5. Spectra 44
6. References 74

1. General Materials and Methods

1.1 Solvents and Reagents

Dry solvents were purchased from *Acros Organics* (Extra Dry over Molecular Sieve) and *Fisher Scientific* (Extra Dry over Molecular Sieve). Triethylamine and 1-methylimidazole were purchased from *Sigma Aldrich* (SureSeal). All other reagents were purchased from *BLD Pharm, Carl Roth, Fisher Scientific, Merck, Sigma-Aldrich, Tokyo Chemical Industry (TCI).* Water was deionized with a *Sartorius* Arium® pro unit (Sartopore 0.200 µm, UV). Stereochemically pure click reagents were purchased from *Sirius Fine Chemicals*. Commercially obtained reagents were used without further purification.

1.2 Thin-Layer Chromatography (TLC)

TLC was performed on pre-coated Alugram® Xtra SIL G/UV254 TLC plates (*Macherey-Nagel*). UV-active substances were visualized under the UV lamp at 254 and 365 nm. Further, compounds were stained with vanillin reagent (500 mg vanillin, 100 mL MeOH/AcOH 9:1 v/v, 3.50 mL H_2SO_4), Hanessian's stain (5.00 g ammonium molybdate, 1.00 g ceric sulfate, 10.0 mL H_2SO_4, 90.0 mL H_2O), or ninhydrin reagent (3.00 g ninhydrin in 200 mL EtOH) under heating.

1.3 Preparative Thin-Layer Chromatography (pTLC)

pTLC was carried out using pre-coated TLC plates (20×20 cm, 1.00 mm silica gel 60 layer with fluorescent indicator UV254, *Macherey-Nagel*).

1.4 Column Chromatography

Silica gel column chromatography was performed with silica gel MN 60 M (0.04 - 0.063 mm, *Macherey-Nagel*).

1.5 Flash Column Chromatography

RP_{18} flash column chromatography was performed with MN RS 43 g/120 g C_{18} ec columns (*Macherey-Nagel*) on a Puriflash 430 system (*Interchim*) or on an Isolera™ Systems (*Biotage®*). For each counterion (ammonium, triethylammonium, tetrabutylammonium) an individual column was used.

NP flash column chromatography was conducted using silica gel MN 60 M (0.04 - 0.063 mm, *Macherey-Nagel*) in self-packed cartridges (*Interchim*).

1.6 Nuclear Magnetic Resonance Spectroscopy (NMR)

NMR spectra were measured at the Institute of Organic Chemistry, University of Hamburg, and the Analytical Department of the Institute of Organic Chemistry, Karlsruhe Institute of Technology (KIT). The NMR spectra were recorded at room temperature (rt) on *Bruker* Avance III HD 400 MHz, *Bruker* Avance Neo 400 MHz, *Bruker* Avance I 500 MHz, or *Bruker* Avance III HD 600 MHz spectrometers. The chemical shifts (δ) were quoted in parts per million [ppm] and calibrated to the internal solvent signals. For further characterization, two-dimensional NMR experiments (H, H-COSY, HSQC, HMBC) were measured. NMR solvents were purchased from *Eurisotop* and *Deutero*.

1.7 ESI Mass Spectrometry

ESI mass spectrometry was performed at the Institute of Organic Chemistry, University of Hamburg, and in the Analytical Department of the Institute of Organic Chemistry at the Karlsruhe Institute of Technology (KIT). The chromatograms were recorded using an Agilent 6224 ESI-TOF spectrometer or a Q Exactive Plus Orbitrap mass spectrometer from *Thermo Scientific* in positive or negative mode.

1.8 MALDI Mass Spectrometry

MALDI mass spectrometry was performed in the Analytical Department of the Institute of Organic Chemistry at the Karlsruhe Institute of Technology (KIT) using an AXIMA Confidence spectrometer from *Shimadzu*. The matrix used was a saturated solution of 2',4',6'-Trihydroxyacetophenone monohydrate (THAP) in 50:50:0.1 Water:Acetonitrile:TFA.

1.9 Reversed Phase High-Performance Liquid Chromatography (RP_{18}-HPLC)

The high-performance liquid chromatography was carried out on a 1260 Infinity II LC-device (pump G7111A, autosampler G7129A, detector DAD G7117C, *Agilent Technologies*) with an EC 125/3 Nucleodur 100-5 C_{18} ec column/EC 4/3 Nucleodur 100-5 C_{18} ec pre-column system (*Macherey-Nagel*) and OpenLAB CDS as software. Method: a MeCN gradient in tetrabutylammonium acetate buffer (2.00 mM in H_2O, pH 6.00), 5 – 80%, 0 – 20 min (flow: 1.00 mL/min).

1.10 Ion Exchange with Dowex NH_4^+

For ion exchange to NH_4^+ counterions, a short column was packed with Dowex® 50W X8 (hydrogen form, 16-50 mesh) and flushed with H_2O until the eluted solvent stayed colorless. The column was then flushed with hydrochloric acid (1.00 M) and subsequently washed with H_2O until a neutral pH was reached. Then, the column was flushed with ammonia (10% in H_2O) and washed with H_2O until the pH was neutral again. The corresponding compound was dissolved in H_2O (in case of low solubility, some MeCN was added) and applied to the column. The compound was subsequently eluted with several column volumes of H_2O and lyophilized. This process was repeated for each ion exchange.

1.11 TEAB Buffer

Triethylammonium bicarbonate buffer was produced by bubbling CO_2 (3 kg) into a stirred dispersion of triethylamine in H_2O (1.00 M) overnight. The resulting buffer (1.00 M, pH 7.40) was diluted accordingly.

1.12 Tricine Buffer

For washing and RNA-labeling treatment of cells, a tricine buffer was used. The buffer contains following ingredients: tricine (0.896 g/L, 5.00 mmol/L), glucose (2 g/L, 11.0 mmol/L), NaCl (7.325 g/L, 125 mmol/L), $CaCl_2$ (0.200 g/L, 1.80 mmol/L), $MgSO_4$ (0.100 g/L, 0.800 mmol/L), KCl (0.400 g/L, 5.40 mmol/L). The buffer pH was adjusted with NaOH to 7.40 at 31.0 °C.

1.13 Determination of nucleic acid concentration

Determination of the concentration of isolated RNA was performed on a NanoDrop ND-100 spectrometer from *Fisher Scientific.* For this, the nucleic acid mode at 260 nm was used, with a sample volume of 1 µL applied.

1.14 Fluorescence spectroscopy

Fluorescence spectroscopic measurements were performed using a Fluoromax-4 fluorescence spectrometer equipped with a Peltier element from *Horiba Scientific* (formerly *Jobin-Yvon*) at 20 °C. Background interference in the fluorescence signal was corrected by subtracting the solvent spectrum. Wavelength accuracy of the spectrometer was ensured by calibration against the Raman scatter of water. Quartz spectrophotometer cuvettes from *Starna* with a 10 mm path length and a sample capacity of 0.50 mL were used.

1.15 UV/Vis Spectroscopy

UV/Vis spectroscopic measurements were performed using a Cary3500 Multicell UV/Vis spectrometer with Peltier element from *Agilent*. The samples were measured in 1 cm quartz glass cuvettes (*Starna*) with a volume of 0.50 mL at 20 °C.

1.16 Centrifugation

Centrifugation was performed using a *VWR* Micro Star 17R microcentrifuge with integrated cooling. Samples were centrifuged at 12,000 x g unless stated otherwise.

2. Synthesis

All reactions were carried out under a nitrogen or argon atmosphere using dry solvents.

2.1 General Procedures

2.1.1 Synthesis of 5'-Monophosphates (General Procedure 1)

The monophosphates were synthesized according to a previously published procedure by SOWA & OUCHI.[1]

Pyridine (4.80 equiv) was added dropwise to a 0 °C cold solution of phosphoryl chloride (4.40 equiv) in MeCN (19.0 equiv). H_2O (2.00 – 2.50 equiv) was slowly added dropwise (Caution: phosphoryl chloride reacts violently with H_2O), and the resulting mixture was stirred for an additional 10 min at 0 °C. After adding the corresponding 5-iodonucleoside (1.00 equiv) in one portion, the reaction progress was followed by RP_{18}-HPLC. At full conversion or increasing occurrence of unwanted by-products, the reaction was hydrolyzed by pipetting into ice-cold H_2O, and the pH was adjusted to 8 by the careful addition of ammonium bicarbonate. The resulting mixture was lyophilized and then subjected to RP_{18} flash chromatography (10 min H_2O, then gradually to MeCN over 30 min). All fractions containing the product were combined, and triethylamine (10.0 equiv) was added. After stirring for 30 min at rt, the mixture was concentrated under reduced pressure and then subjected to RP_{18} flash chromatography.

2.1.2 Synthesis of 5'-Triphosphates (General Procedure 2)

The triphosphates were synthesized according to a previously published procedure by KORE.[2]

5-iodonucleoside (1.00 equiv) was dissolved in trimethyl phosphate (TMP, 18.5 equiv) and cooled to 0 °C. Subsequently, phosphorus oxychloride ($POCl_3$, 1.20 equiv) was added dropwise, and the reaction solution was stirred at 0 °C. The progress of the reaction was monitored by thin-layer chromatography (TLC, acetonitrile 7:3 1.00 M TEAB buffer). Following this, an emulsion of tris(tetrabutylammonium) hydrogen pyrophosphate (0.850 equiv) and tributylamine (6.00 equiv) in dry acetonitrile (20.5 equiv) was added dropwise at 0 °C. The reaction solution was stirred for 20 min, followed by the addition of water. Subsequently, it was extracted with dichloromethane, and the aqueous phase was lyophilized. Purification of the crude product was performed by automated ion-exchange chromatography (DEAE-Sephadex® A25 column, 0 M to 1.00 M TEAB buffer in 60 min).

2.1.3 Linker Coupling (General Procedure 3)

A Schlenk flask containing the corresponding 5-iodonucleotide (1.00 equiv), tri(2-furyl)phosphine (12.0 mol%), tris(dibencylideneacetone)dipalladium(0) (5.40 mol%) and copper(I) iodide (15.0 mol%) was evaporated and backfilled with nitrogen thrice. The solids were then suspended in DMSO, and triethylamine (7.00 equiv) and pent-4-yn-1-amine (1.40 equiv) were added. The resulting mixture was stirred at 50 °C for 1 h. After full conversion of the starting material, as indicated by TLC ($MeCN/H_2O$ 7:3 v/v) for monophosphates or RP_{18}-HPLC for triphosphates, all volatiles were evaporated at 50 °C in an oil pump vacuum. The residue was taken up in $MeCN/H_2O$ (1:1 v/v) and filtered over a short RP_{18} silica plug. After lyophilization of the filtrate, the residue was subjected to RP_{18} flash chromatography (5 min 0.05 M TEAB buffer, then gradually to MeCN over 30 min). To remove excess buffer salts, the obtained solid was co-evaporated with methanol and again lyophilized from $MeCN/H_2O$ (1:1 v/v).

2.1.4 Carbamate Formation (General Procedure 4)
The corresponding 5-amino-1-pentyn-1-yl substituted nucleotide (1.00 equiv) was suspended in DMSO and triethylamine (3.50 equiv) was added. Following the addition of the corresponding NHS carbonate (1.15 equiv), the resulting mixture was stirred for 1 h at room temperature. After full conversion, as indicated by TLC (MeCN/H_2O 7:3 v/v), all volatiles were evaporated at 50 °C in an oil pump vacuum. The residue was subjected to RP_{18} flash chromatography (5 min 50.0 mM TEAB buffer, then gradually to MeCN over 30 min). To remove excess buffer salts, the obtained solid was co-evaporated with methanol and lyophilized from MeCN/H_2O (1:1 v/v).

2.1.5 Tri*PPP*ro Synthesis (General Procedure 5)
A solution of trifluoroacetic anhydride (5.00 equiv) and triethylamine (8.00 equiv) in MeCN (4 mL) was dropwise added to a 0 °C cold solution of bis[C9AB]-pyrophosphate (1.00 equiv) in MeCN (5 mL). After stirring for 15 min at room temperature, all volatiles were removed in an oil pump vacuum at room temperature, and the residue was co-evaporated with MeCN (2 mL). Following subsequent dissolution in DMF (5 mL), triethylamine (5.00 equiv) and 1-methylimidazole (2.50 equiv) were added, and the resulting solution was stirred for 15 min at room temperature before the corresponding monophosphate (0.4 – 0.5 equiv) was added in one portion. After full conversion of the starting material, as indicated by RP_{18}-HPLC, all volatiles were removed in an oil pump vacuum at room temperature, and the residue was preliminarily purified by RP_{18} flash chromatography (5 min H_2O, then gradually to MeCN over 30 min). All fractions containing the product were combined, lyophilized, and then subjected to a short ion-exchange column (Dowex NH_4^+). After lyophilization, a RP_{18} flash chromatography (5 min H_2O, then gradually to MeCN over 30 min) was carried out, and the process was repeated a second time to give the corresponding product.

2.2 Synthesis of Tri*PPP*ros

2.2.1 Preparation of ICMP (12)

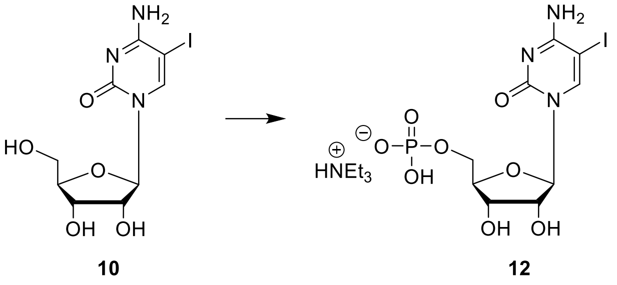

ICMP **12** was synthesized according to general procedure 1 with 5-Iodocytidine **10** (1.87 g, 5.06 mmol, 1.00 equiv) in a mixture of phosphoryl chloride (2.08 mL, 22.3 mmol, 4.40 equiv), pyridine (1.96 mL, 24.3 mmol, 4.80 equiv), and H_2O (183 µL, 10.1 mmol, 2.00 equiv) in MeCN (5 mL, 18.9 equiv) for 2.5 h.

The product (1.4 TEA, 1.96 g, 3.31 mmol, 65%) was obtained as a colorless solid.

^{1}H-NMR (600 MHz, D_2O): δ [ppm] = 8.23 (s, 1H), 5.93 (d, $^3J_{HH}$ = 4.3 Hz, 1H), 4.33 (dd, $^3J_{HH}$ = 5.3, 4.4 Hz, 1H), 4.30 (dd, $^3J_{HH}$ = 5.2 Hz, 1H), 4.26 – 4.24 (m, 2H), 4.13 (dd, $^3J_{HH}$ = 4.4, 2.8 Hz, 1H), 4.11 (dd, $^3J_{HH}$ = 4.4, 2.8 Hz, 1H), 4.06 (ddd, $^2J_{HH}$ = 11.8 Hz, $^3J_{HH}$ = 5.5 Hz, $^3J_{HP}$ = 3.5 Hz, 1H), 3.20 (q, $^3J_{HH}$ = 7.3 Hz, 8.6H), 1.28 (t, $^3J_{HH}$ = 7.3 Hz, 12.8H). **^{13}C-NMR** (151 MHz, D_2O): δ [ppm] = 164.6, 156.8, 147.5, 89.5, 83.2 (d, $^3J_{CP}$ = 8.8 Hz), 74.2, 69.4, 63.7 (d, $^2J_{CP}$ = 4.4 Hz), 58.1, 46.6, 8.2. **^{31}P-NMR** (243 MHz, D_2O): δ [ppm] = 1.40. **HRMS** (ESI$^+$): *m/z* cal: 449.9558 $[M+H]^+$, found: 449.9560.

2.2.2 Preparation of IUMP (13)

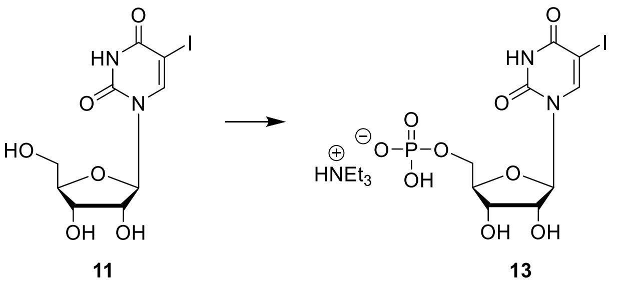

IUMP **13** was synthesized according to general procedure 1 with 5-Iodouridine **11** (1.00 g, 2.70 mmol, 1.00 equiv) in a mixture of phosphoryl chloride (1.11 mL, 11.9 mmol, 4.40 equiv), pyridine (1.05 mL, 13.0 mmol, 4.80 equiv), and H_2O (122 µL, 6.75 mmol, 2.50 equiv) in MeCN (2.7 mL, 19.1 equiv) for 2 h.

The product (1.4 TEA, 1.18 g, 2.01 mmol, 74%) was obtained as a colorless solid.

^{1}H-NMR (500 MHz, D_2O): δ [ppm] = 8.26 (s, 1H), 5.92 (d, $^3J_{HH}$ = 5.3 Hz, 1H), 4.37 (dd, $^3J_{HH}$ = 5.3 Hz, 1H), 4.31 (dd, $^3J_{HH}$ = 5.3, 4.2 Hz, 1H), 4.26 – 4.24 (m, 1H), 4.09 (ddd, $^2J_{HH}$ = 11.8 Hz, $^3J_{HP}$ = 4.4 Hz, $^3J_{HH}$ = 2.8 Hz, 1H), 4.04 (ddd, $^2J_{HH}$ = 11.8, $^3J_{HP}$ = 5.7 Hz, $^3J_{HH}$ = 3.5 Hz, 1H), 3.19 (q, $^3J_{HH}$ = 7.3 Hz, 8.5H), 1.27 (t, $^3J_{HH}$ = 7.3 Hz, 12.7H). **^{13}C-NMR** (126 MHz, D_2O): δ [ppm] = 152.0, 146.4, 146.3, 89.0, 84.1 (d,

$^{3}J_{CP}$ = 8.8 Hz), 74.2, 70.2, 68.9, 64.4 (d, $^{2}J_{CP}$ = 4.7 Hz), 47.0, 8.6. **^{31}P-NMR** (122 MHz, D_2O): δ [ppm] = 1.06. **HRMS** (ESI$^-$): *m/z* cal: 448.9252 [M-H]$^-$, found: 448.9254.

2.2.3 Preparation of 5-phthalimidopent-1-yn (33)

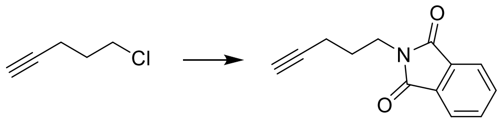

32 **33**

5-phthalimidopent-1-yn **33** was synthesized according to a literature procedure[3] with 5-chloro-1-pentyne **32** (5.30 mL, 50.0 mmol, 1.00 equiv), phthalimide (8.83 g, 60.0 mmol, 1.20 equiv), potassium carbonate (6.91 g, 50.0 mmol, 1.00 equiv) and potassium iodide (100 mg, 602 µmol, 1.20 mol%) in DMF (50 mL).

The product (10.6 g, 49.5 mmol, 99%) was obtained as a colorless solid. The analytical data agreed with those reported in literature.[3]

2.2.4 Preparation of pent-4-yn-1-amine (34)

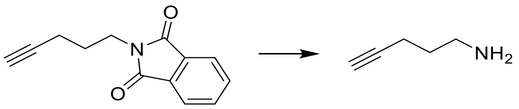

33 **34**

Pent-4-yn-1-amine **34** was synthesized according to a literature procedure[3] with 5-phthalimidopent-1-yn **33** (7.40 g, 34.7 mmol, 1.00 equiv) and hydrazine hydrate (21.6 mL, 347 mmol, 10.0 equiv) in ethanol (70 mL). (Caution: the product is volatile, no vacuum below 150 mbar should be applied at 40 °C.)

The product (2.65 g, 31.9 mmol, 92 %) was obtained as a light-yellow oil. The analytical data agreed with those reported in literature.[3]

2.2.5 Preparation of AP-CMP (14)

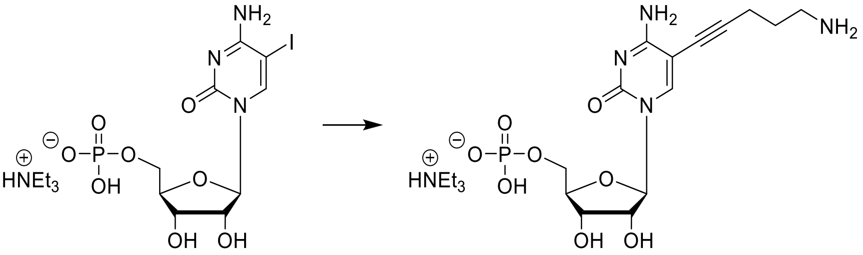

12 **14**

AP-CMP **14** was synthesized according to general procedure 3 with ICMP **12** (300 mg, 508 µmol, 1.00 equiv), tri(2-furyl)phosphine (14.1 mg, 60.9 µmol, 12.0 mol%), tris(dibencylideneacetone)dipalladium(0) (25.1 mg, 27.4 µmol, 5.40 mol%), copper(I) iodide (14.5 mg, 76.2 µmol, 15.0 mol%), triethylamine (495 µL, 355 µmol, 7.00 equiv), and pent-4-yn-1-amine (59.1 mg, 711 µmol, 1.40 equiv) in DMSO (3 mL).

The product (1.2 TEA, 243 mg, 462 µmol, 91%) was obtained as a light-yellow solid.

^{1}H-NMR (400 MHz, D_2O): δ [ppm] = 8.25 (s, 1H), 6.01 (d, $^3J_{HH}$ = 3.7 Hz, 1H), 4.37-4.33 (m, 2H), 4.28-4.26 (m, 1H), 4.10 (ddd, $^2J_{HH}$ = 12.2 Hz, $^3J_{HH}$ = 2.8 Hz, $^3J_{HP}$ = 2.8 Hz, 1H), 4.04 (ddd, $^2J_{HH}$ = 11.9 Hz, $^3J_{HH}$ = 5.4 Hz, $^3J_{HP}$ = 2.9 Hz, 1H), 3.22 (q, $^3J_{HH}$ = 7.3 Hz, 7.2H), 3.21 (t, $^3J_{HH}$ = 7.2 Hz, 2H), 2.64 (t, $^3J_{HH}$ = 6.5 Hz, 2H), 1.99 (quin, $^3J_{HH}$ = 6.8 Hz, 2H), 1.30 (t, $^3J_{HH}$ = 7.3 Hz, 10.8H). **^{13}C-NMR** (101 MHz, D_2O): δ [ppm] = 165.1, 156.2, 144.2, 95.4, 93.0, 89.4, 83.4 (d, $^3J_{CP}$ = 8.7 Hz), 74.6, 72.1, 69.3, 62.9 (d, $^2J_{CP}$ = 4.5 Hz), 46.7, 38.5, 25.2, 16.1, 8.2. **^{31}P-NMR** (162 MHz, D_2O): δ [ppm] = 3.04. **HRMS** (ESI$^+$): *m/z* cal: 405.1170 $[M+H]^+$, found: 405.1159.

2.2.6 Preparation of AP-UMP (15)

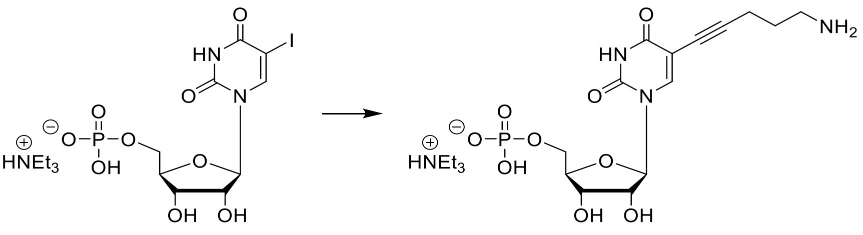

13 **15**

AP-UMP **15** was synthesized according to general procedure 3 with IUMP **13** (480 mg, 817 µmol, 1.00 equiv), tri(2-furyl)phosphine (22.8 mg, 98.1 µmol, 12.0 mol%), tris(dibencylideneacetone)dipalladium(0) (40.4 mg, 44.1 µmol, 5.40 mol%), copper(I) iodide (23.3 mg, 123 µmol, 15.0 mol%), triethylamine (797 µL, 5.72 mmol, 7.00 equiv), and pent-4-yn-1-amine (102 mg, 1.22 mmol, 1.50 equiv) in DMSO (4 mL).

The product (0.6 TEA, 322 mg, 686 µmol, 84%) was obtained as a light-yellow solid.

^{1}H-NMR (600 MHz, D_2O): δ [ppm] = 8.19 (s, 1H), 5.99 (d, $^3J_{HH}$ = 5.0 Hz, 1H), 4.38 (dd, $^3J_{HH}$ = 5.1 Hz, 1H), 4.34 (dd, $^3J_{HH}$ = 5.1, 4.3 Hz, 1H), 4.29 – 4.27 (m, 1H), 4.10 (ddd, $^2J_{HH}$ = 11.9 Hz, $^3J_{HH}$ = 2.8 Hz, $^3J_{HP}$ = 2.8 Hz, 1H), 4.05 (ddd, $^2J_{HH}$ = 11.9 Hz, $^3J_{HH}$ = 5.4 Hz, $^3J_{HP}$ = 2.8 Hz, 1H), 3.21 (q, $^3J_{HH}$ = 7.3 Hz, 3.8H), 3.18 (t, $^3J_{HH}$ = 7.3 Hz, 2H), 2.59 (t, $^3J_{HH}$ = 6.6 Hz, 2H), 1.97 (quin, $^3J_{HH}$ = 6.9 Hz, 2H), 1.28 (t, $^3J_{HH}$ = 7.3 Hz, 5.5H). **^{13}C-NMR** (151 MHz, D_2O): δ [ppm] = 164.8, 150.8, 143.7, 100.0, 93.9, 88.5, 83.9 (d, $^3J_{CP}$ = 9.4 Hz), 74.2, 72.4, 69.7, 63.5 (d, $^2J_{CP}$ = 4.4 Hz), 46.6, 38.6, 25.2, 16.0, 8.2. **^{31}P-NMR** (243 MHz, D_2O): δ [ppm] = 4.64. **HRMS** (ESI$^+$): *m/z* cal: 406.1010 $[M+H]^+$, found: 406.1010.

2.2.7 Preparation of 2TCO*a*-CMP (16)

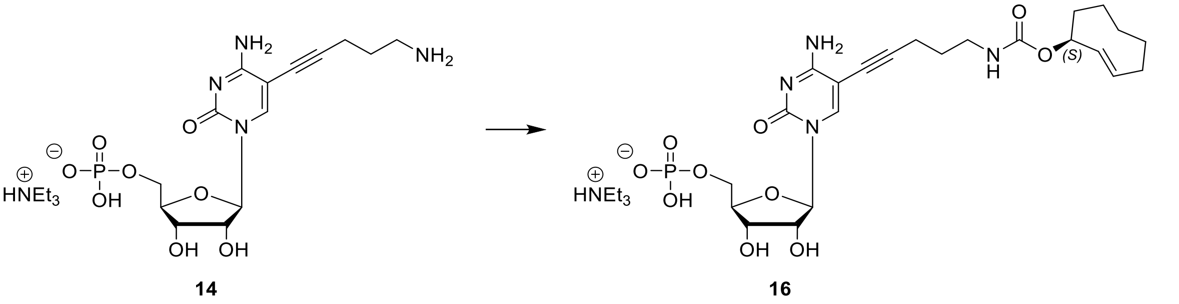

2TCO*a*-CMP **16** was synthesized according to general procedure 4 with AP-CMP **14** (100 mg, 194 µmol, 1.00 equiv), triethylamine (94.8 µL, 680 µmol, 3.50 equiv), and 2TCO*a*-NHS (59.7 mg, 223 µmol, 1.15 equiv) in DMSO (1 mL).

The product (1.7 TEA, 119 mg, 163 µmol, 84%) was obtained as a colorless solid.

^{1}H-NMR (600 MHz, MeOD): δ [ppm] = 8.09 (s, 1H), 5.59 (d, $^3J_{HH}$ = 4.6 Hz, 1H), 5.86 – 5.81 (ddd, $^3J_{HH}$ = 16.2, 11.3, 3.7 Hz, 1H), 5.54 (dd, $^3J_{HH}$ = 16.4, 2.4 Hz, 1H), 5.24 (bs, 1H), 4.21 (dd, $^3J_{HH}$ = 4.8 Hz, 1H), 4.17 (dd, $^3J_{HH}$ = 4.9 Hz, 1H), 4.14 – 4.06 (m, 3H), 3.28 – 3.26 (m, 2H), 3.02 (q, $^3J_{HH}$ = 7.2 Hz, 10.4H), 2.48 (t, $^3J_{HH}$ = 6.8 Hz, 2H), 2.45 – 2.43 (m, 1H), 2.04 – 1.95 (m, 3H), 1.88 – 1.83 (m, 1H), 1.77 (quin, $^3J_{HH}$ = 6.7 Hz, 2H), 1.74 – 1.68 (m, 1H), 1.65 – 1.60 (m, 1H), 1.53 – 1.46 (m, 1H), 1.24 (t, $^3J_{HH}$ = 7.3 Hz, 15.3H), 1.18 – 1.13 (m, 1H), 0.89 – 0.83 (m, 1H). **^{13}C-NMR** (151 MHz, MeOD): δ [ppm] = 166.6, 158.5, 157.3, 145.0, 133.0, 132.6, 97.0, 94.1, 91.1, 85.0 (d, $^3J_{CP}$ = 8.8 Hz), 76.1, 75.0, 72.3, 71.3, 65.3 (d, $^2J_{CP}$ = 5.0 Hz), 47.4, 41.7, 40.6, 37.0, 36.8, 30.1, 29.7, 25.2, 17.6, 9.6. **^{31}P-NMR** (243 MHz, MeOD): δ [ppm] = 1.49. **HRMS** (ESI$^-$): *m/z* cal: 555.1862 $[M-H]^-$, found: 555.1866.

2.2.8 Preparation of 2TCO*a*-UMP (18)

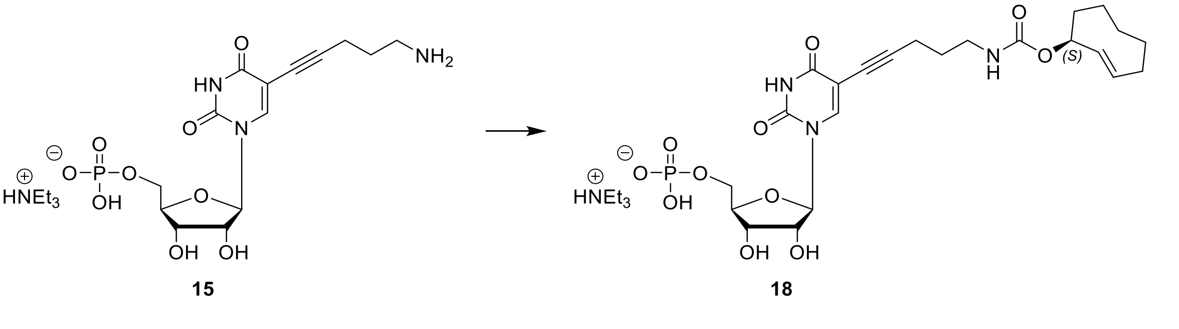

2TCO*a*-UMP **18** was synthesized according to general procedure 4 with AP-UMP **15** (80.0 mg, 171 µmol, 1.00 equiv), triethylamine (83.2 µL, 597 µmol, 3.50 equiv), and 2TCO*a*-NHS (52.4 mg, 196 µmol, 1.15 equiv) in DMSO (1 mL).

The product (1.9 TEA, 113 mg, 150 µmol, 88%) was obtained as a colorless solid.

^{1}H-NMR (400 MHz, MeOD): δ [ppm] = 8.03 (s, 1H), 5.95 (d, $^3J_{HH}$ = 5.7 Hz, 1H), 5.88 – 5.80 (m, 1H), 5.53 (dd, $^3J_{HH}$ = 16.8, 2.4 Hz, 1H), 5.22 (bs, 1H), 4.27 (dd, $^3J_{HH}$ = 5.5 Hz, 1H), 4.24 (dd, $^3J_{HH}$ = 5.3, 3.0 Hz, 1H), 4.12 – 4.10 (m, 1H), 4.07 – 4.05 (m, 2H), 3.25 – 3.22 (m, 2H), 3.01 (q, $^3J_{HH}$ = 7.3 Hz, 11.2H), 2.45 – 2.42 (m, 3H), 2.06 – 1.93 (m, 3H), 1.88 – 1.82 (m, 1H), 1.78 – 1.73 (m, 2H), 1.71 – 1.67 (m, 1H), 1.65 – 1.59 (m, 1H), 1.55 – 1.47 (m, 1H), 1.23 (t, $^3J_{HH}$ = 7.3 Hz, 17.3H), 1.20 – 1.11 (m, 1H), 0.90 – 0.82 (m, 1H). **^{13}C-NMR** (151 MHz, MeOD): δ [ppm] = 164.8, 158.5, 151.7, 144.2, 133.0, 132.6, 101.7, 95.0, 89.7, 85.7 (d, $^3J_{CP}$ = 7.7 Hz), 75.2, 75.0, 72.9, 71.8, 65.7 (d, $^3J_{CP}$ = 5.0 Hz), 47.5, 41.7, 40.9, 37.0, 36.8, 30.1, 29.5, 25.2, 17.8, 9.5. **^{31}P-NMR** (243 MHz, MeOD): δ [ppm] = 2.14. **HRMS** (ESI$^-$): *m/z* cal: 556.1702 [M-H]$^-$, found: 556.1667.

2.2.9 Preparation of BCN-CMP (17)

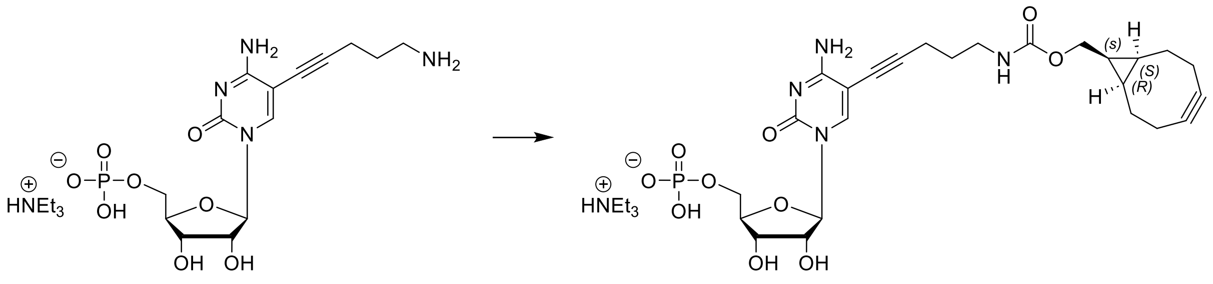

14 **17**

BCN-CMP **17** was synthesized according to general procedure 4 with AP-CMP **14** (100 mg, 205 µmol, 1.00 equiv), triethylamine (100 µL, 717 µmol, 3.50 equiv), and BCN-NHS (68.6 mg, 236 µmol, 1.15 equiv) in DMSO (1 mL).

The product (1.4 TEA, 143 mg, 196 µmol, 96%) was obtained as a colorless solid.

^{1}H-NMR (500 MHz, MeOD): δ [ppm] = 8.12 (s, 1H), 5.96 (d, $^3J_{HH}$ = 4.7 Hz, 1H), 4.21 (dd, $^3J_{HH}$ = 4.8 Hz, 1H), 4.17 (dd, $^3J_{HH}$ = 4.9 Hz, 1H), 4.14 – 4.06 (m, 5H), 3.27 (t, $^3J_{HH}$ = 6.6 Hz, 2H), 3.08 (q, $^3J_{HH}$ = 7.4 Hz, 8.7H), 2.48 (t, $^3J_{HH}$ = 6.8 Hz, 2H), 2.28 – 2.20 (m, 4H), 2.18 – 2.14 (m, 2H), 1.76 (quin, $^3J_{HH}$ = 6.8 Hz, 2H), 1.64 – 1.56 (m, 2H), 1.37 (quin, $^3J_{HH}$ = 8.0 Hz, 1H), 1.26 (t, $^3J_{HH}$ = 7.3 Hz, 13H), 0.96 – 0.89 (m, 2H). **^{13}C-NMR** (126 MHz, MeOD): δ [ppm] = 166.6, 159.4, 157.3, 145.0, 99.5, 97.0, 94.0, 91.1, 85.0 (d, $^3J_{CP}$ = 8.6 Hz), 76.2, 72.3, 71.2, 65.3 (d, $^2J_{CP}$ = 4.7 Hz), 63.6, 47.5, 40.6, 30.1, 29.6, 22.0, 21.4, 19.0, 17.6, 9.5. **^{31}P-NMR** (202 MHz, MeOD): δ [ppm] = 1.29. **HRMS** (ESI$^-$): *m/z* cal: 579.1862 [M-H]$^-$, found: 579.1873

2.2.10 Preparation of BCN-UMP (19)

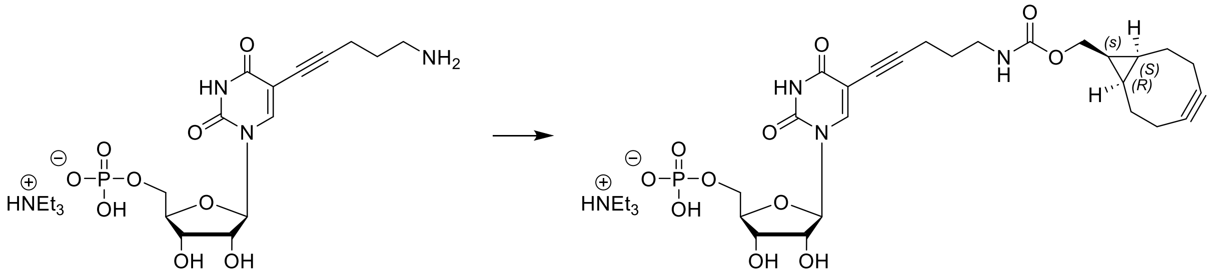

15 19

BCN-UMP **19** was synthesized according to general procedure 4 with AP-UMP **15** (100 mg, 181 µmol, 1.00 equiv), triethylamine (88.5 µL, 635 µmol, 3.50 equiv), and BCN-NHS (60.8 mg, 209 µmol, 1.15 equiv) in DMSO (1 mL).

The product (2.0 TEA, 119 mg, 152 µmol, 84%) was obtained as a colorless solid.

^{1}H-NMR (600 MHz, MeOD): *δ* [ppm] = 8.03 (s, 1H), 5.96 (d, $^{3}J_{HH}$ = 6.0 Hz, 1H), 4.27 (dd, $^{3}J_{HH}$ = 5.7 Hz, 1H), 4.24 (dd, $^{3}J_{HH}$ = 5.3, 3.2 Hz, 1H), 4.13 – 4.10 (m, 3H), 4.07 – 4.06 (m, 2H), 3.23 (dd, $^{3}J_{HH}$ = 6.9, 6.0 Hz, 2H), 3.04 (q, $^{3}J_{HH}$ = 7.3 Hz, 12H), 2.43 (t, $^{3}J_{HH}$ = 7.0 Hz, 2H), 2.29 – 2.16 (m, 6H), 1.76 (quin, $^{3}J_{HH}$ = 6.7 Hz, 2H), 1.64 – 1.58 (m, 2H), 1.37 (quin, $^{3}J_{HH}$ = 8.3 Hz, 1H), 1.25 (t, $^{3}J_{HH}$ = 7.3 Hz, 18H), 0.95 – 0.90 (m, 2H). **^{13}C-NMR** (151 MHz, MeOD): *δ* [ppm] = 164.7, 159.4, 151.7, 144.2, 101.7, 99.5, 94.9, 89.7, 85.7 (d, $^{3}J_{CP}$ = 8.3 Hz), 75.2, 72.8, 71.8, 65.7 (d, $^{2}J_{CP}$ = 4.8 Hz), 63.5, 47.5, 40.9, 29.9, 29.5, 22.2, 21.3, 19.0, 17.7, 9.6. **^{31}P-NMR** (243 MHz, MeOD): *δ* [ppm] = 1.29. **HRMS** (ESI^{-}): *m/z* cal: 580.1702 [M-H]$^{-}$, found: 580.1705.

2.2.11 Preparation of Bis[C9AB]-pyrophosphate (36)

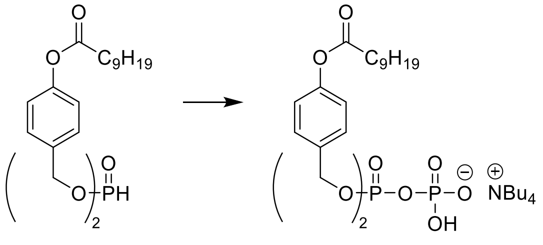

35 36

Bis[C9AB]-pyrophosphate **36** was synthesized according to a literature procedure.[4] Briefly, to a suspension of an appropriate amount of bis[C9AB]-*H*-phosphonate[3] **35** (1.00 equiv) in MeCN (10 mL) was added NCS (2.50 equiv), and the resulting mixture was stirred at 50 °C for 3 h. After complete consumption of the starting material, as indicated by ^{1}H-NMR, the obtained solution was dropwise added to a solution of tetrabutylammonium dihydrogen phosphate (0.4 M in MeCN, 3.00 equiv) at rt, and the mixture was stirred for an additional hour. Subsequently, all volatiles were removed under an oil pump vacuum at room temperature. The residue was then dissolved in DCM (20 mL) and washed

sequentially with an ammonium acetate solution (1.00 M in H_2O, 15 mL) and H_2O (15 mL), using a centrifuge for faster phase separation. The organic phase was dried over sodium sulfate and evaporated in an oil pump vacuum at room temperature.

The product (usually >90%) was obtained as a colorless oil and used immediately in the next step without further purification. (Caution: the pyrophosphate is not stable upon prolonged storage. Experiential, it can be stored under a nitrogen atmosphere either overnight at -20 °C or over three days at -80 °C.)

2.2.12 Preparation of Bis[C9AB]-2TCO*a*-CTP (4)

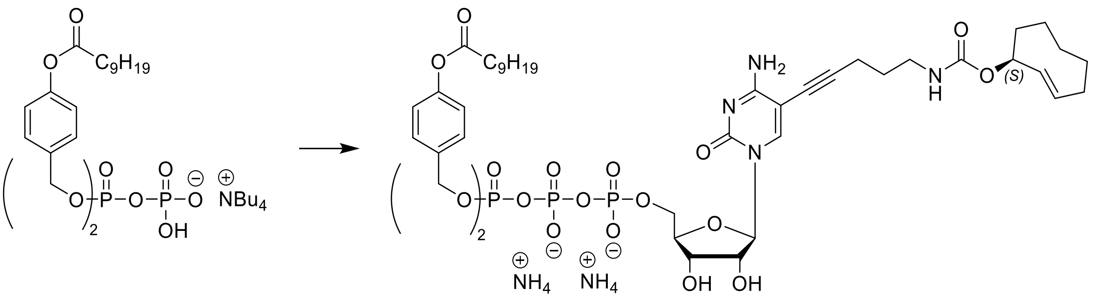

36 **4**

Bis[C9AB]-2TCO*a*-CTP **4** was synthesized according to general procedure 5. Bis[C9AB]-pyrophosphate **36** (299 mg, 318 µmol, 1.00 equiv) was stepwise activated with trifluoroacetic anhydride (221 µL, 1.59 mmol, 5.0 equiv) and triethylamine (354 µL, 2.54 mmol, 8.00 equiv) as well as 1-methylimidazole (63.3 µL, 795 µmol, 2.50 equiv), and triethylamine (221 µL, 1.59 mmol, 5.00 equiv), and subsequently reacted with 2TCO*a*-CMP **16** (100 mg, 140 µmol, 0.440 equiv) for 2 h.

The product (59.4 mg, 44.7 µmol, 32%) was obtained as a colorless cotton.

^{1}H-NMR (600 MHz, MeOD): δ [ppm] = 8.15 (s, 1H), 7.40 – 7.38 (m, 4H), 7.04 – 7.03 (m, 4H), 5.86 (d, $^3J_{HH}$ = 4.2 Hz, 1H), 5.85 – 5.80 (m, 1H), 5.52 (dd, $^3J_{HH}$ = 16.5, 2.6 Hz, 1H), 5.22 (bs, 1H), 5.18 – 5.15 (m, 4H), 4.32 (dd, $^3J_{HH}$ = 5.3 Hz, 1H), 4.31 – 4.29 (m, 2H), 4.23 (dd, $^3J_{HH}$ = 4.7 Hz, 1H), 4.17 – 4.15 (m, 1H), 3.24 – 3.21 (m, 2H), 2.57 (t, $^3J_{HH}$ = 7.4 Hz, 4H), 2.45 (t, $^3J_{HH}$ = 6.9 Hz, 2H), 2.43 – 2.41 (m, 1H), 2.02 – 1.93 (m, 3H), 1.86 – 1.81 (m, 1H), 1.76 – 1.66 (m, 5H), 1.72 (quin, $^3J_{HH}$ = 7.4 Hz, 2H), 1.63 – 1.58 (m, 1H), 1.50 – 1.27 (m, 25H), 1.16 – 1.11 (m, 1H), 0.92 – 0.89 (m, 6H), 0.87 – 0.81 (m, 1H). **^{13}C-NMR** (151 MHz, MeOD): δ [ppm] = 173.7, 164.1, 158.5, 152.3, 146.1, 135.0 (d, $^3J_{CP}$ = 7.7 Hz), 133.0, 132.6, 130.5 (d, $^4J_{CP}$ = 2.8 Hz), 122.8, 98.4, 93.9, 91.8, 84.9 (d, $^3J_{CP}$ = 9.4 Hz), 76.0, 75.0, 71.0, 70.7, 70.5 (d, $^2J_{CP}$ = 4.5 Hz), 66.1 (d, $^2J_{CP}$ = 4.1 Hz), 41.7, 40.6, 37.0, 36.8, 35.0, 33.1, 30.6, 30.5, 30.4, 30.2, 30.1, 29.5, 26.0, 25.2, 23.7, 17.7, 14.5. **^{31}P-NMR** (243 MHz, MeOD): δ [ppm] = -11.60 (d, $^2J_{PP}$ = 19.4 Hz), -13.18 (d, $^2J_{PP}$ = 17.2 Hz), -23.62 (dd, $^2J_{PP}$ = 18.0 Hz). **HRMS** (ESI$^-$): *m/z* cal: 617.2334 [M-2H]$^-$, found: 617.2120.

2.2.13 Preparation of Bis[C9AB]-2TCO*a*-UTP (2)

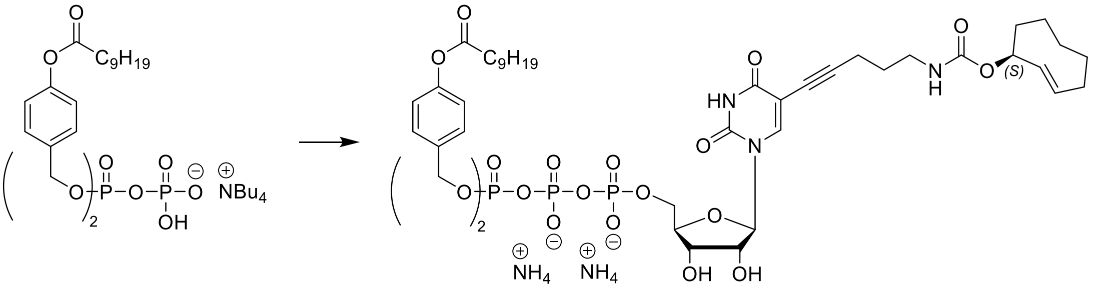

36 2

Bis[C9AB]-2TCO*a*-UTP **2** was synthesized according to general procedure 5. Bis[C9AB]-pyrophosphate **36** (199 mg, 212 µmol, 1.00 equiv) was stepwise activated with trifluoroacetic anhydride (147 µL, 1.06 mmol, 5.00 equiv) and triethylamine (236 µL, 1.69 mmol, 8.00 equiv) as well as 1-methylimidazole (42.2 µL, 530 µmol, 2.50 equiv), and triethylamine (148 µL, 1.06 mmol, 5.00 equiv), and subsequently reacted with 2TCO*a*-UMP **18** (65.0 mg, 88.1 µmol, 0.420 equiv) for 2 h.

The product (42.4 mg, 33.3 µmol, 38%) was obtained as a colorless cotton.

^{1}H-NMR (600 MHz, MeOD): *δ* [ppm] = 7.95 (s, 1H), 7.41 – 7.39 (m, 4H), 7.05 – 7.04 (m, 4H), 5.91 (d, $^{3}J_{HH}$ = 5.7 Hz, 1H), 5.85 – 5.80 (m, 1H), 5.52 (d, $^{3}J_{HH}$ = 16.3, 2.3 Hz, 1H), 5.21 (bs, 1H), 5.18 – 5.15 (m, 4H), 4.30 (dd, $^{3}J_{HH}$ = 4.7 Hz, 1H), 4.27 – 4.22 (m, 3H), 4.13 – 4.11 (m, 1H), 3.20 (t, $^{3}J_{HH}$ = 6.6 Hz, 2H), 2.57 (t, $^{3}J_{HH}$ = 7.4 Hz, 4H), 2.44 – 2.37 (m, 3H), 2.03 – 1.93 (m, 3H), 1.87 – 1.82 (m, 1H), 1.75 – 1.67 (m, 7H), 1.63 – 1.58 (m, 1H), 1.50 – 1.28 (m, 25H), 1.16 – 1.11 (m, 1H), 0.91 (m, 6H), 0.87 – 0.82 (m, 1H). **^{13}C-NMR** (151 MHz, MeOD): *δ* [ppm] = 173.8, 164.6, 158.4, 152.3, 151.6, 144.1, 134.9 (d, $^{3}J_{CP}$ = 7.7 Hz), 132.9, 132.6, 130.5 (d, $^{4}J_{CP}$ = 3.3 Hz), 122.8, 101.7, 95.1, 90.0, 85.0 (d, $^{3}J_{CP}$ = 9.5 Hz), 75.0, 75.0, 72.8, 71.3, 70.5 (d, $^{2}J_{CP}$ = 5.5 Hz), 66.8 (d, $^{2}J_{CP}$ = 5.5 Hz), 41.7, 40.9, 37.0, 36.8, 35.0, 33.1, 30.6, 30.4, 30.4, 30.2, 30.1, 29.5, 26.0, 25.2, 23.7, 17.8, 14.4. **^{31}P-NMR** (243 MHz, MeOD): *δ* [ppm] = -11.67 (d, $^{2}J_{PP}$ = 18.7 Hz), -13.24 (d, $^{2}J_{PP}$ = 17.2 Hz), -23.90 (dd, $^{2}J_{PP}$ = 17.2 Hz). **HRMS** (ESI^{-}): *m/z* cal: 617.7254 $[M-2H]^{2-}$, found: 617.6962.

2.2.14 Preparation of Bis[C9AB]-BCN-CTP (3)

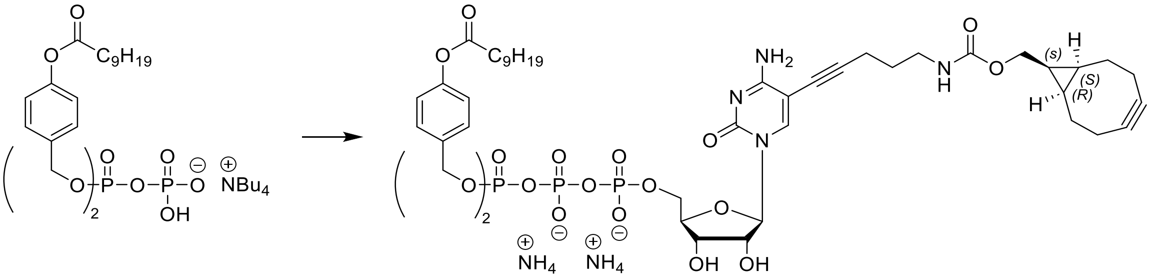

36 3

Bis[C9AB]-BCN-CTP **3** was synthesized according to general procedure 5. Bis[C9AB]-pyrophosphate **36** (295 mg, 314 µmol, 1.00 equiv) was stepwise activated with trifluoroacetic anhydride (218 µL, 1.57 mmol, 5.00 equiv), and triethylamine (350 µL, 2.51 mmol, 8.00 equiv) as well as 1-methylimidazole (62.5 µL, 784 µmol, 2.50 equiv), and triethylamine (219 µL, 1.57 mmol, 5.00 equiv), and subsequently reacted with BCN-CMP **17** (100 mg, 138 µmol, 0.440 equiv) for 2 h.

The product (43.5 mg, 33.6 µmol, 24%) was obtained as a colorless cotton.

^{1}H-NMR (600 MHz, MeOD): δ [ppm] = 8.22 (s, 1H), 7.40 – 7.38 (m, 4H), 7.05 – 7.03 (m, 4H), 5.85 (d, $^{3}J_{HH}$ = 4.1 Hz, 1H), 5.18 – 5.16 (m, 4H), 4.34 (dd, $^{3}J_{HH}$ = 5.2 Hz, 1H), 4.33 – 4.28 (m, 2H), 4.17 – 4.16 (m, 1H), 4.10 (d, $^{3}J_{HH}$ = 8.1 Hz, 2H), 3.23 (t, $^{3}J_{HH}$ = 6.6 Hz, 2H), 2.57 (t, $^{3}J_{HH}$ = 7.4 Hz, 4H), 2.46 (t, $^{3}J_{HH}$ = 6.8 Hz, 2H), 2.24 – 2.18 (m, 4H), 2.15 – 2.12 (m, 2H), 1.77 – 1.70 (m, 6H), 1.59 – 1.53 (m, 2H), 1.45 – 1.27 (m, 32H), 0.92 – 0.89 (m, 8H). **^{13}C-NMR** (MHz, MeOD): δ [ppm] = 173.7, 163.1, 159.7, 159.4, 152.3, 146.6, 135.0 (d, $^{3}J_{CP}$ = 6.9 Hz), 130.5 (d, $^{4}J_{CP}$ = 2.2 Hz), 122.8, 99.6, 99.5, 93.7, 91.9, 85.1 (d, $^{3}J_{CP}$ = 9.1 Hz), 76.0, 70.7, 70.5, 66.0 (d, $^{2}J_{CP}$ = 6.4 Hz), 63.6, 40.7, 35.1, 33.1, 30.6, 30.5, 30.4, 30.4, 30.2, 30.2, 29.4, 26.0, 23.7, 22.0, 21.4, 19.0, 17.7, 14.5. **^{31}P-NMR** (243 MHz, MeOD): δ [ppm] = -11.72 (d, $^{2}J_{PP}$ = 19.4 Hz), -13.19 (d, $^{2}J_{PP}$ = 17.2 Hz), -23.68 (dd, $^{2}J_{PP}$ = 18.3 Hz). **HRMS** (ESI^{+}): *m/z* cal: 1261.4886 [M+H]$^{+}$, found: 1261.4888.

2.2.15 Preparation of Bis[C9AB]-BCN-UTP (1)

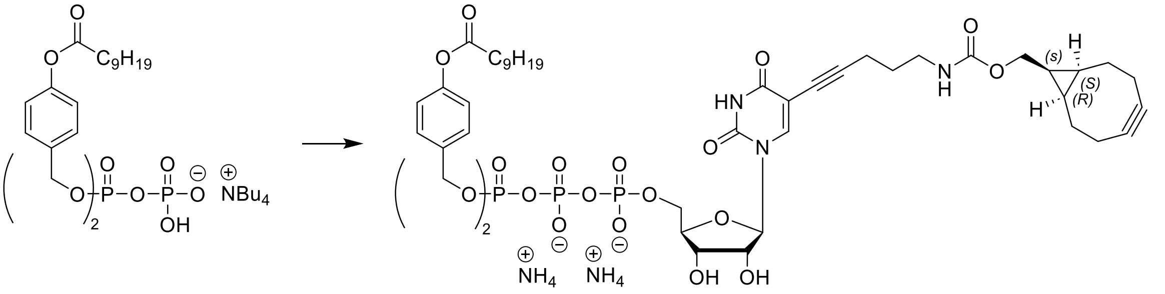

36 **1**

Bis[C9AB]-BCN-UTP **1** was synthesized according to general procedure 5. Bis[C9AB]-pyrophosphate **36** (262 mg, 279 µmol, 1.00 equiv) was stepwise activated with trifluoroacetic anhydride (194 µL, 1.39 mmol, 5.00 equiv), and triethylamine (311 µL, 2.23 mmol, 8.00 equiv) as well as 1-methylimidazole (56.0 µL, 697 µmol, 2.50 equiv), and triethylamine (194 µL, 1.39 mmol, 5.00 equiv), and subsequently reacted with BCN-UMP **19** (100 mg, 128 µmol, 0.460 equiv) for 2 h.

The product (52.5 mg, 40.5 µmol, 32%) was obtained as a colorless cotton.

^{1}H-NMR (500 MHz, MeOD): δ [ppm] = 7.97 (s, 1H), 7.42 – 7.39 (m, 4H), 7.06 – 7.03 (m, 4H), 5.92 (d, $^{3}J_{HH}$ = 5.8 Hz, 1H), 5.19 – 5.16 (m, 4H), 4.32 (dd, $^{3}J_{HH}$ = 5.4, 3.8 Hz, 1H), 4.29 – 4.21 (m, 3H), 4.14 – 4.08 (m, 3H), 3.23 – 3.19 (m, 2H), 2.57 (t, $^{3}J_{HH}$ = 7.4 Hz, 6H), 2.39 (t, $^{3}J_{HH}$ = 7.0 Hz, 2H), 2.28 – 2.11 (m, 7H), 1.76 – 1.70 (m, 8H), 1.66 – 1.54 (m, 3H), 1.39 – 1.27 (m, 32H), 0.92 – 0.89 (m, 8H). **^{13}C-NMR** (126 MHz, D_2O): δ [ppm] = 173.8, 164.6, 152.3, 151.6, 144.2, 135.1 (d, $^{3}J_{CP}$ = 7.3 Hz), 135.0, 130.5 (d, $^{4}J_{CP}$ = 3.0 Hz), 130.5, 122.8, 101.7, 99.5, 89.8, 85.2 (d, $^{3}J_{CP}$ = 9.0 Hz), 75.0, 71.4, 70.4, 70.4, 66.6 (d, $^{2}J_{CP}$ = 5.8 Hz), 63.5, 40.9, 35.1, 33.1, 30.6, 30.5, 30.4, 30.2, 30.2, 29.5, 26.0, 23.8, 22.0, 21.4, 19.0, 17.8, 14.5, 9.2. **^{31}P-NMR**

(202 MHz, D_2O): δ [ppm] = -11.66 (d, $^2J_{PP}$ = 18.7 Hz), -13.29 (d, $^2J_{PP}$ = 17.4 Hz), -23.82 (t, $^2J_{PP}$ = 18.7 Hz).
HRMS ($ESI^{+/-}$): *m/z* cal: $[M-H]^-$ 1260.4581, found: 1260.45788.

2.3 Synthesis of Triphosphates

2.3.1 Preparation of ICTP (20)

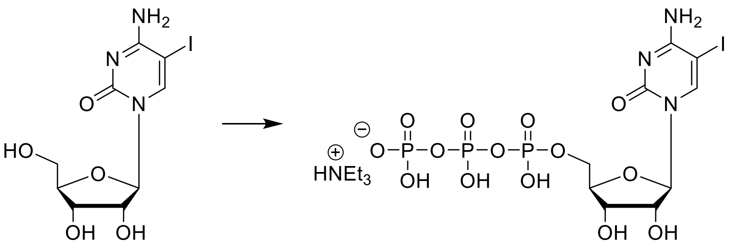

10 **20**

ICTP **20** was synthesized according to general procedure 2. 5-Iodocytidine **10** (500 mg, 1.36 mmol, 1.00 equiv) was dissolved in trimethyl phosphate (3 mL). Phosphorus oxychloride (152 µL, 249 mg, 1.63 mmol, 1.20 equiv) was added dropwise. Tris(tetrabutylammonium) hydrogen pyrophosphate (1.04 g, 1.20 mmol, 0.85 equiv), and 1.93 mL tributylamine (1.90 g, 8.10 mmol, 6.00 equiv) in MeCN (1.5 mL) were added. The obtained product was purified by automated reversed-phase (RP18) column chromatography (100% 50.0 mM TEAB buffer to 100% acetonitrile in 30 min).

The product (4.0 TEA, 319 mg, 321 µmol, 28%) was obtained as a colorless solid.

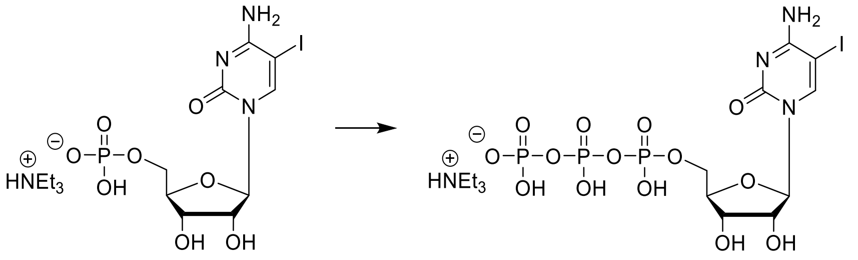

12 **20**

Alternatively, ICTP **20** can be synthesized from the corresponding monophosphate **12** according to a slightly modified procedure previously published by MOHAMADY.[5]

Triethylamine (283 µL, 2.03 mmol, 12.0 equiv) and TFAA (235 µL, 1.69 mmol, 10.0 equiv) were sequentially added dropwise to a suspension of ICMP **12** (100 mg, 169 µmol, 1.00 equiv) in MeCN (5 mL). The resulting mixture was stirred for 10 min at room temperature before all volatiles were removed in an oil pump vacuum at rt. The residue was taken up in MeCN (5 mL), and triethylamine (236 µL, 1.69 mmol, 10.0 equiv), followed by sequential addition of 1-methylimidazole (67.0 µL, 846 µmol, 5.00 equiv). After stirring for 10 min at rt, a solution of tris(tetrabutylammonium) hydrogen pyrophosphate (458 mg, 508 µmol, 3.00 equiv) in MeCN (5 mL) was added dropwise via a syringe pump (0.2 mL/min). After complete addition, the resulting mixture was stirred for 1 h at rt, before all volatiles were removed in an oil pump vacuum at room temperature. The residue was dissolved in H_2O, washed thrice with DCM, and finally purified by automated ion-exchange chromatography (DEAE-Sephadex® A25 column, 5 min H_2O, then gradually to 1.00 M TEAB buffer over 30 min) and RP_{18} flash chromatography (5 min 0.05 TEAB buffer, then gradually to MeCN over 30 min).

The product (4.0 TEA, 141 mg, 82%) was obtained as a yellow solid.

^{1}H-NMR (400 MHz, D_2O): δ [ppm] = 8.24 (s, 1H), 5.91 (d, $^3J_{HH}$ = 4.6 Hz, 1H), 4.44 (t, $^3J_{HH}$ = 5.1 Hz, 1H), 4.32 (t, $^3J_{HH}$ = 4.9 Hz, 1H), 4.29 – 4.20 (m, 3H), 3.15 (q, $^3J_{HH}$ = 7.4 Hz, 25H), 1.24 (t, $^3J_{HH}$ = 7.3 Hz, 36H). **^{13}C-NMR** (101 MHz, D_2O): δ [ppm] = 164.7, 156.9, 147.5, 89.2, 83.0 (d, $^3J_{CP}$ = 9.2 Hz), 74.3, 69.0, 64.6 (d, $^2J_{CP}$ = 5.8 Hz), 58.2, 46.6, 8.2. **^{31}P-NMR** (162 MHz, D_2O): δ [ppm] = -8.06 (bs), -11.68 (d, $^2J_{PP}$ = 20.2 Hz), -22.86 (dd, $^2J_{PP}$ = 20.5 Hz). **MS** (ESI$^-$): *m/z* cal: 607.8739 [M-H]$^-$, found: 607.8745.

2.3.2 Preparation of IUTP (21)

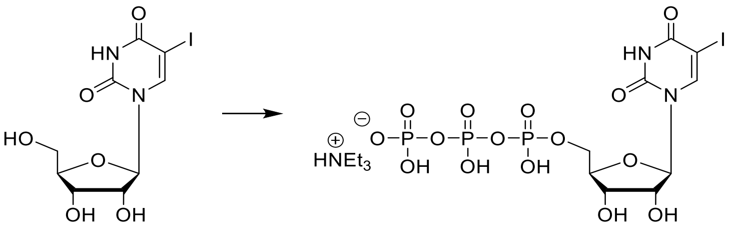

11 **21**

IUTP **21** was synthesized according to general procedure 2. 5-Iodouridine **11** (500 mg, 1.40 mmol, 1.00 equiv) was dissolved in trimethyl phosphate (3 mL). Phosphorus oxychloride (150 µL, 1.60 mmol, 1.20 equiv) was added dropwise. Tris(tetrabutylammonium) hydrogen pyrophosphate (1.04 g, 1.10 mmol, 0.85 equiv) and 1.93 mL tributylamine (1.50 g, 8.10 mmol, 6.00 equiv) in 1.5 mL dry acetonitrile were added.

The product (4.0 TEA, 216 mg, 211 µmol, 16%) was obtained as a colorless solid.

^{1}H-NMR (400 MHz, D_2O): δ [ppm] = 8.29 (s, 1H), 5.95 (d, $^3J_{HH}$ = 5.5 Hz, 1H), 4.47 (t, $^3J_{HH}$ = 3.9 Hz, 1H), 4.41 (t, $^3J_{HH}$ = 4.6 Hz, 1H), 4.32 – 4.19 (m, 3H), 3.20 (q, $^3J_{HH}$ = 7.3 Hz, 25H), 1.28 (t, $^3J_{HH}$ = 7.3 Hz, 36H). **^{13}C-NMR** (101 MHz, D_2O): δ [ppm] = 170.5, 157.6, 145.1, 88.3, 83.1, 73.5, 69.4, 64.9, 59.0. **^{31}P-NMR** (162 MHz, D_2O): δ [ppm] = -7.74 (d, $^2J_{PP}$ = 20.9 Hz), -11.73 (d, $^2J_{PP}$ = 20.4 Hz), -22.77 (dd, $^2J_{PP}$ = 20.4 Hz). **HRMS** (ESI$^-$): *m/z* cal: 608.8579 [M-H]$^-$, found: 608.8571.

2.3.3 Preparation of AP-CTP (22)

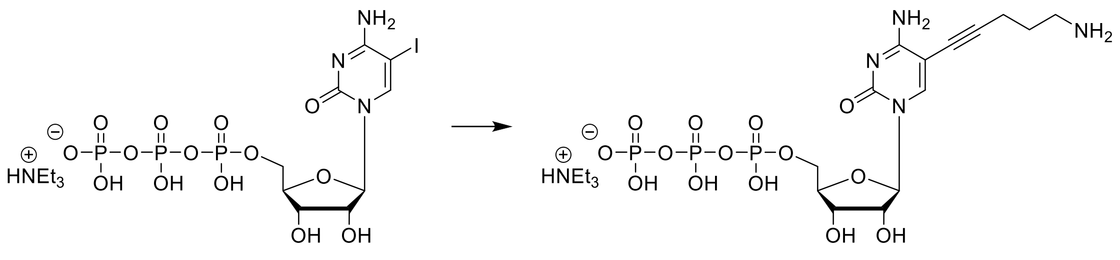

20 **22**

AP-CTP **22** was synthesized according to general procedure 3 with ICTP **20** (180 mg, 160 µmol, 1.00 equiv), tri(2-furyl)phosphine (4.50 mg, 19.0 µmol, 12 mol%), tris(dibencylideneacetone)dipalladium(0) (8.0 mg, 8.70 µmol, 5.4 mol%), copper(I) iodide (4.40 mg, 23.0 µmol, 15 mol%), triethylamine (220 µL, 1.60 mmol, 7.00 equiv), and pent-4-yn-1-amine (27.0 mg, 320 mmol, 1.50 equiv) in DMSO (2.5 mL).

The product (4.3 TEA, 120 mg, 120 µmol, 73%) was obtained as a light-yellow solid.

^{1}H-NMR (MHz, D_2O): δ [ppm] = 8.26 (s, 1H), 5.98 (d, $^{3}J_{HH}$ = 3.3 Hz, 1H), 4.44 (t, $^{3}J_{HH}$ = 5.8 Hz, 1H), 4.37 – 4.28 (m, 3H), 4.27 – 4.23 (m, 1H), 3.22 – 3.16 (m, 2H), 3.11 (q, $^{3}J_{HH}$ = 7.4 Hz, 27H), 2.65 – 2.57 (m, 2H), 1.99 – 1.90 (m, 2H), 1.24 (t, $^{3}J_{HH}$ = 7.3 Hz, 38H). **^{13}C-NMR** (101 MHz, D_2O): δ [ppm] = 165.1, 156.2, 144.0, 95.5, 93.0, 89.6, 82.4, 74.7, 72.1, 68.2, 63.9, 38.4, 25.3, 16.2. **^{31}P-NMR** (162 MHz, D_2O): δ [ppm] = -6.15 (d, $^{2}J_{PP}$ = 21.0 Hz), -10.98 (d, $^{2}J_{PP}$ = 19.3 Hz), -21.99 (dd, $^{2}J_{PP}$ = 20.3 Hz). **HRMS** (ESI^{-}): *m/z* cal: 563.0351 [M-H]$^{-}$, found: 563.0351.

2.3.4 Preparation of AP-UTP (23)

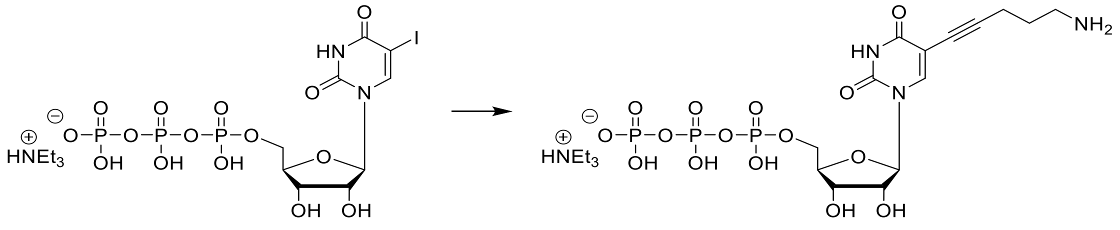

AP-UTP **23** was synthesized according to general procedure 3 with IUTP **21** (130 mg, 130 µmol, 1.00 equiv), tri(2-furyl)phosphine (3.50 mg, 15.0 µmol, 12 mol%), tris(dibencylideneacetone)dipalladium(0) (6.02 mg, 6.80 µmol, 5.4 mol%), copper(I) iodide (3.40 mg, 18.0 µmol, 15 mol%), triethylamine (180 µL, 1.30 mmol, 7.00 equiv), and pent-4-yn-1-amine (21.0 mg, 250 µmol, 1.50 equiv) in DMSO (2.5 mL).

The product (4.2 TEA, 90.0 mg, 91.0 µmol, 72%) was obtained as a light-yellow solid.

^{1}H-NMR (400 MHz, D_2O): δ [ppm] = 8.15 (s, 1H), 6.02 (d, $^{3}J_{HH}$ = 4.3 Hz, 1H), 4.46 (t, $^{3}J_{HH}$ = 5.3 Hz, 1H), 4.34 (t, $^{3}J_{HH}$ = 4.7 Hz, 1H), 4.31 – 4.23 (m, 3H), 3.20 (q, $^{3}J_{HH}$ = 7.3 Hz, 27H), 2.58 (t, $^{3}J_{HH}$ = 6.4 Hz, 2H), 1.94 (p, $^{3}J_{HH}$ = 6.7 Hz, 2H), 1.28 (t, $^{3}J_{HH}$ = 7.4 Hz, 38H). **^{13}C-NMR** (101 MHz, D_2O): δ [ppm] = 174.3, 158.1, 142.8, 92.2, 88.7, 82.4, 76.1, 75.2, 74.4, 68.8, 64.2, 46.4, 38.6, 25.5, 16.2, 8.5. **^{31}P-NMR** (162 MHz, D_2O): δ [ppm] = -6.18 (d, $^{2}J_{PP}$ = 21.1 Hz), -11.04 (d, $^{2}J_{PP}$ = 19.9 Hz), -22.03 (dd, $^{2}J_{PP}$ = 20.6 Hz). **HRMS** (ESI^{-}): *m/z* cal: 564.0191 [M-H]$^{-}$, found: 564.0195.

2.3.5 Preparation of 2TCO*a*-CTP (24)

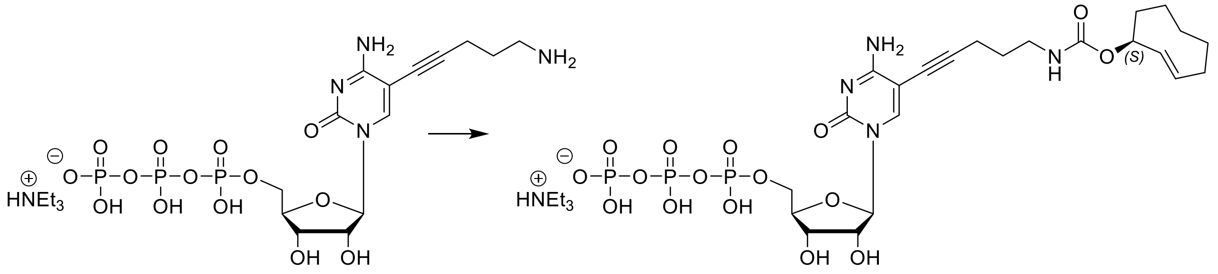

2TCO*a*-CTP **24** was synthesized according to general procedure 4 with AP-CTP **22** (40.0 mg, 54.0 µmol, 1.00 equiv), triethylamine (26.0 µL, 190 µmol, 3.50 equiv), and 2TCO*a*-NHS (20.0 mg, 75.0 µmol, 1.40 equiv) at 40 °C for 2 h.

The product (3.0 TEA, 39.0 mg, 38.0 µmol, 71%) was obtained as a light-yellow solid.

^{1}H-NMR (600 MHz, MeOD): δ [ppm] = 8.07 (s, 1H), 5.96 (s, 1H), 5.83 (dd, $^3J_{HH}$ = 13.1 Hz, 1H), 5.53 (d, $^3J_{HH}$ = 16.2 Hz, 1H), 5.23 (bs, 1H), 4.33 – 4.28 (m, 3H), 4.20 – 4.14 (m, 2H), 3.27 (quin, $^3J_{HH}$ = 6.2 Hz, 2H), 3.19 (q, $^3J_{HH}$ = 7.2 Hz, 17.9H), 2.53 – 2.49 (m, 2H), 2.45 – 2.43 (m, 1H), 2.04 – 1.99 (m, 2H), 1.98 – 1.94 (m, 1H), 1.88 – 1.83 (m, 1H), 1.77 (quin, $^3J_{HH}$ = 6.7 Hz, 2H), 1.74 – 1.68 (m, 1H), 1.65 – 1.60 (m, 1H), 1.52 – 1.45 (m, 1H), 1.31 (t, $^3J_{HH}$ = 7.2 Hz, 27.5H), 1.19 – 1.12 (m, 1H), 0.89 – 0.83 (m, 1H). **^{13}C-NMR** (151 MHz, MeOD): δ [ppm] = 162.6, 158.5, 150.7, 144.9, 132.9, 132.5, 99.2, 97.4, 91.2, 84.7, 75.9, 75.4, 75.0, 70.8, 66.2, 47.2, 41.7, 40.6, 37.0, 36.8, 30.1, 29.7, 25.2, 17.6. **^{31}P-NMR** (243 MHz, MeOD): δ [ppm] = -10.28 (bs), -11.23 (bs), -23.64 (bs). **HRMS** (ESI$^-$): *m/z* cal: 715.1188 [M-H]$^-$, found: 715.1184.

2.3.6 Preparation of 2TCO*a*-UTP (26)

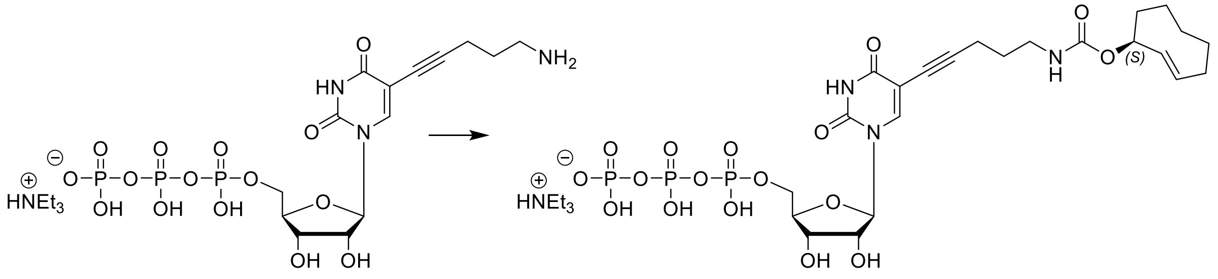

2TCO*a*-UTP **26** was synthesized according to general procedure 4 with AP-CTP **23** (124 mg, 117 µmol, 1.00 equiv), triethylamine (68.0 µL, 490 µmol, 4.20 equiv), and 2TCO*a*-NHS (43.0 mg, 163 µmol, 1.40 equiv) at 40 °C for 2 h.

The product (2.8 TEA, 81.0 mg, 80.0 µmol, 69%) was obtained as a white solid.

^{1}H-NMR (600 MHz, MeOD): δ [ppm] = 8.12 – 8.04 (m, 1H), 6.00 – 5.95 (m, 1H), 5.84 – 5.72 (m, 1H), 5.69 – 5.55 (m, 1H), 5.49 – 5.31 (m, 1H), 4.46 – 4.35 (m, 2H), 4.32 – 4.20 (m, 3H), 3.34 – 3.25 (m, 2H),

3.21 (q, $^3J_{HH}$ = 7.3 Hz, 18H), 2.54 – 2.45 (m, 2H), 2.42 – 2.32 (m, 1H), 2.22 – 2.03 (m, 2H), 2.03 – 1.89 (m, 1H), 1.86 – 1.73 (m, 3H), 1.69 – 1.55 (m, 2H), 1.54 – 1.44 (m, 2H), 1.29 (t, $^3J_{HH}$ = 7.3 Hz, 25H), 0.92 – 0.71 (m, 1H). **^{13}C-NMR** (151 MHz, MeOD): δ [ppm] = 164.6, 158.5, 150.8, 143.3, 133.4, 118.3, 100.8, 96.1, 88.4, 83.5, 73.7, 69.7, 65.1, 46.6, 40.2, 35.2, 28.3, 27.7, 27.4, 25.8, 25.3, 22.7, 21.0, 16.8, 8.2. **^{31}P-NMR** (243 MHz, MeOD): δ [ppm] = -8.20 (d, $^2J_{PP}$ = 19.5 Hz), -11.45 (d, $^2J_{PP}$ = 20.1 Hz), -22.53 (t, $^2J_{PP}$ = 19.7, 18.1 Hz). **HRMS** (ESI$^-$): *m/z* cal: 718.1174 [M+H], found: 718.1185.

2.3.7 Preparation of BCN-CTP (25)

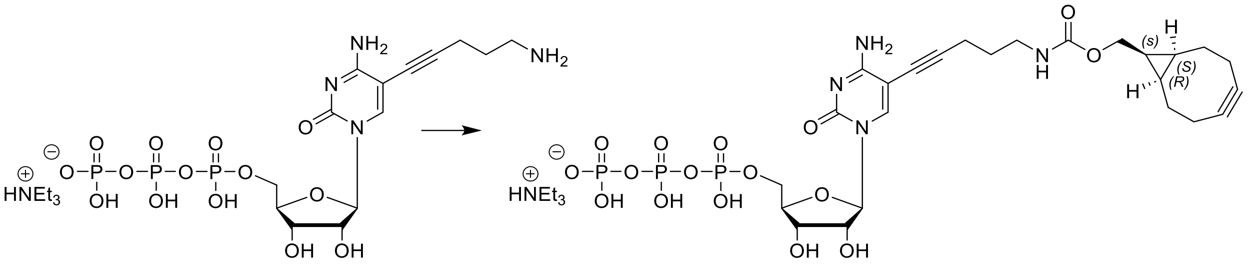

22 **25**

BCN-CTP **25** was synthesized according to general procedure 4 with AP-CTP **22** (118 mg, 118 µmol, 1.00 equiv), triethylamine (68.7 µL, 495 µmol, 3.50 equiv), and BCN-NHS (38.3 mg, 165 µmol, 1.40 equiv) at 40 °C for 2 h.

The product (4.0 TEA, 55.0 mg, 47.9 µmol, 41%) was obtained as an off-white solid.

^{1}H-NMR (400 MHz, D_2O): δ [ppm] = 8.12 (s, 1H), 5.97 (d, $^3J_{HH}$ = 4.6 Hz, 1H), 4.40 (t, $^3J_{HH}$ = 4.8 Hz, 1H), 4.32 (t, $^3J_{HH}$ = 4.8 Hz, 1H), 4.30 – 4.26 (m, 3H), 4.12 (d, $^3J_{HH}$ = 8.2 Hz, 2H), 3.34 – 3.27 (m, 2H), 3.21 (q, $^3J_{HH}$ = 7.3 Hz, 24H), 2.53 (t, $^3J_{HH}$ = 6.5 Hz, 3H), 2.27 – 2.16 (m, 6H), 1.81 (p, $^3J_{HH}$ = 6.4 Hz, 2H), 1.58 – 1.46 (m, 2H), 1.29 (t, $^3J_{HH}$ = 7.3 Hz, 36H), 0.89 (q, $^3J_{HH}$ = 9.0 Hz, 2H). **^{13}C-NMR** (101 MHz, D_2O): δ [ppm] = 165.1, 156.2, 143.6, 100.2, 97.6, 93.7, 89.4, 82.8, 74.3, 69.1, 64.8, 63.5, 46.6, 40.3, 28.5, 27.3, 20.8, 19.3, 17.2, 8.2. **^{31}P-NMR** (162 MHz, D_2O): δ [ppm] = -10.90 (d, $^2J_{PP}$ = 20.1 Hz), -11.53 (d, $^2J_{PP}$ = 20.8 Hz), -23.35 (dd, $^2J_{PP}$ = 20.1 Hz). **HRMS** (ESI$^-$): *m/z* cal: 739.1188 [M-H]$^-$, found: 739.1200.

2.3.8 Preparation of BCN-UTP (27)

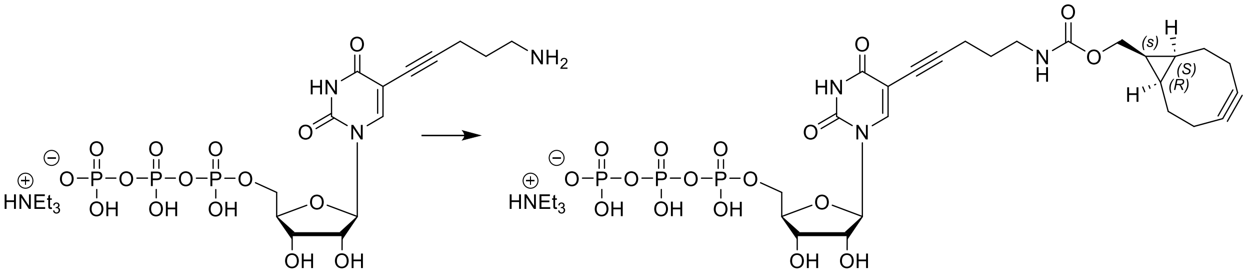

23 **27**

BCN-UTP **27** was synthesized according to general procedure 4 with AP-UTP **23** (90.0 mg, 89.7 µmol, 1.00 equiv), triethylamine (52.3 µL, 377 µmol, 4.20 equiv), and BCN-NHS (29.2 mg, 126 µmol, 1.40 equiv) at 40 °C for 2 h.

The product (3.4 TEA, 34.0 mg, 31.3 µmol, 35%) was obtained as an off-white solid.

^{1}H-NMR (400 MHz, D_2O): δ [ppm] = 8.10 (s, 1H), 5.98 (d, $^3J_{HH}$ = 5.5 Hz, 1H), 4.46 – 4.38 (m, 2H), 4.31 – 4.22 (m, 3H), 4.15 (d, $^3J_{HH}$ = 8.3 Hz, 2H), 3.33 – 3.26 (m, 2H), 3.21 (q, $^3J_{HH}$ = 7.3 Hz, 21H), 2.50 (t, $^3J_{HH}$ = 6.8 Hz, 2H), 2.29 – 2.18 (m, 6H), 1.80 (p, $^3J_{HH}$ = 6.3 Hz, 2H), 1.61 – 1.50 (m, 2H), 1.29 (t, $^3J_{HH}$ = 7.3 Hz, 31H), 0.98 – 0.89 (m, 2H).**^{13}C-NMR** (101 MHz, D_2O): δ [ppm] = 150.8, 143.3, 100.6, 88.4, 73.7, 69.7, 65.1, 63.5, 46.6, 40.1, 28.2, 22.5, 19.7, 17.2, 17.0, 8.2. **^{31}P-NMR** (16f2 MHz, D_2O): δ [ppm] = -10.96 (d, $^2J_{PP}$ = 20.0 Hz), -11.61 (d, $^2J_{PP}$ = 20.9 Hz), -23.36 (dd, $^2J_{PP}$ = 20.1 Hz). **HRMS** (ESI$^-$): *m/z* cal: 741.1101[M], found: 741.1082.

2.4 Synthesis of TCO-Probe

2.4.1 Preparation of 7-isocyanato-4-methylcoumarin (38)

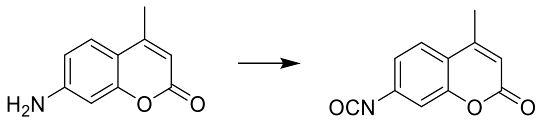

37 **38**

7-Isocyanato-4-methylcoumarin was prepared according to a published procedure[6] with 7-amino-4-methylcoumarin (970 mg, 5.54 mmol, 1.00 equiv), triphosgene (592 mg, 1.99 mmol, 0.40 equiv), and triethylamine (1.54 mL, 11.1 mmol, 2.00 equiv) in DCM (30 mL).

The product (650 mg, 3.23 mmol, 58%) was obtained as a beige solid. The analytical data agreed with those described in literature.[6]

2.4.2 Preparation of 2TCO*a*(4-methylcoumarin-7-yl)carbamate (39)

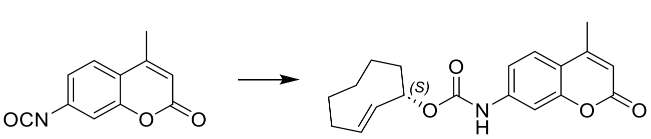

38 **39**

2TCO*a*(4-methylcoumarin-7-yl)carbamate was synthesized by a modified literature procedure.[7]

Sodium hydride (6.1 mg, 26 µmol, 1.1 equiv) was added to a solution of 2TCO*a*-OH (29 mg, 230 µmol, 1.0 equiv) in THF (2 mL) and the resulting mixture was stirred for 15 min at rt. The suspension was then dropwise added to a solution of 7-isocyanato-4-methylcoumarin (70 mg, 35 µmol, 1.5 equiv) in THF (2 mL), and the reaction was stirred for 16 h before it was quenched by the addition of Dowex

(50WX8 hydrogen form, 200-400 mesh), until a slightly acidic pH was reached. The suspension was filtered, the filter cake was washed with DCM and the filtrate was evaporated under reduced pressure. The residue was purified by flash chromatography (PE/EE 7:3 v/v, dryload) and further purified by pTLC (PE/EE 1:1 v/v).

The product (30 mg, 92 µmol, 40%) was obtained as a colorless solid. The analytical data agreed with those described in literature.[7]

3. Cell Experiments

3.1 Cell Culture

Vero B4 cells (DSMZ no. ACC 33) were cultured under sterile conditions in Dulbecco's modified Eagle's medium ([+] 4.5 g/L D-glucose, L-Glutamine, [+] Pyruvate, DMEM, *Gibco^{TM}*) supplemented with 10% fetal calf serum (HI FBS, *Gibco^{TM}*) and 1 U/mL Penicillin-Streptomycin (*Gibco^{TM}*) at 37 °C, 5 % CO_2 and 90% humidity.

3.2 Procedures

3.2.1 Procedure for Isolation of RNA labeled with Tri*PPP*ro 3 and NTP 25

Generally, 1,5 x 10^5 Vero cells were seeded in petri dishes (60/15 mm, *Greiner Bio-One)* and incubated at 37 °C, 5 % CO_2, and 90% humidity for two days in DMEM to reach 80 – 90% confluency. For RNA-Labeling with Tri*PPP*ro, the medium was changed to VP-SFM (*Gibco™*) with one washing step, followed by the addition of the respective Tri*PPP*ro **3** (5 µM) in VP-SFM. The Tri*PPP*ro media was renewed after 120 min. After 2 more hours of incubation, the RNA-Isolation was performed as described below. For RNA-Labeling with NTP, the cells were washed with tricine buffer and then incubated with an equimolar solution of SNT-Transporter (60 µM, BioTracker NTP, *Sigma-Aldrich*), and NTP **25** (60 µM) in tricine buffer for 10 min at 37 °C, 5% CO_2, and 90% humidity. After that, the treatment solution was removed, cells were washed once with DMEM and then incubated for 4 h in DMEM at 37 °C, 5% CO_2, and 90% humidity before the RNA-Isolation could take place. For RNA-Isolation, the culture medium was removed, and 1 mL per 10 cm^2 TRIzol® Reagent (*Invitrogen*) was added. The lysate was transferred into an Eppendorf Tube® and incubated for 5 min at rt, followed by the addition of 200 µL chloroform per 1 mL TRIzol® Reagent. After mixing and incubating for 3 min at rt, the lysate was centrifuged at 4 °C and 12.000 x g for 15 min, whereby a 3-phase system is obtained. The upper aqueous phase was transferred into an Eppendorf Tube® and used for further purification with the RNA Clean and Concentrator-5 Kits (*Zymo Research*). The purification was carried out according to the protocol of the Kit, 10 µL RNase- and DNase-free water was used to elute the RNA.

For negative control, cells were cultivated without the addition of NTP or Tri*PPP*ro under the aforementioned labeling procedures. In the case of the Tri*PPP*ros, the corresponding amount of DMSO was added. Further work-up and RNA-Isolation of the control experiment were carried out according to the procedure described above.

3.2.2 Procedure for the Click Reaction, following RNA-Isolation

For the click reaction, a 4 mM stock solution of tetrazine-fluorophore conjugate **5** in DMSO was prepared. The isolated RNA was incubated with 5 µM dye **5** in RNase- and DNase-free water, containing 1% DMSO. The click reaction was incubated for 18 h at rt. Excess dye was removed from the solution by purifying the isolated RNA with the Zymo Clean and Concentrator 5-Kit and eluted with 10 µL of RNase- and DNase-free water, followed by the determination of the concentration. Fluorescence spectroscopy was performed using an excitation wavelength of $\lambda_{exc.}$ = 437 nm and 5 nm slits for both excitation and emission.

3.3.3 Procedure for Fixed Cell Labeling with NTPs

Generally, 1.0 x 10^4 Vero cells were seeded in µ-slides 8-well (ibiTreat) and incubated at 37 °C, 5% CO_2 and 90% humidity for 24 h in DMEM. For RNA-Labeling with NTP, the cells were washed with tricine buffer and then incubated with an equimolar solution of SNT-Transporter (60 µM, Bio Tracker NTP, *Sigma-Aldrich*) and NTP **25** (60 µM) in tricine buffer for 10 min at 37 °C, 5% CO_2, and 90% humidity. After that, the treatment solution was removed, cells were washed once with DMEM and then

incubated in DMEM at 37 °C, 5% CO_2, and 90% humidity for 4 h. Following incubation, cells were washed with PBS, fixed with 4% PFA for 15 min at rt, washed two times with PBS, and then permeabilized with Triton X-100 (0.1%, 20 min, rt). Subsequently, samples were washed three times with PBS, followed by incubation with fluorescent labeling solution containing 5 µM dye **5** overnight at 37°C. Following dye incubation, cells were incubated with Hoechst dye (nuclear counterstain) for 5 min. Afterwards, imaging was performed using a Leica DMI8, TCS SP8 inverse microscope with a HC PL APO 63x/1.40 OIL CS2 objective. Tetrazine-fluorophore conjugate **5** was excited using the 488 nm laser (10% intensity), and fluorescence emission was measured at 500–520 nm, complemented with a brightfield image.

3.3.4 Procedure for Fixed Cell Labeling with preclicked-NTPs

Generally, 1.0 x 10^4 Vero cells were seeded in µ-slides 8-well (ibiTreat) and incubated at 37 °C, 5% CO_2, and 90% humidity for 24 h in DMEM. For RNA-Labeling with preclicked-NTP, cells were washed with tricine buffer and then incubated with an equimolar solution of SNT-Transporter (35 µM, Bio Tracker NTP, *Sigma-Aldrich*) and preclicked-NTP **29** (35 µM) in tricine buffer for 10 min at 37 °C, 5% CO_2, and 90% humidity. After that, the treatment solution was removed, cells were washed once with DMEM, and then incubated in DMEM at 37 °C, 5% CO_2, and 90% humidity for 4 h. Following incubation, cells were washed with PBS, fixed with 4% PFA for 15 min at rt, washed two times with PBS, and then permeabilized with Triton X-100 (0.1%, 20 min, rt). Subsequently, samples were washed three times with PBS, followed by incubation with Hoechst dye (nuclear counterstain) for 5 min. Afterwards, imaging was performed using a Leica DMI8, TCS SP8 inverse microscope with a HC PL APO 63x/1.40 OIL CS2 objective. Tetrazine-fluorophore conjugate **5** was excited using the 488 nm laser (10% intensity), and fluorescence emission was measured at 500–520 nm, complemented with a brightfield image.

Additional information: The preclicked-NTP **29** was obtained by incubating an equimolar amount of NTP **25** and dye **5** (70 µM) in water with a minimum of 1% DMSO overnight. The solvent was then removed in a SpeedVac, and the preclicked-NTP was dissolved in tricine buffer at the desired concentration.

3.3.5 Procedure for Live Cell Labeling with Tri*PPP*ros

Vero cells (2.5 × 10^4 cells per well) were seeded into an ibidi 8-well imaging chamber and cultured overnight at 37°C 5% CO_2. The following day, the medium was replaced with VP Serum-Free Medium (VP SFM). As a transcription inhibition control, selected wells were treated with 5 µg/ml actinomycin D (ActD). Cells were then incubated for a total duration of 3 h with 5 µM Tri*PPP*ro-modified nucleotides (**1** - **4**) diluted in VP SFM, unless stated otherwise. Following incubation, cells were washed three times with VP SFM and, where appropriate, ActD treatment was renewed. Cells were subsequently incubated with fluorescent labeling solution containing 5 µM dye **5** and Hoechst 33342 (Cat. No. 14533-100MG, *Merck*) for 30 min at 37°C. Immediately following dye incubation, imaging was performed using a Nikon Ti2 spinning disk fluorescence microscope equipped with a Yokogawa CSU-W1 SoRa unit, Hamamatsu C14440-20UP camera with a gain setting of 1× and auto-exposure enabled, and a 100× Apo TIRF objective (NA 1.49, Nikon). The system included laser lines at 405, 445, 488, 515, 561, and 638 nm, along with standard filter sets. Hoechst fluorescence was detected using the 405 nm laser line (50% intensity, 200 ms exposure time), while dye **5** was imaged using the 488 nm line (80% intensity, 600 ms exposure time). Physiological conditions were maintained at 37 °C with 5% CO_2 using an environmental control system.

A549 and PK15 cells were treated identically.

3.3.6 Procedure for rRNA Counterstain

Following live-cell imaging, cells were fixed in 4% paraformaldehyde (PFA) for 15 min at rt and washed twice with PBS. Permeabilization was performed using 0.1% Triton X-100 in PBS for 20 min at rt, followed by blocking with 3% bovine serum albumin (BSA) in PBS for 15 min at rt. Subsequently, cells were incubated with primary antibody targeting whole 5.8S ribosomal RNA (1:250 dilution in 3% BSA, rRNA monoclonal antibody (Y10b) (Cat. No. MA1-16628, LOT AB4638411, mouse IgG3, *Invitrogen*) for 1 h at rt, washed three times with PBS, and then incubated with fluorescently labeled secondary antibody (1:1000 dilution in PBS, Goat anti-Mouse IgG (H+L) Highly Cross-Adsorbed Secondary Antibody, Alexa Fluor™ 647 (Cat. No. A21236, LOT 2482947, *Invitrogen*)) for 1 h at rt. After three additional washes in PBS, imaging was performed as described in 3.3.5. The AF647 was excited with the 638 nm laser line (75% intensity, 200 ms exposure time).

3.3.7 Procedure for Live Cell Labeling with MitoTracker DeepRed™

Vero cells (3.5×10^4 cells per dish) were seeded into 35 mm ibidi imaging dishes and cultured overnight at 37 °C with 5% CO_2. Tri*PPP*ro labeling was performed as described in Section 3.3.5. Mitochondria were stained with 100 nM MitoTracker DeepRed™ (Cat. No. M22426, *Thermo Fisher*) for 30 min at 37 °C, followed by three washes with VP SFM. For fluorescent tagging of Tri*PPP*ro-modified RNA, cells were incubated with 5 µM **5** and Hoechst 33342 (Cat. No. 14533-100MG, *Merck*) for 30 min at 37 °C. Live-cell imaging was performed immediately on the Nikon Ti2 spinning-disk system (see Section 3.3.5). Acquisition settings: Hoechst, 405 nm (50% intensity, 200 ms); MitoTracker, 638 nm (50% intensity, 400 ms); dye 5, 488 nm (80% intensity, 600 ms).

3.3.8 Procedure for Live Cell Labeling with Tri*PPP*ro vs. SNTT transporter

Vero cells (2.5×10^4 cells per well) were seeded into an ibidi 8-well imaging chamber and cultured overnight at 37 °C with 5% CO_2. For the Tri*PPP*ro condition, cells were processed as described in Section 3.3.5, except that the incubation with Tri*PPP*ro-modified nucleotides was shortened to 1 hour. For the SNTT transporter condition, cells were pulsed for 1 h with 5 µM SNTT and 5 µM **25** in Tricine buffer. After treatment, cells were washed three times with VP SFM, stained with 5 µM dye **5** and Hoechst 33342 for 30 min at 37 °C, and imaged as described in Section 3.3.5.

3.3.9 Procedure for Live Cell Labeling with Tri*PPP*ro with Lattice Lightsheet

Live-cell imaging was performed on a Zeiss Lattice Lightsheet 7 microscope equipped with two Hamamatsu ORCA-Fusion BT sCMOS cameras, a 13.3× NA 0.4 illumination objective, and a 44.83× NA 1.0 detection objective. The system employed 488 nm, 561 nm, and 640 nm laser lines with corresponding emission filters, a 30 × 1,000 µm light sheet optimized to minimize side lobes, and a 565 nm beam splitter. Environmental conditions were maintained at 37 °C and 5% CO_2 throughout the acquisition process. Time-lapse volumes were recorded every hour for 48 hours using 488 nm (5%) and 640 nm (1%) lasers for illumination. Each volume comprised 300 frames spanning a total depth of 120 µm, with a 100 ms exposure per frame. Acquired datasets were deskewed using the cover glass transformation method and deconvoluted with the constrained iterative algorithm (automatic strength) implemented in ZEN v. 3.12 (Zeiss).

3.3.10 Statistical analysis

Regions of interest (ROIs) were defined and measured in *ImageJ/Fiji*. Data visualization and statistical analyses were performed using GraphPad Prism. The number of analyzed samples (*n*) is specified in the respective figure legends. Boxplots display the median (line), interquartile range (box), and full data range (whiskers). Statistical significance was determined using the Kruskal–Wallis test followed by Dunn's multiple comparisons test. Significance notation: ns = not significant; $p < 0.05$ (*); $p < 0.01$ (**); $p < 0.001$ (***); $p < 0.0001$ (****).

4. Supplementary Figures

4.1 Turn-on values and kinetic studies

Spectroscopic monitoring of the fluorogenic click reaction and determination of its rate constant were performed using a 4.00 mM stock solution of tetrazine-fluorophore conjugate **5** prepared in DMSO and stored as aliquots at -20 °C. All nucleotide stock solutions were prepared at a concentration of 4.00 mM in ddH_2O and stored at -20 °C. The click reaction was performed in 1.00 cm path length quartz cuvettes (*Starna*) with a volume of 0.50 mL, using a DMSO/water mixture (1:99 v/v%) as the solvent. The dye **5** was diluted to 20.0 µM in water, containing 1% DMSO, and the emission spectra were recorded immediately ($\lambda_{exc.}$ = 437 nm, Slits: 2.50 nm excitation, 2.50 nm emission, T = 20 °C, $\lambda_{emi.}$ = 452 – 800 nm. To obtain the background spectrum for subtraction, 500 µL of a DMSO/water mixture (1:99) was measured. For time-resolved fluorescence measurement of the click reaction, 200 µM of the respective nucleotide and 20.0 µM of the dye **5** were mixed in DMSO/water (1:99, v/v%) to obtain the click product. Emission spectra were recorded until the maximum fluorescence intensity was observed. The time-resolved data of the click reaction were corrected against the fluorescence spectrum of the respective nucleotide dissolved in water containing 1% DMSO. The data was normalized to the corresponding observed fluorescence maxima. In order to determine the reaction rate constant, the area between 452-800 nm at the corresponding time stamp was plotted against time. A mono-exponential fit was applied based on the assumption of pseudo-first-order kinetics. This allowed the determination of the observed reaction rate constant (k_{obs}) by fitting the experimental data according to the following equation:

$$y = a + b * e^{-k_{obs} x}$$

To obtain the second-order rate constants (k_2), the following equation was applied to convert the observed pseudo-first-order rate constants:

$$k_2 = \frac{k_{obs}}{[NTP]_0}$$

4.1.1 Click compound 31 (BCN-UTP 27 + 5)

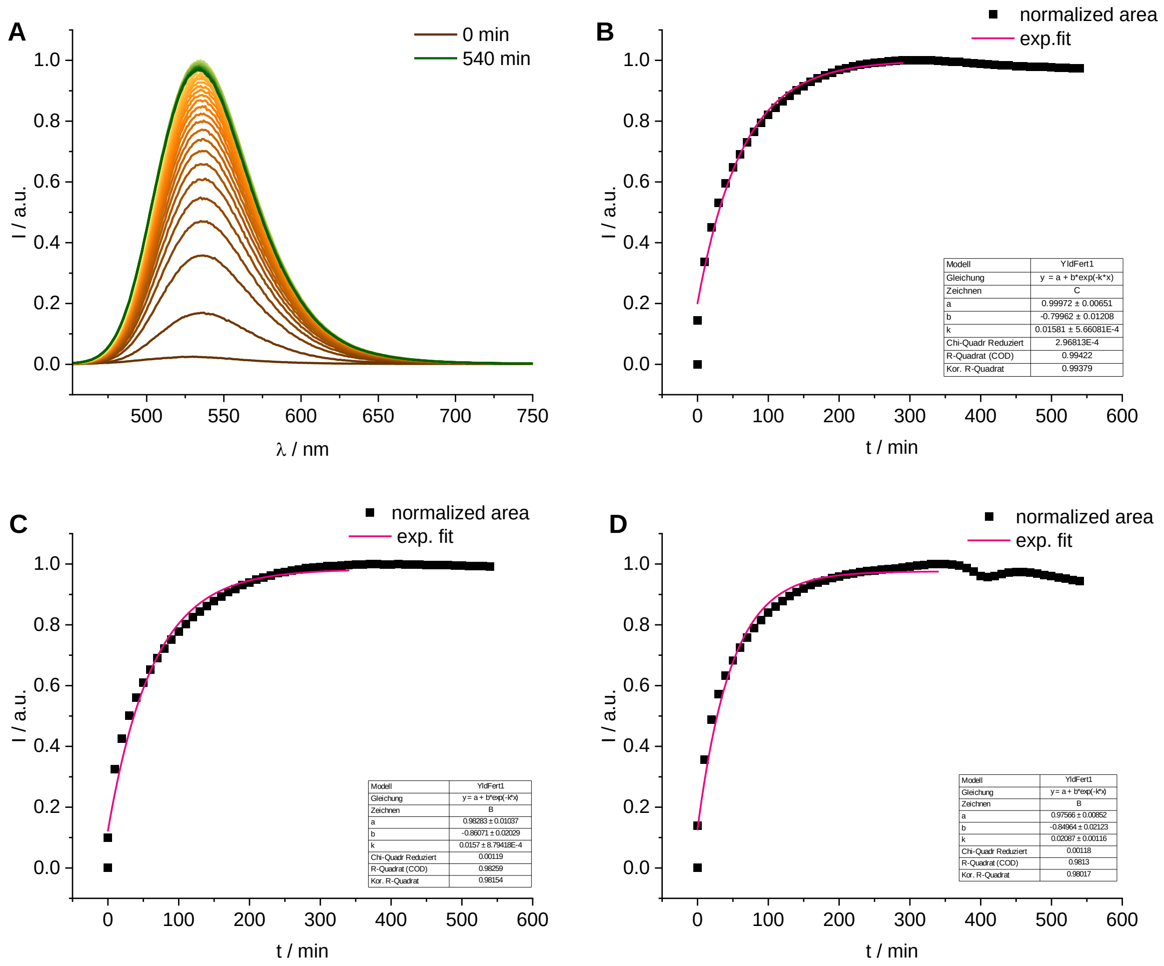

Figure S1: A) Fluorescence spectra with increasing reaction time of the click reaction between BCN-modified UTP **27** and the tetrazine-dye **5**. B) Kinetic data analysis by plotting the area between 452 – 800 nm against time. C) and D) Replicates of kinetic data analysis. Measurements were performed in water with 1% DMSO, λ_{ex} = 437 nm, 20 µM of dye **5** (1.00 equiv.), 200 µM **27** (10.0 equiv.), Slits: 2.5 nm excitation, 2.5 nm emission, T = 20 °C. Exponential fit function: y = a + b exp(-k_{obs}x) was used to determine the k_2.

4.1.2 Click compound 29 (BCN-CTP 25 + 5)

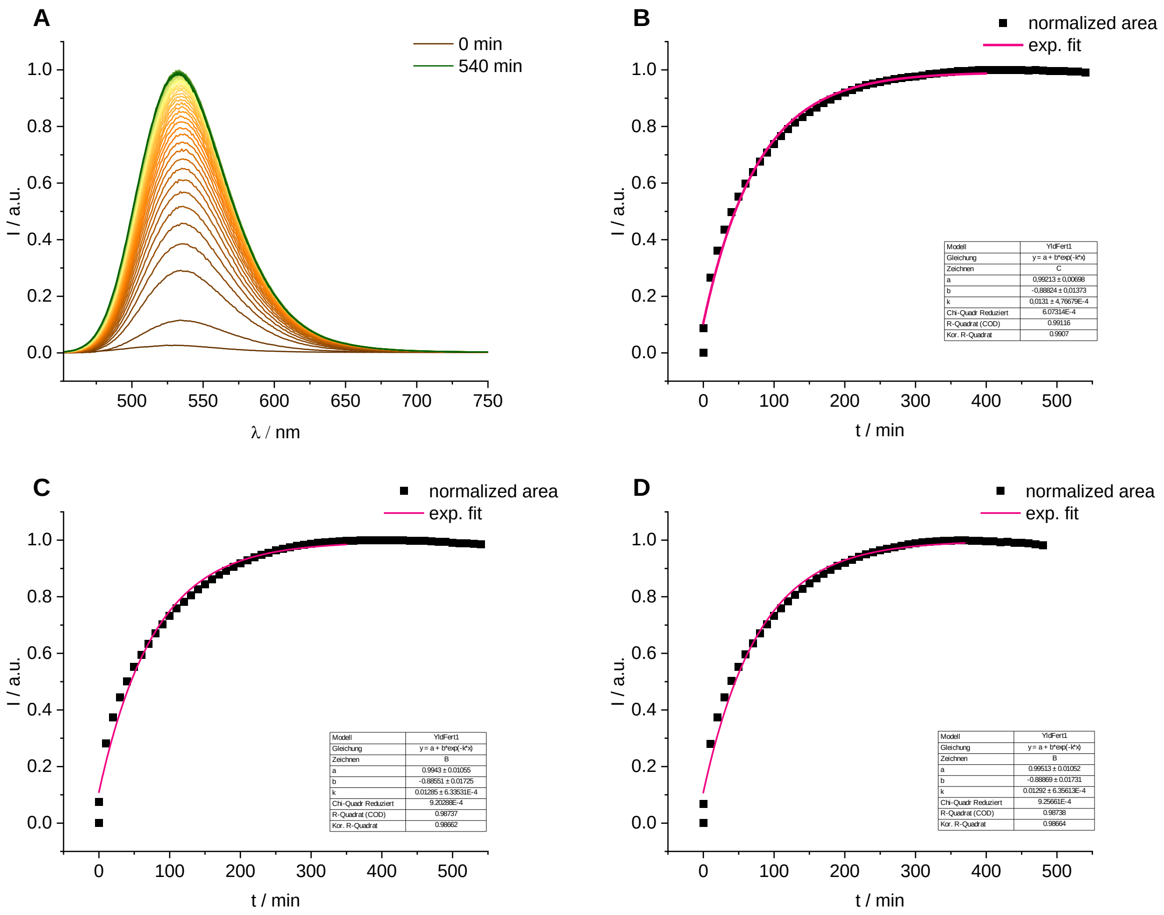

Figure S2: A) Fluorescence spectra with increasing reaction time of the click reaction between BCN-modified CTP **25** and the tetrazine-dye **5**. B) Kinetic data analysis by plotting the area between 452 – 800 nm against time. C) and D) Replicates of kinetic data analysis. Measurements were performed in water with 1% DMSO, λ_{ex} = 437 nm, 20 µM of dye **5** (1.00 equiv.), 200 µM **25** (10.0 equiv.), Slits: 2.5 nm excitation, 2.5 nm emission, T = 20 °C. Exponential fit function: $y = a + b \exp(-k_{obs}x)$ was used to determine the k_2.

4.1.3 Click compound 30 (2TCO*a*-UTP 26 + 5)

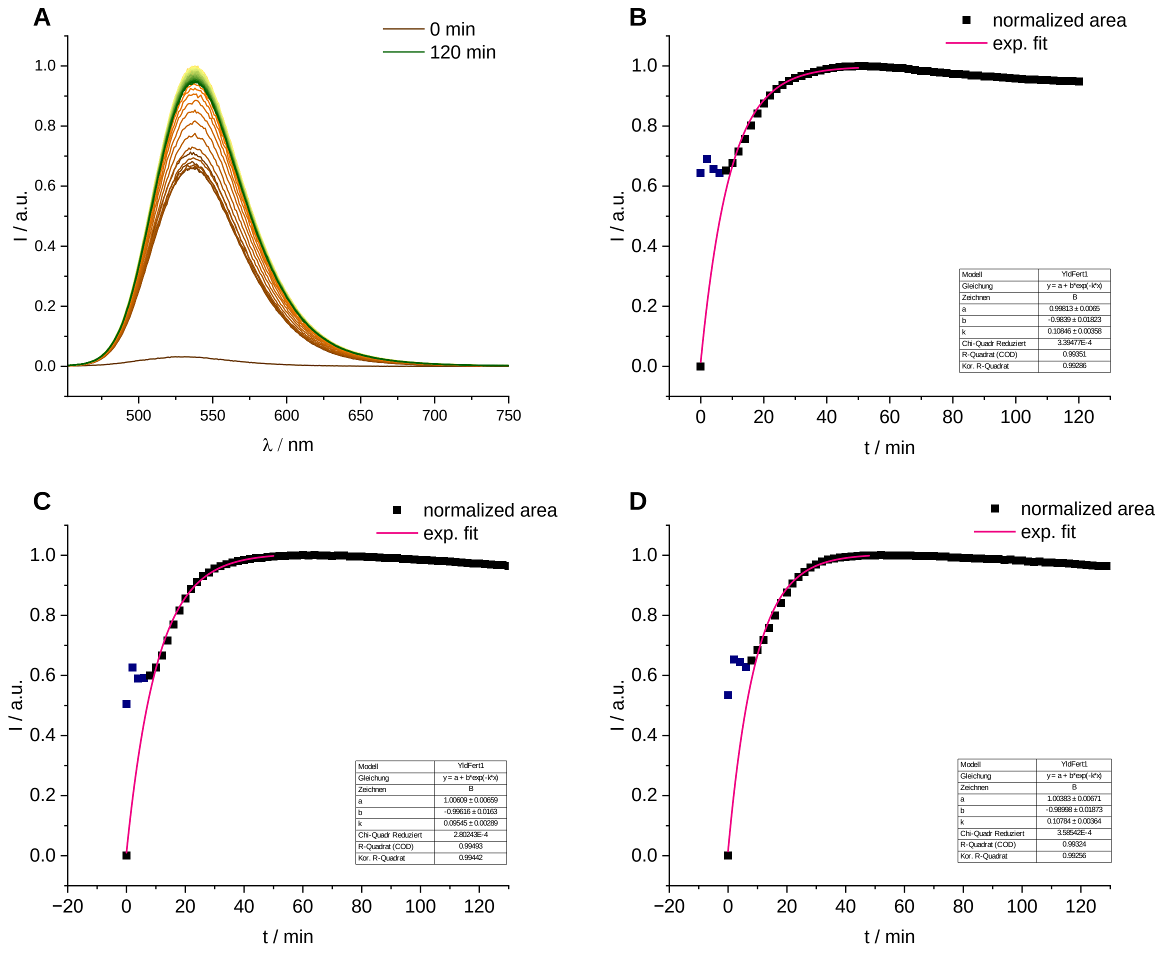

Figure S3: A) Fluorescence spectra with increasing reaction time of the click reaction between 2TCOa-modified UTP **26** and the tetrazine-dye **5**. B) Kinetic data analysis by plotting the area between 452 – 800 nm against time. C) and D) Replicates of kinetic data analysis. Measurements were performed in water with 1% DMSO, λ_{ex} = 437 nm, 20 µM of dye **5** (1.00 equiv.), 200 µM **26** (10.0 equiv.), Slits: 2.5 nm excitation, 2.5 nm emission, T = 20 °C. Exponential fit function: y = a + b exp(-k_{obs}x) was used to determine the k_2.

4.1.4 Click compound 28 (2TCO*a*-CTP 24 + 5)

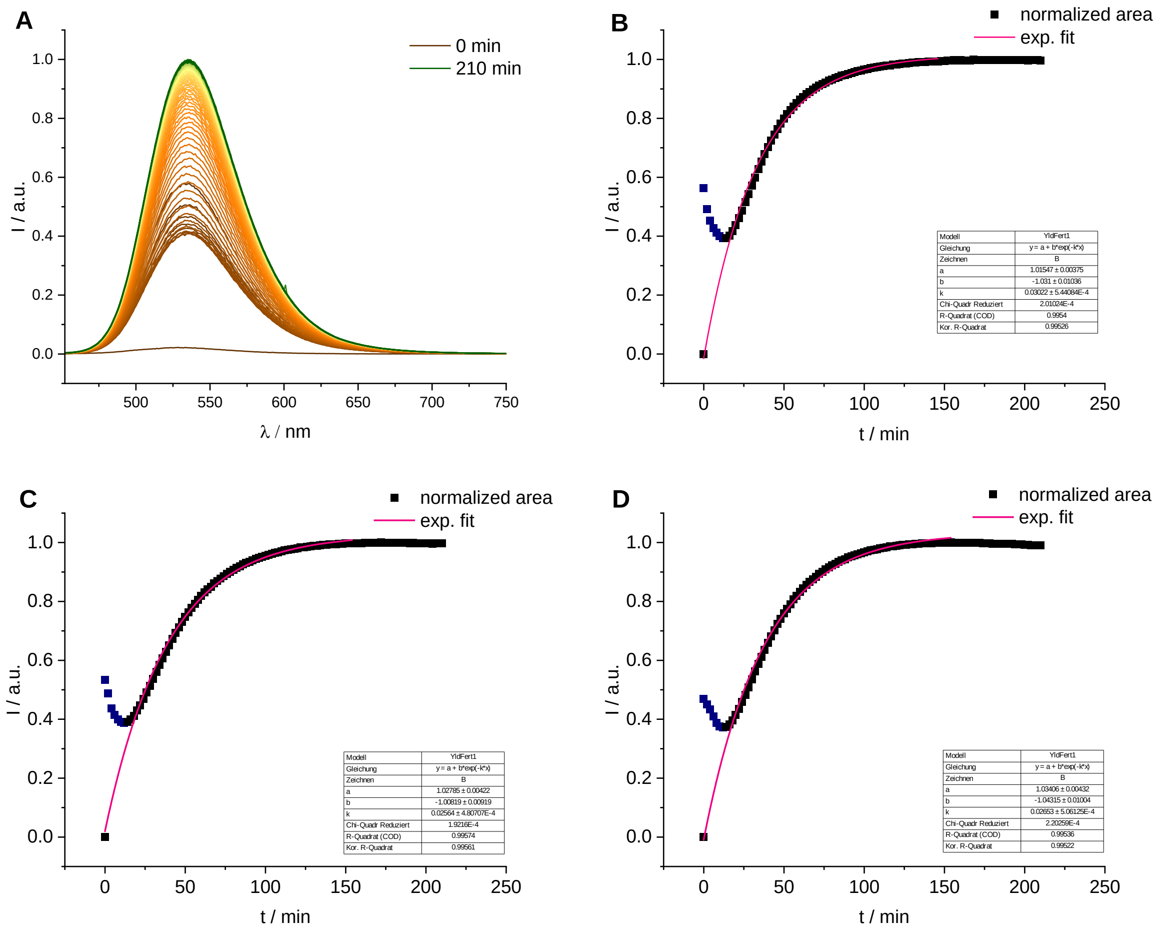

Figure S4: A) Fluorescence spectra with increasing reaction time of the click reaction between 2TCOa-modified CTP **24** and the tetrazine-dye **5**. B) Kinetic data analysis by plotting the area between 452 – 800 nm against time. C) and D) Replicates of kinetic data analysis. Measurements were performed in water with 1% DMSO, λ_{ex} = 437 nm, 20 µM of dye **5** (1.00 equiv.), 200 µM **23** (10.0 equiv.), Slits: 2.5 nm excitation, 2.5 nm emission, T = 20 °C. Exponential fit function: y = a + b exp(-k_{obs}x) was used to determine the k_2.

<u>Mass analysis of Click Reaction:</u>

Elimination product:

MS (ESI$^-$): *m/z* cal: 565.0424 [M+H]$^+$, found: 565.0472

Pyridazine click compound:

MS (ESI$^-$): *m/z* cal: 1055.2865 [M]$^+$, found: 1055.2878

4.1.5 Click reaction 4TCO-NHS-carbonate + 5

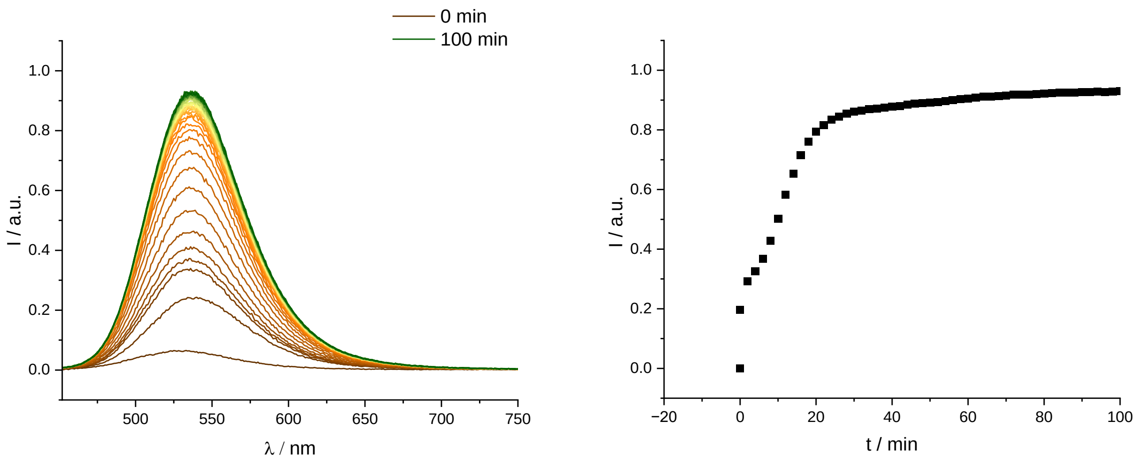

Figure S5: Fluorescence spectra with increasing reaction time of the click reaction between 4TCO-NHS-carbonate and the tetrazine-dye **5** (left). Area between 452 – 800 nm plotted against time (right). Measurements were performed in water with 1% DMSO, λ_{ex} = 437 nm, 20 µM of dye **5** (1.00 equiv.), 200 µM 4TCO-NHS-carbonate (10.0 equiv.), Slits: 2.5 nm excitation, 2.5 nm emission, T = 20 °C.

4.2 Elimination Assay

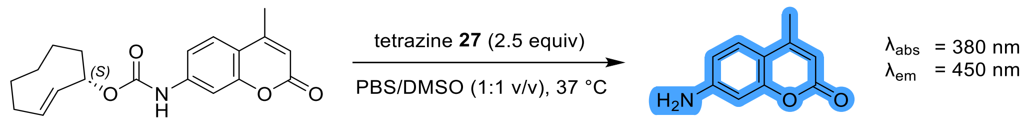

39 **37**

Elimination assay with the fluorogenic TCO-probe was carried out based on a previously published procedure[7] using the following stock solutions: tetrazine (6.45 mM in DMSO), TCO-probe (10 mM in DMSO), and 7-AC (10 mM in DMSO).

General procedure for creating the standard curve: tetrazine stock (3.88 µL) was mixed with DMSO (95.12 µL) and PBS (100 µL). 7-AC stock (1.00 µL) was added, and the mixture was diluted accordingly with PBS/DMSO 1:1 (v/v). The fluorescence was measured on a Cary Eclipse Fluorescence Spectrophotometer (*Agilent Technologies*) in single use makro cuvettes (*Brand*) with given concentrations and the following settings: excitation and emission slit 2.5 nm, PMT detector voltage 770 V. The samples were diluted with 1.8 mL PBS/DMSO 1:1 (v/v) prior to the measurement. The area of the fluorescence signal was determined between 385 nm and 500 nm. All measurements were carried out in triplicate. A standard curve was plotted using linear regression.

General procedure for measuring the extent of elimination: Tetrazine stock (3.88 µL) was mixed with DMSO (95.12 µL) and PBS (100 µL). TCO-probe stock (1.00 µL) was added, and the mixture was incubated at 37 °C. The fluorescence was measured on a Cary Eclipse Fluorescence Spectrophotometer (*Agilent Technologies*) in single use makro cuvettes (*Brand*) at given time points and the following settings: excitation and emission slit 2.5 nm, PMT detector voltage 770 V. The reaction mixtures were diluted with 1.8 mL PBS/DMSO 1:1 (v/v) prior to the measurement. The area

of the fluorescence signal was determined between 385 nm and 500 nm. The degree of elimination was then calculated using the standard curve. All measurements for all given time points were carried out in triplicate.

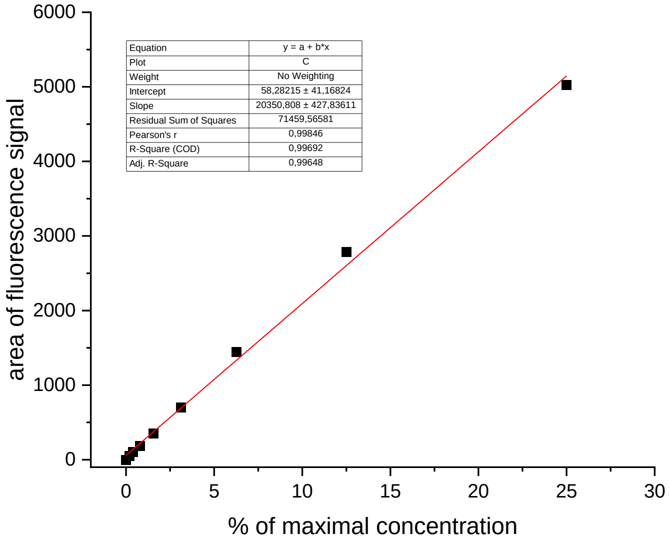

Figure S6: Calibration curve for determining of the degree of elimination in the reaction between the fluorogenic TCO-probe and the tetrazine dye. The area of the obtained fluorescence signal between 385 nm and 500 nm was plotted against the percentage of the maximal possible 7-aminocoumarin concentration in the elimination assay. Measurements were taken in the presence of the tetrazine dye to account for any possible effects on fluorescence. The standard curve was fitted by linear regression.

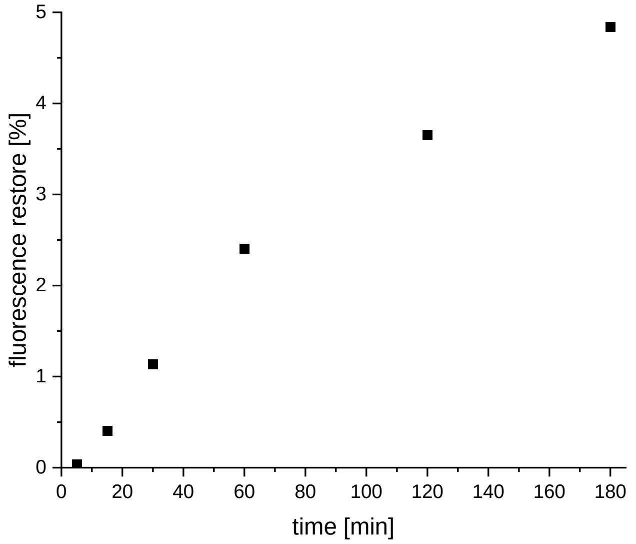

Figure S7: Time-dependent elimination of 7-aminocoumarin from the dihydropyridazine click-product of the TCO-probe and the tetrazine dye. The degree of elimination was calculated according to the calibration curve by determining the area of the fluorescence signal between 385 nm and 500 nm at given time points.

4.4 Cell Images

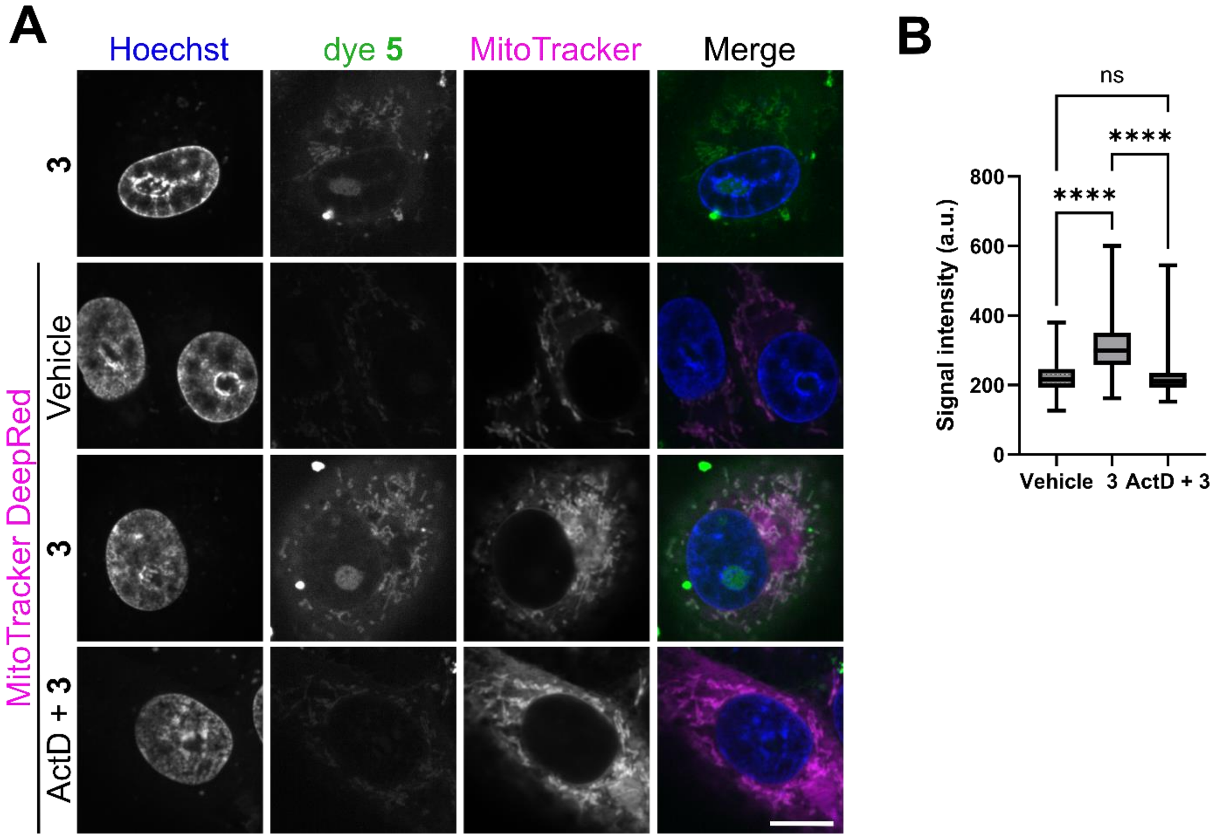

Figure S8: Detection of nascent RNA using BCN_CTP (3) and MioTracker DeepRed™. (**A**) Vero cells were washed with VP-SFM and incubated with 5 µM of **3** for 3 hours, if indicated this and the following steps in the presence of ActD. Following labeling, cells were washed three times with VP-SFM and subsequently incubated for 30 min with 100 nm MitoTracker DeepRed™ (ThermoFisher) to visualize mitochondria (magenta). After washing cells in VP-SFM, cells were incubated for 30 min with 5 µM tetrazine-fluorophore conjugate (**5**) to detect Tri*PPP*ro-tagged RNA (green), and Hoechst (blue) to label nuclei. Live-cell imaging was performed using a Nikon Ti2 spinning disk fluorescence microscope equipped with a 100× Apo TIRF objective. Nuclear outlines are circled (yellow). Individual channels are shown in greyscale, the merged image is shown in color. Scalebar, 10 µm. (**B**) MitoTracker-labeled mitochondria were thresholded in *ImageJ/Fiji* to generate binary masks, and corresponding signal intensities in the Tri*PPP*ro channel were measured within the defined mitochondrial regions. The median intensity per cell was determined, with at least 20 cells analyzed per condition. Boxplots represent the median (line), interquartile range (box), and full range (whiskers). Statistical significance was assessed using the Kruskal–Wallis test followed by Dunn's multiple comparisons test. Significance notation: ns = not significant; $p < 0.05$ (*); $p < 0.01$ (**); $p < 0.001$ (***); $p < 0.0001$ (****).

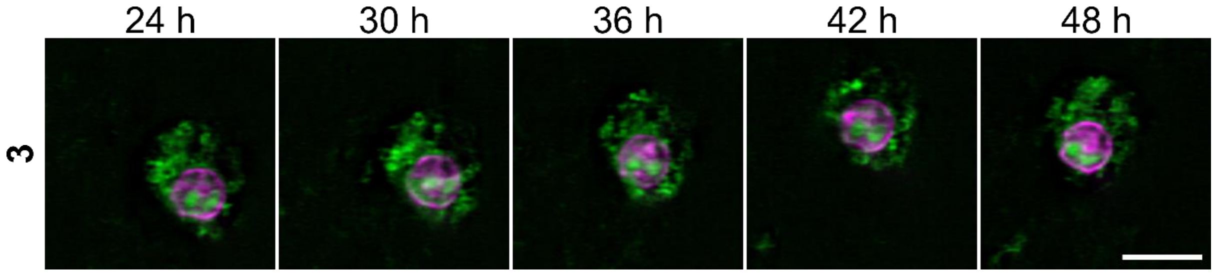

Figure S9: Time-lapse imaging of RNA-labeled Vero cells. Vero cells were washed with VP-SFM and incubated with 5 µM of **3**. After labeling, cells were washed three times with VP-SFM. After 30 min incubation with 5 µM tetrazine-fluorophore conjugate (**5**) to visualize Tri*PPP*ro-tagged RNA (green), and Hoechst (blue) to label nuclei, live-cell imaging was performed using a Zeiss Lattice Lightsheet 7, equipped with two Hamamatsu ORCA-Fusion BT sCMOS cameras, a 13.3× NA=0.4 illumination objective, and a 44.83× NA=1.0 detection objective. The setup included 488/561/640 nm laser lines with corresponding filter sets. A 30 × 1,000 µm light sheet, free from side lobes, and a 565 nm beam splitter were used. The environmental control system maintained physiological growth conditions at 37 °C with 5% CO_2. Acquired volumes (300 frames, 120 µm total, 100 ms exposure) were recorded every 1 h for 48 h using 488 nm (5%) and 640 nm (1%) lasers for illumination. Volumes were deskewed using the cover glass transformation method and deconvoluted (automatic strength, constrained iterative algorithm) in ZEN v. 3.12. Scale bar, 20 µm.

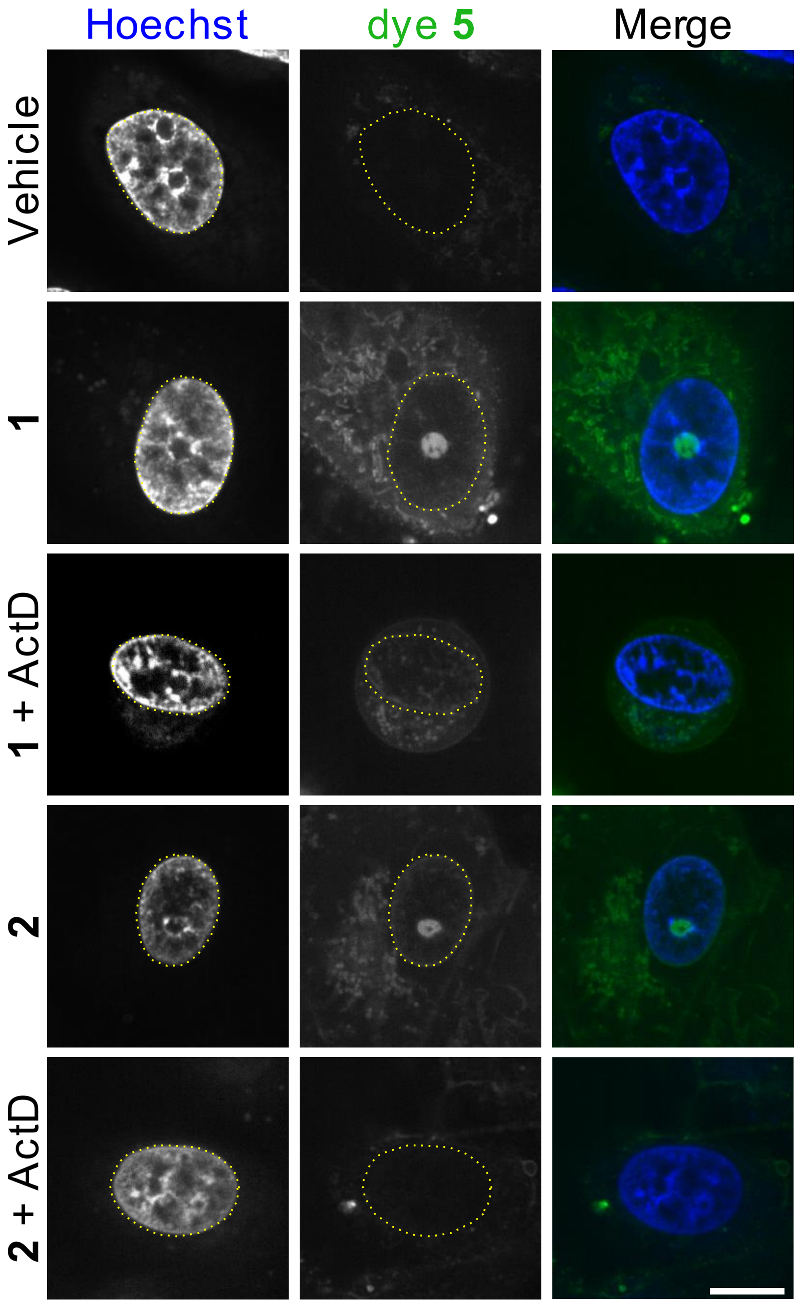

Figure S10: Detection of nascent RNA using 2TCOa- and BCN-Tri*PPP*ro nucleotides. Vero cells were washed with VP-SFM and incubated with 5 µM of the indicated Tri*PPP*ro nucleotide, with or without the transcription inhibitor actinomycin D (ActD). After labeling, cells were washed three times with VP-SFM, and ActD was reapplied where indicated. After 30 min incubation with 5 µM tetrazine-fluorophore conjugate (**5**) to visualize Tri*PPP*ro-tagged RNA (green), and Hoechst (blue) to label nuclei, live-cell imaging was performed using a Nikon Ti2 spinning disk fluorescence microscope equipped with a 100× Apo TIRF objective. Dye **5** was excited with the 488 nm laser line (80% intensity, 600 ms exposure time), and Hoechst with the 405 nm laser line (50% intensity, 200 ms exposure time). Nuclear outlines are circled (yellow). Individual channels are shown in greyscale, the merged image is shown in color. Scalebar, 10 µm. Representative images of at least 3 biological replicates.

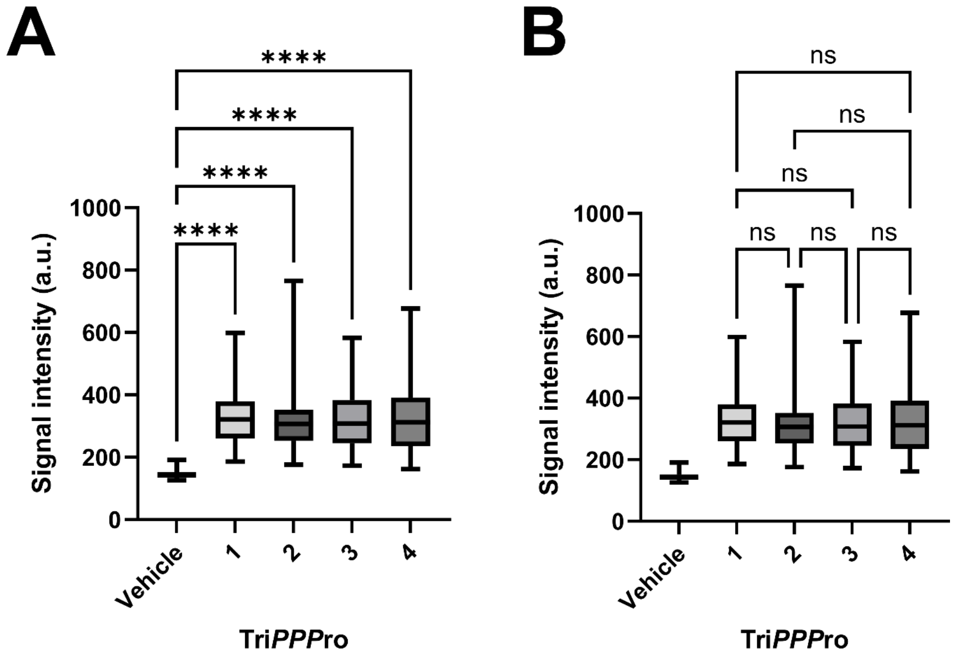

Figure S11: Representative nucleolar signal intensity upon Tri*PPP*ro treatment. (**A**) Comparison of nucleolar signal intensity between vehicle-treated and Tri*PPP*ro-treated cells. (**B**) Comparison of nucleolar signal intensity in cells treated with different Tri*PPP*ro variants. Signal intensities were measured in at least 100 nucleoli per condition. Boxplots represent the median (line), interquartile range (box), and the full range (whiskers). Statistical significance was assessed using the Kruskal–Wallis test followed by Dunn's multiple comparisons test. Significance notation: ns = not significant, $p < 0.05$ (*), $p < 0.01$ (**), $p < 0.001$ (***), $p < 0.0001$ (****).

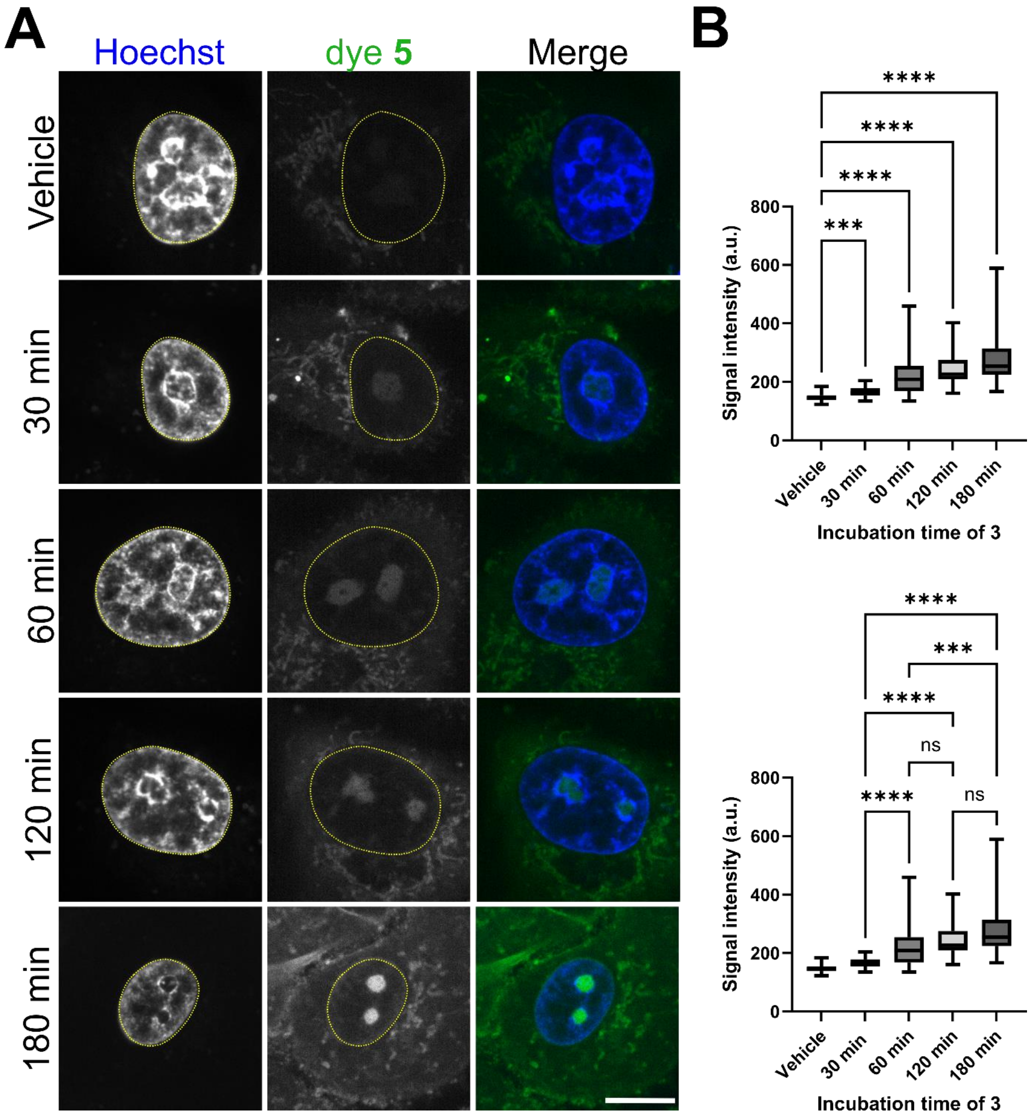

Figure S12: RNA-labeling of Vero cells with 3 using different incubation times with the Tri*PPP*ro. (**A**) Vero cells were washed with VP-SFM and incubated with 5 µM of **3** for the indicated time. After labeling, cells were washed three times with VP-SFM. After 30 min incubation with 5 µM tetrazine-fluorophore conjugate (**5**) to visualize Tri*PPP*ro-tagged RNA (green), and Hoechst (blue) to label nuclei, live-cell imaging was performed using a Nikon Ti2 spinning disk fluorescence microscope equipped with a 100× Apo TIRF objective. Nuclear outlines are circled (yellow). Individual channels are shown in greyscale, the merged image is shown in color. Scalebar, 10 µm. (**B**) Top: Comparison of nucleolar signal intensity between vehicle-treated and Tri*PPP*ro-treated cells. Bottom: Comparison of nucleolar signal intensity in cells treated with different Tri*PPP*ro incubation times. Signal intensities were measured in at least 60 nucleoli per condition. Boxplots represent the median (line), interquartile range (box), and the full range (whiskers). Statistical significance was assessed using the Kruskal–Wallis test followed by Dunn's multiple comparisons test. Significance notation: ns = not significant, $p < 0.05$ (*), $p < 0.01$ (**), $p < 0.001$ (***), $p < 0.0001$ (****).

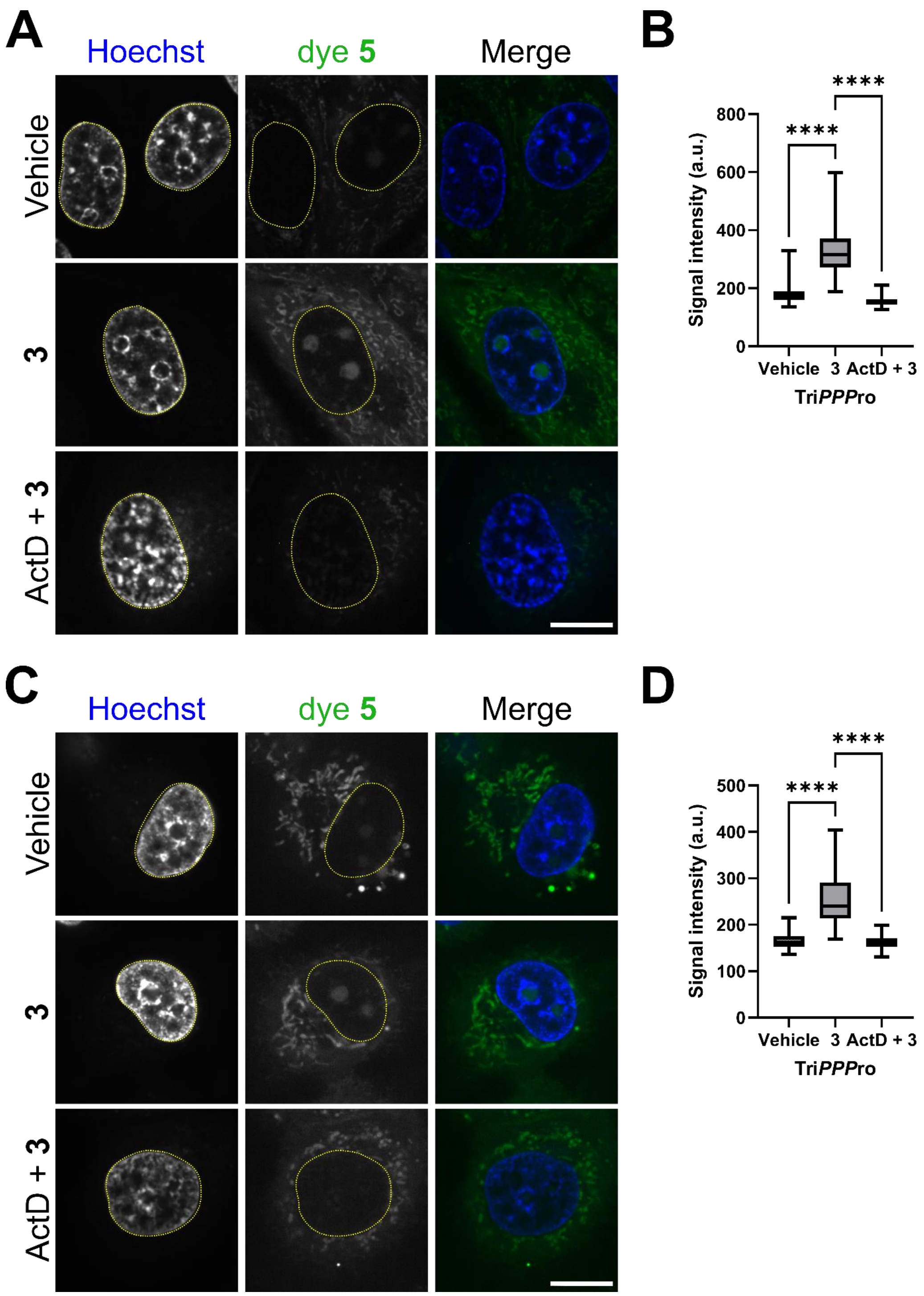

Figure S13: Detection of nascent RNA using BCN_CTP nucleotides in PK15 and A549 cells. (**A**) PK15 cells were washed with VP-SFM and incubated with 5 µM of **3**, with or without the transcription inhibitor actinomycin D (ActD). After labeling, cells were washed three times with VP-SFM, and ActD was reapplied where indicated. After 30 min incubation with 5 µM tetrazine-fluorophore conjugate (**5**) to visualize Tri*PPP*ro-tagged RNA (green), and Hoechst (blue) to label nuclei, live-cell imaging was performed using a Nikon Ti2 spinning disk fluorescence microscope equipped with a 100× Apo TIRF objective. Dye **5** was excited with the 488 nm laser line (40% intensity, 600 ms exposure time), and Hoechst with the 405 nm laser line (50% intensity, 200 ms exposure time). Nuclear outlines are circled (yellow). Individual channels are shown in greyscale, the merged image is shown in color. Scalebar, 10 µm. (**B**) Signal intensities were measured in at least 90 nucleoli per condition. Boxplots represent the median (line), interquartile range (box), and the full range (whiskers). Statistical significance was assessed using the Kruskal–Wallis test followed by Dunn's multiple comparisons test. Significance notation: ns = not significant, $p < 0.05$ (*), $p < 0.01$ (**), $p < 0.001$ (***), $p < 0.0001$ (****). (**C**) A549 cells were treated accordingly to (**A**). (**D**) Quantification performed accordingly to (**B**).

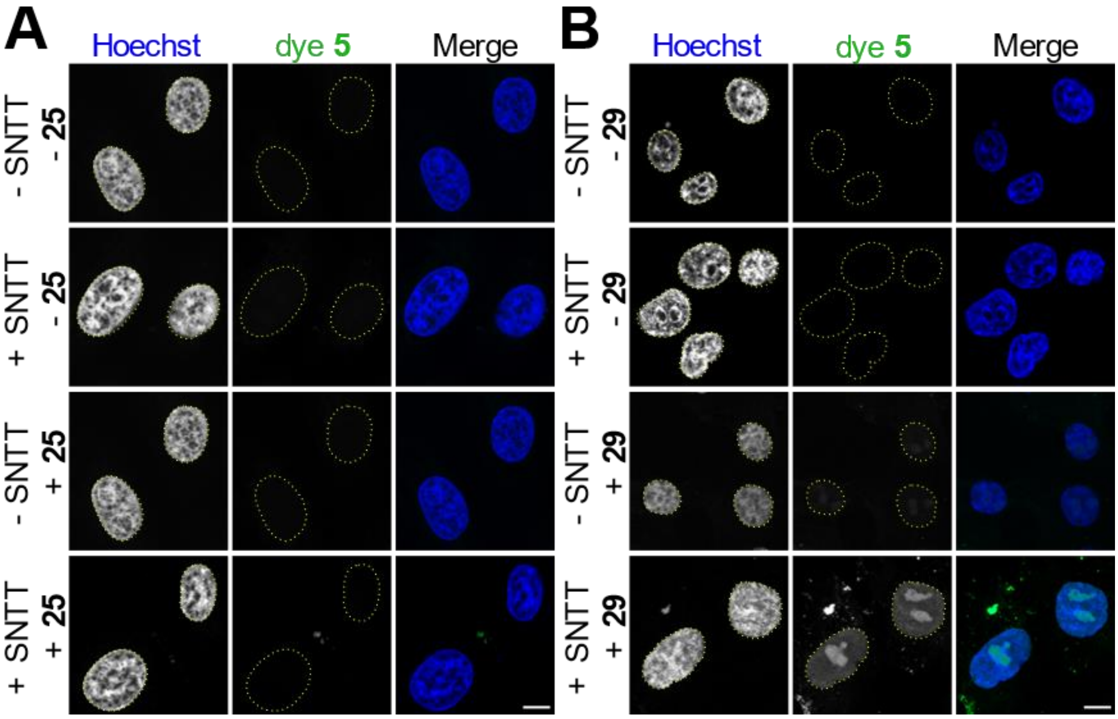

Figure S14: SNTT-based labeling of RNA in Vero cells. (**A**) Vero cells were incubated with nucleotide **25** and **SNTT** (60 µM) in tricine buffer for 10 min, then incubated in DMEM for 4 h. Following incubation, the cells were fixed, permeabilized, and incubated with 5 µM **5** overnight. (**B**) Vero cells were treated with nucleotide **29** and **SNTT** (35 µM) in tricine buffer for 10 min, then incubated in DMEM for 4 hours. Following incubation, the cells were fixed, permeabilized, and thoroughly washed with PBS to remove excessive fluorescent background. Imaging was performed in fixed cells. RNA labeled with **25** or **29** is visualized in green, and nucleus staining with Hoechst in blue. Nuclear outlines are circled (yellow). Individual channels are shown in greyscale; the merged image is shown in color. Scalebar, 10 µm. Representative images of at least 2 biological replicates.

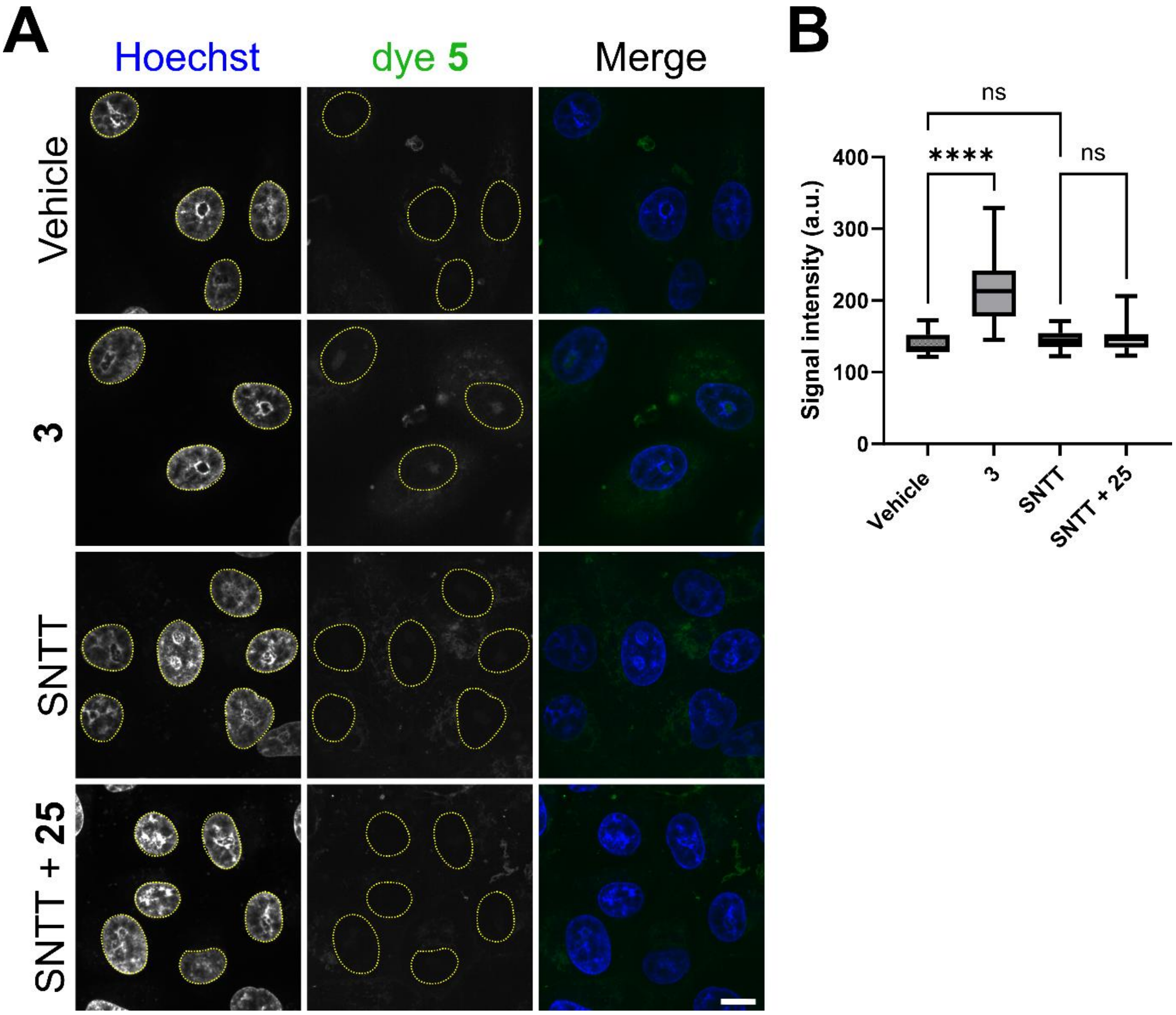

Figure S15: Tri*PPP*ro vs SNTT-based RNA labelling in Vero cells. (**A**) Vero cells were incubated with either 5 µM Tri*PPP*ro **3** in VP-SFM or nucleotide **25** and **SNTT** (5 µM) in tricine buffer for 1 h. After labeling, the cells were washed three times with VP-SFM. After 30 min incubation with 5 µM tetrazine-fluorophore conjugate (**5**) to visualize Tri*PPP*ro-tagged RNA (green), and Hoechst (blue) to label nuclei, live-cell imaging was performed using a Nikon Ti2 spinning disk fluorescence microscope equipped with a 100× Apo TIRF objective. Nuclear outlines are circled (yellow). Individual channels are shown in greyscale; the merged image is shown in color. Scalebar, 10 µm. (**B**) Signal intensities were measured in at least 50 nucleoli per condition. Boxplots represent the median (line), interquartile range (box), and the full range (whiskers). Statistical significance was assessed using the Kruskal–Wallis test followed by Dunn's multiple comparisons test. Significance notation: ns = not significant, $p < 0.05$ (*), $p < 0.01$ (**), $p < 0.001$ (***), $p < 0.0001$ (****).

5. Spectra

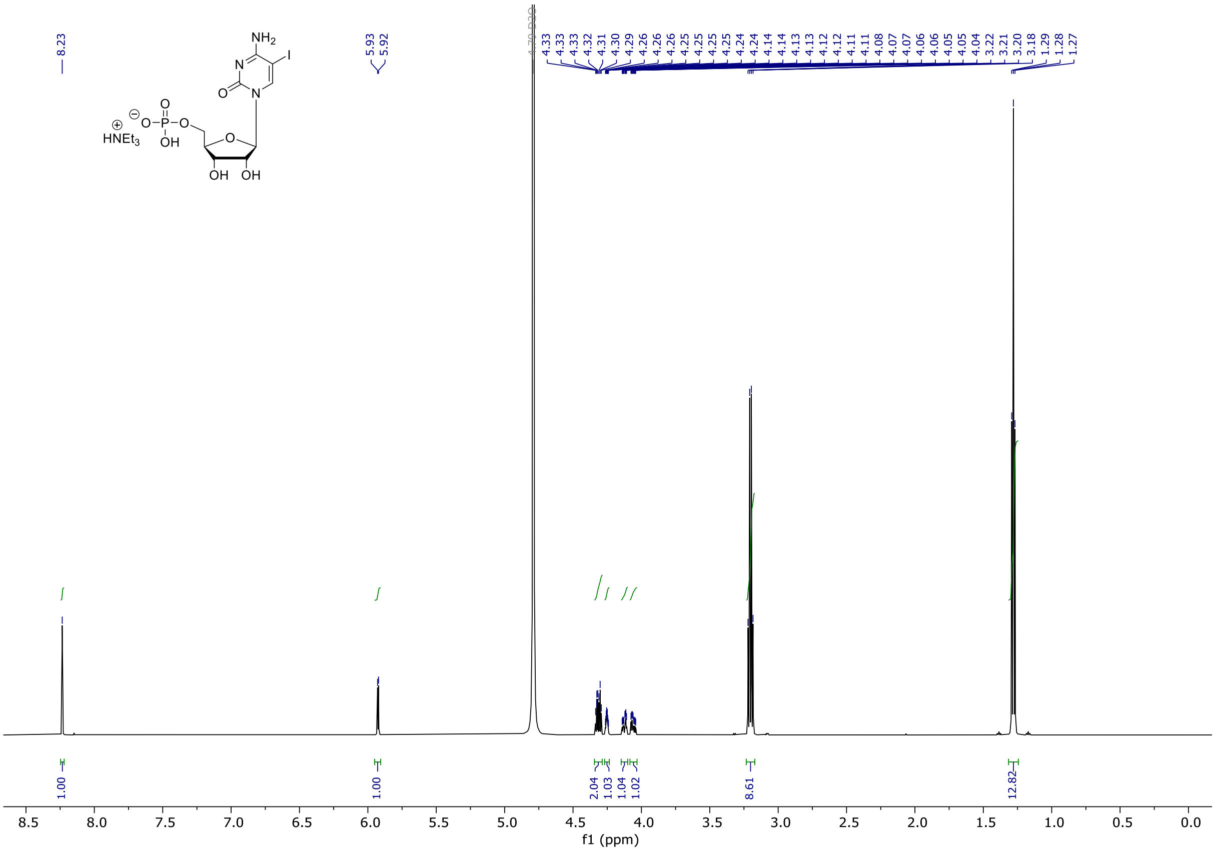

8.23
5.93
5.92
4.33 4.33 4.32 4.31 4.30 4.29 4.26 4.26 4.25 4.25 4.25 4.25 4.24 4.24 4.14 4.14 4.13 4.13 4.12 4.12 4.11 4.11 4.08 4.07 4.07 4.06 4.06 4.05 4.05 4.04 3.22 3.21 3.20 3.18 1.29 1.28 1.27
1.00
1.00
2.04
1.03
1.04
1.02
8.61
12.82
8.5 8.0 7.5 7.0 6.5 6.0 5.5 5.0 4.5 4.0 3.5 3.0 2.5 2.0 1.5 1.0 0.5 0.0
f1 (ppm)

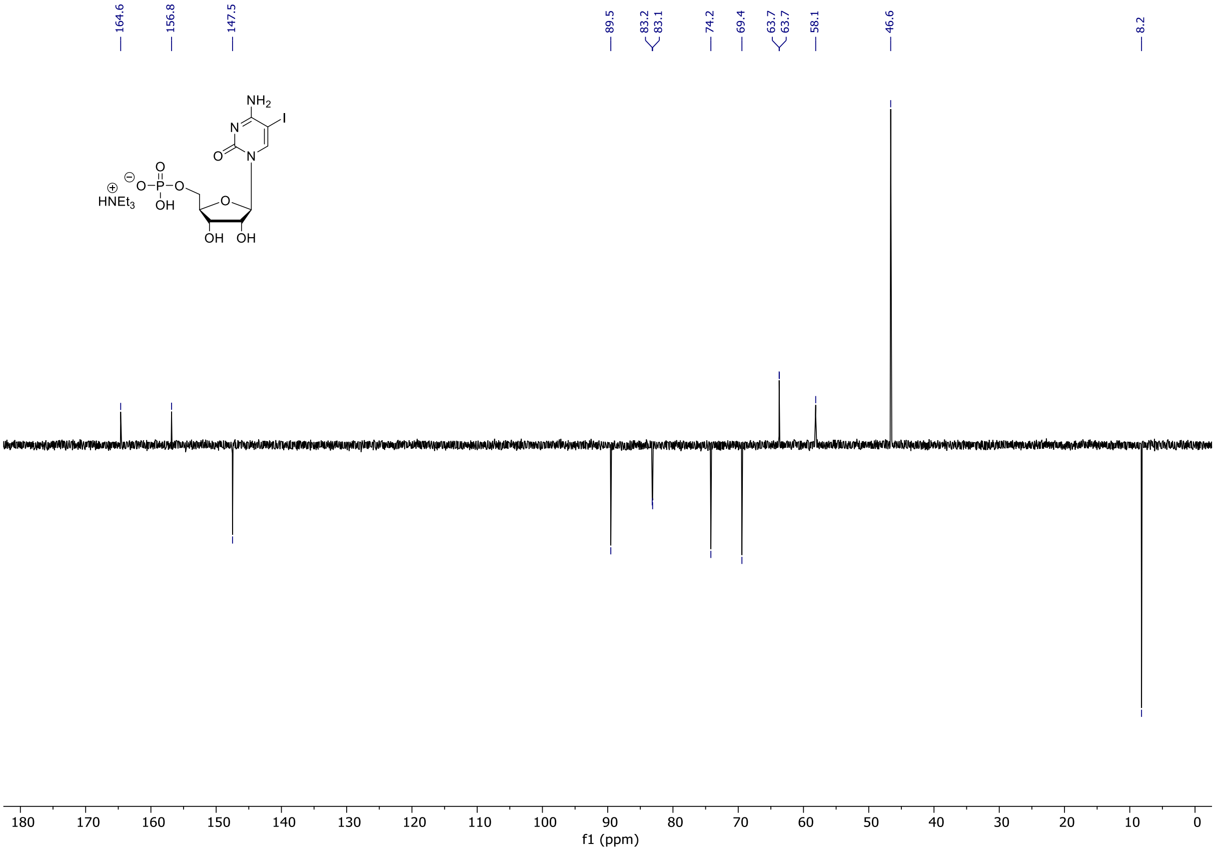

164.6
156.8
147.5
89.5
83.2
83.1
74.2
69.4
63.7
63.7
58.1
46.6
8.2
180 170 160 150 140 130 120 110 100 90 80 70 60 50 40 30 20 10 0
f1 (ppm)

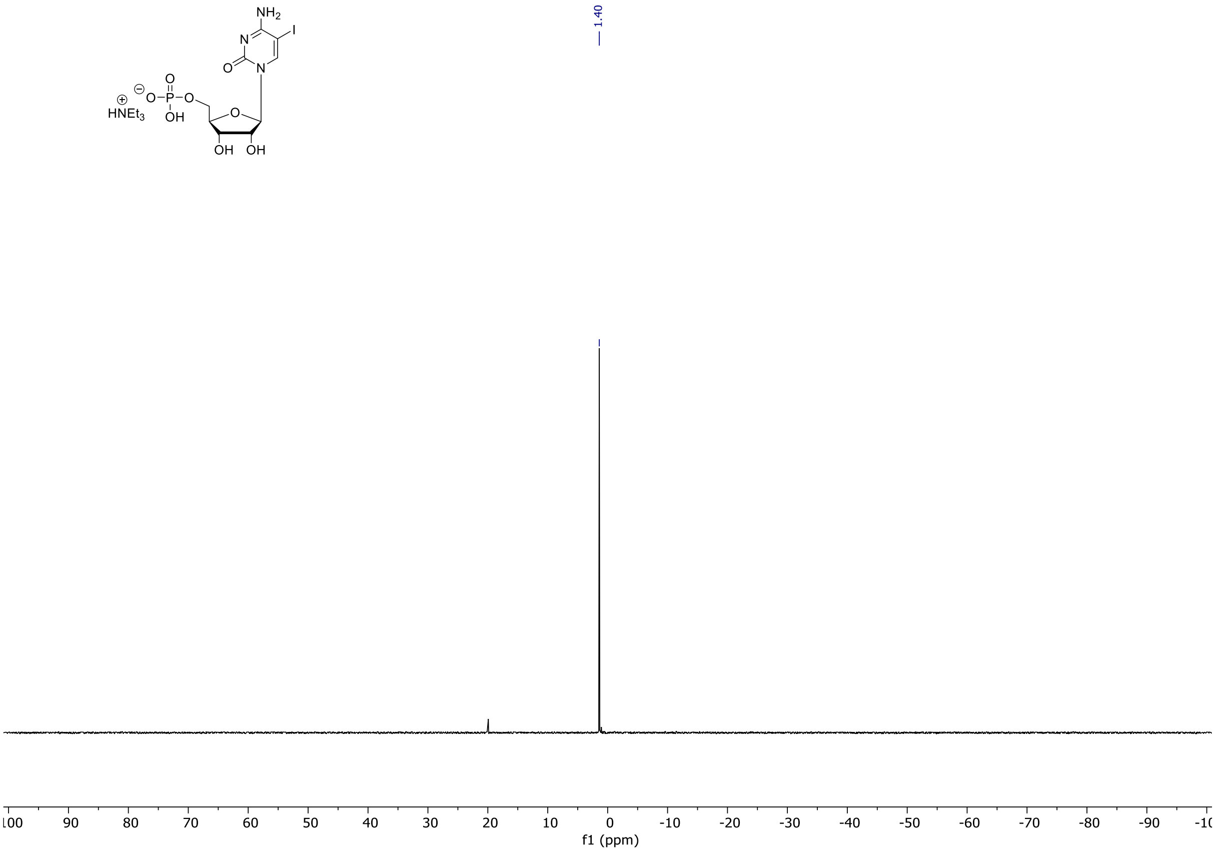

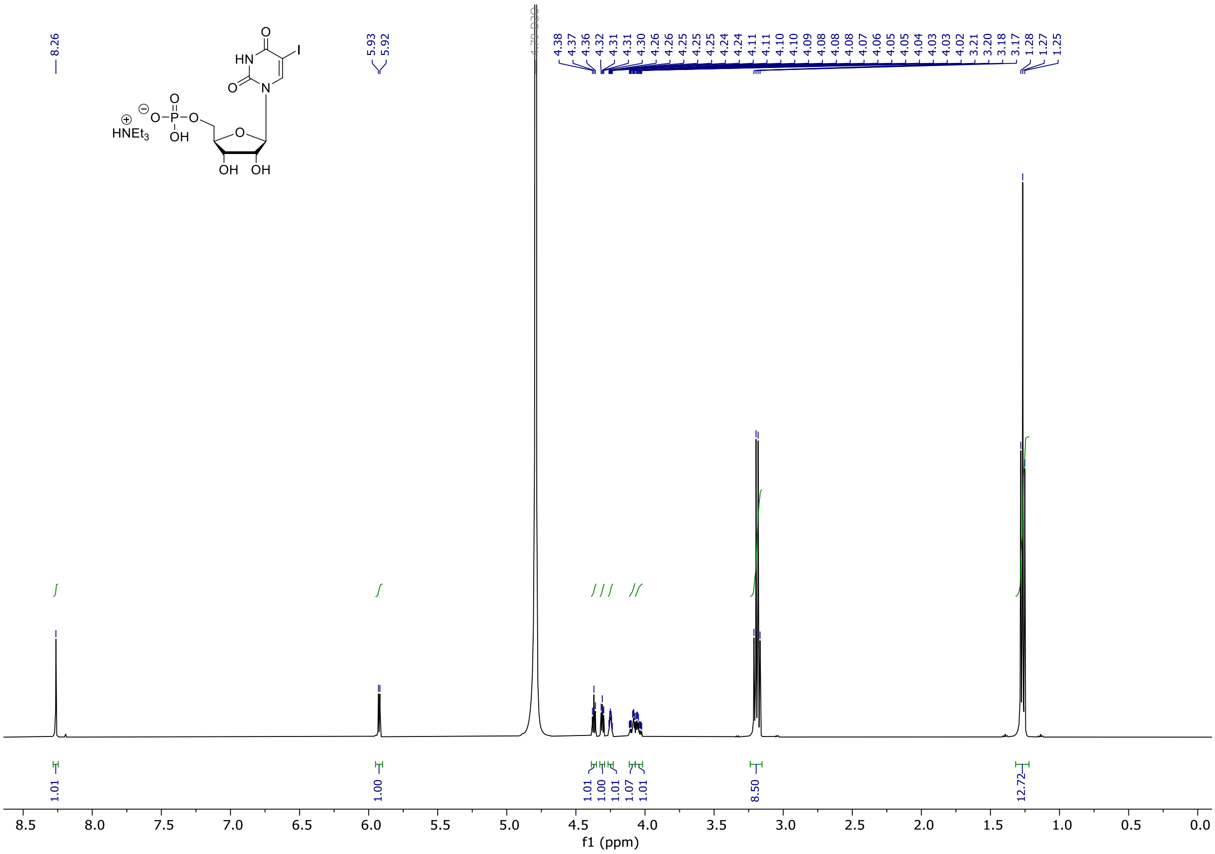

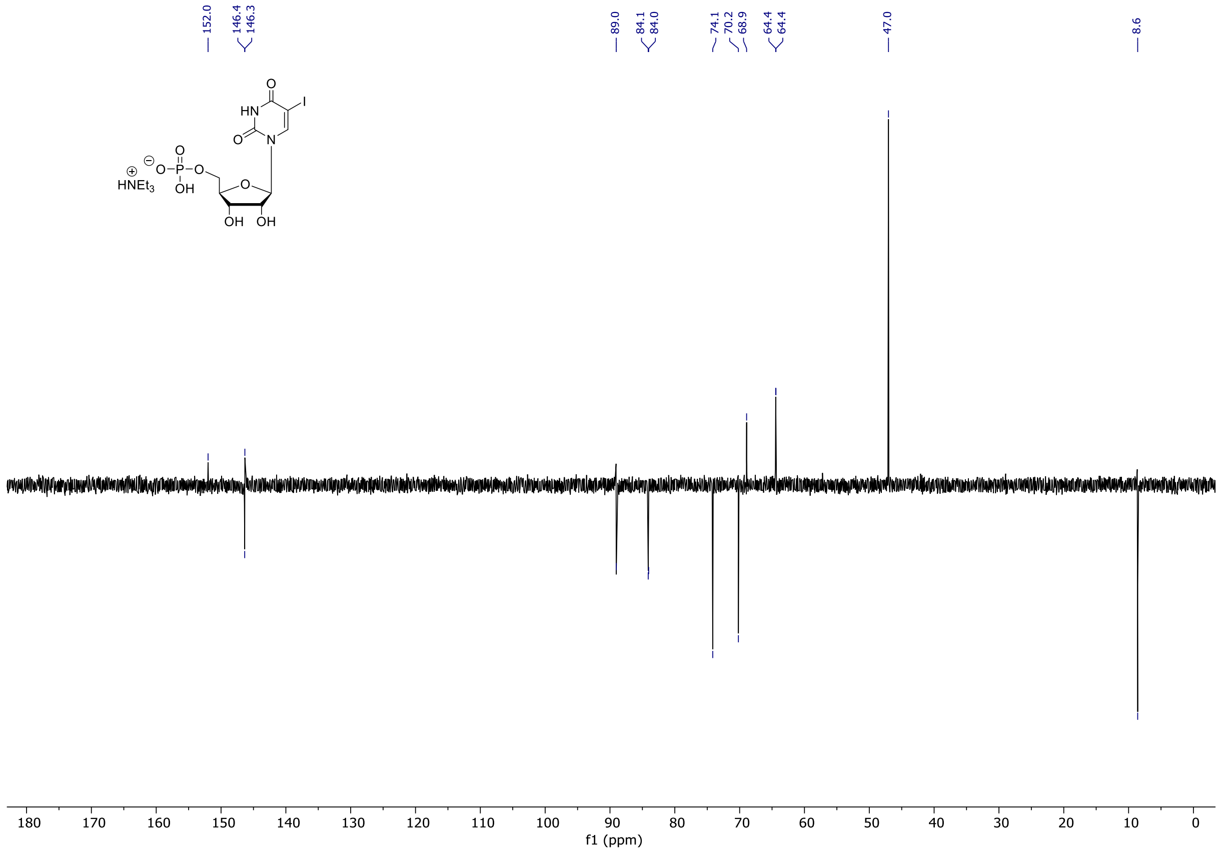
152.0
146.4
146.3
89.0
84.1
84.0
74.1
70.2
68.9
64.4
64.4
47.0
8.6
O
HN
I
O
N
O
O
P
O
O
HNEt3
OH
OH
OH
180 170 160 150 140 130 120 110 100 90 80 70 60 50 40 30 20 10 0
f1 (ppm)

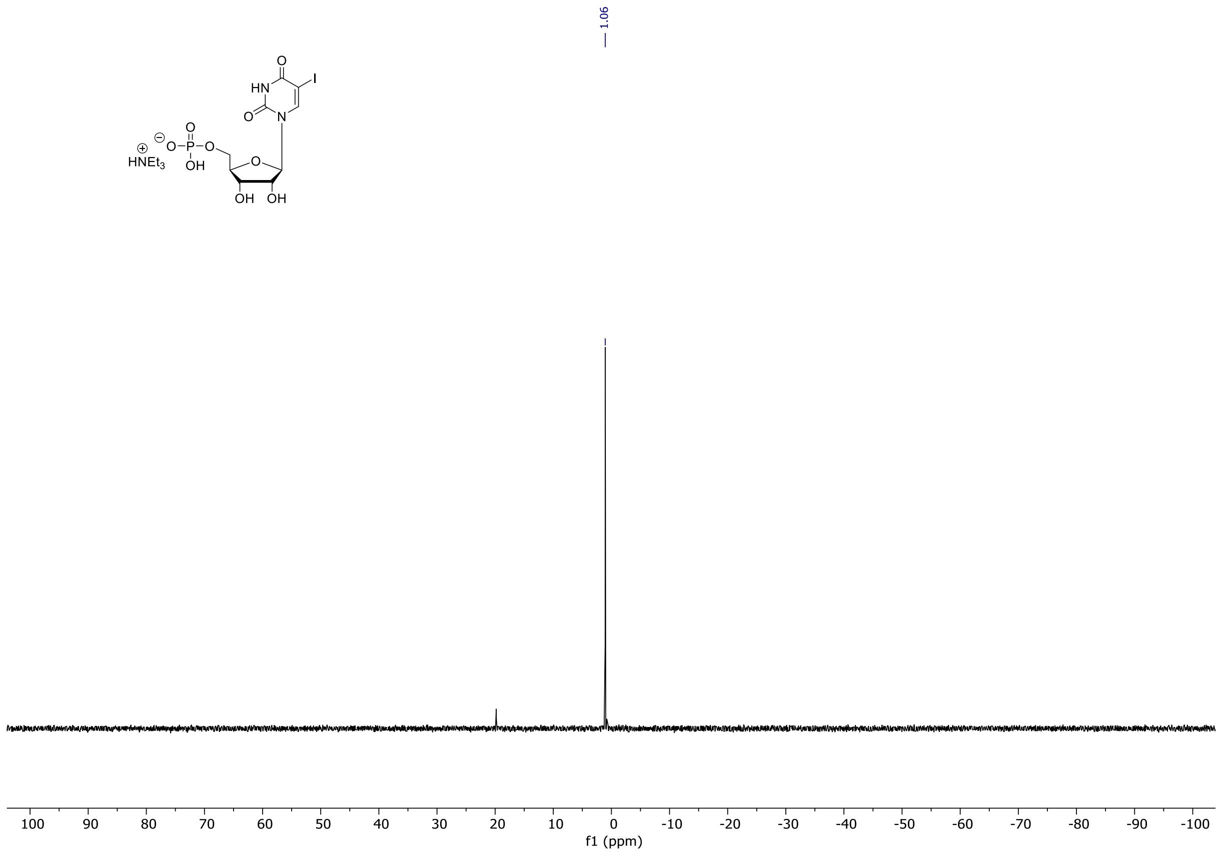
1.06
O
HN
I
O
N
O
O
P
O
O
HNEt3
OH
OH
OH
100 90 80 70 60 50 40 30 20 10 0 -10 -20 -30 -40 -50 -60 -70 -80 -90 -100
f1 (ppm)

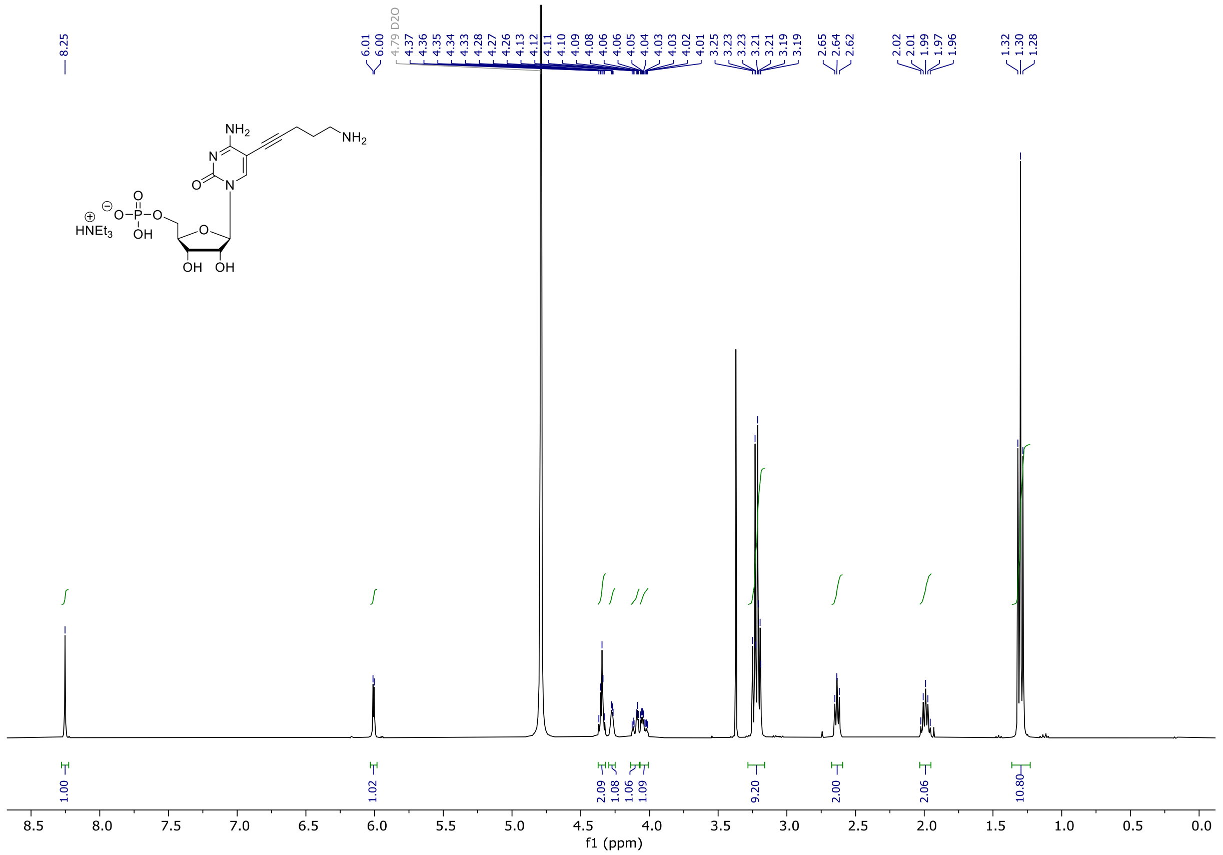

NH2
N
O
NH2
O
P
O
HNEt3
OH
OH OH
8.25
6.01
6.00
4.79 D2O
1.00
1.02
2.09
1.08
1.06
1.09
9.20
2.00
2.06
10.80
8.5 8.0 7.5 7.0 6.5 6.0 5.5 5.0 4.5 4.0 3.5 3.0 2.5 2.0 1.5 1.0 0.5 0.0
f1 (ppm)

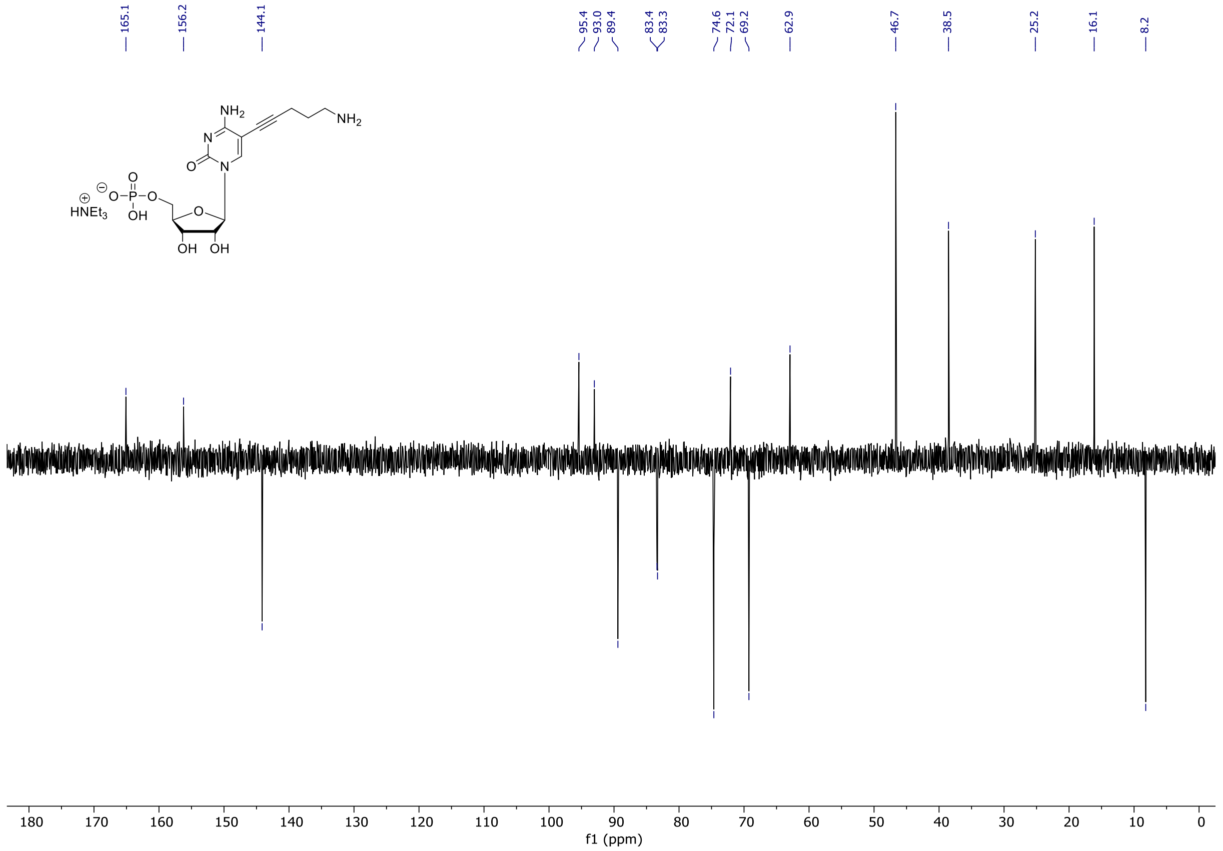

165.1
156.2
144.1
95.4
93.0
89.4
83.4
83.3
74.6
72.1
69.2
62.9
46.7
38.5
25.2
16.1
8.2
180 170 160 150 140 130 120 110 100 90 80 70 60 50 40 30 20 10 0
f1 (ppm)

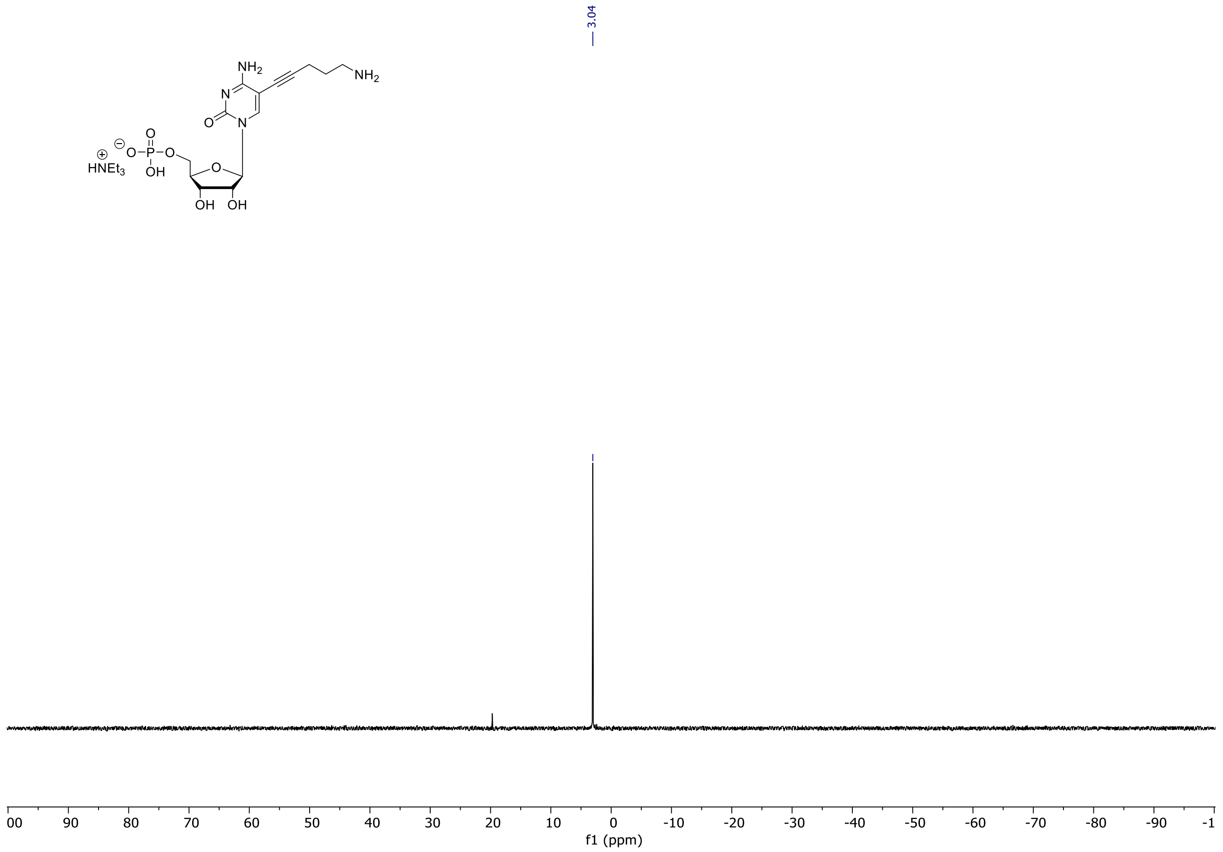

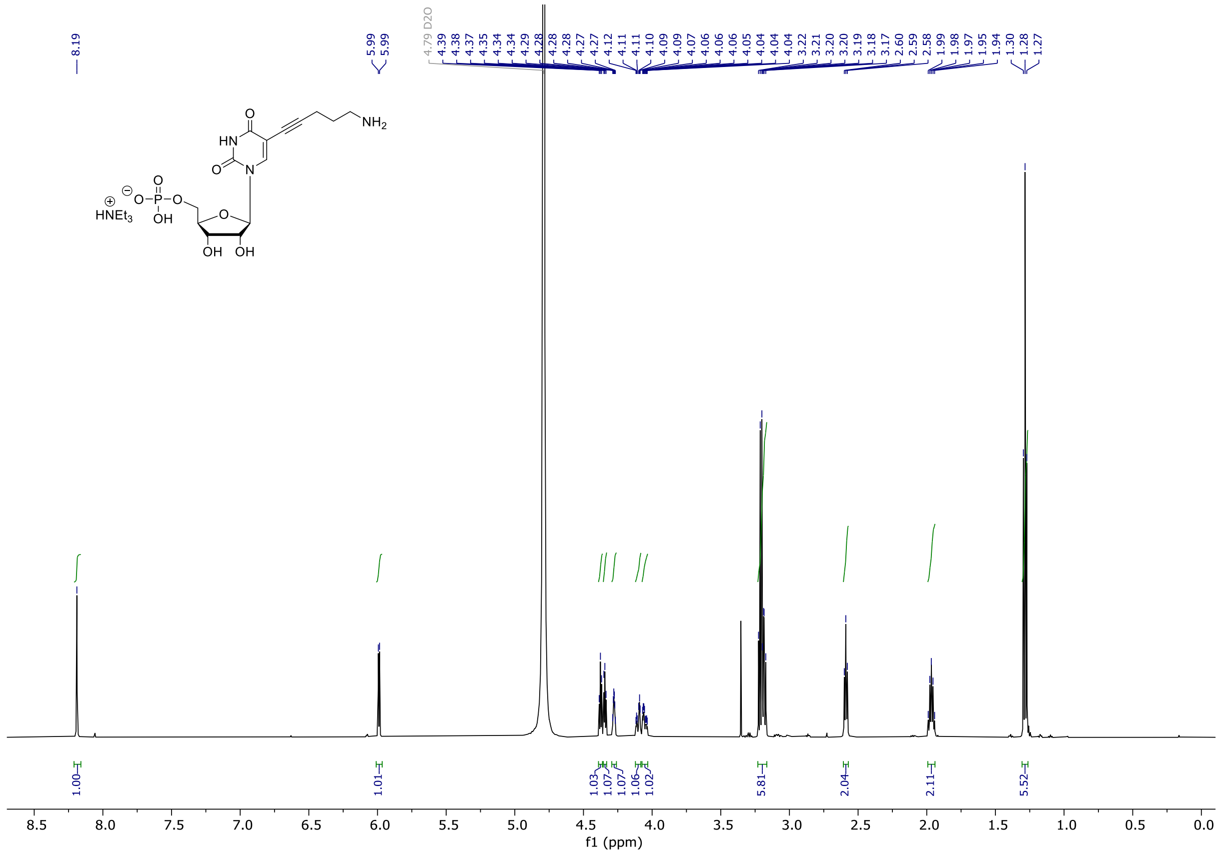

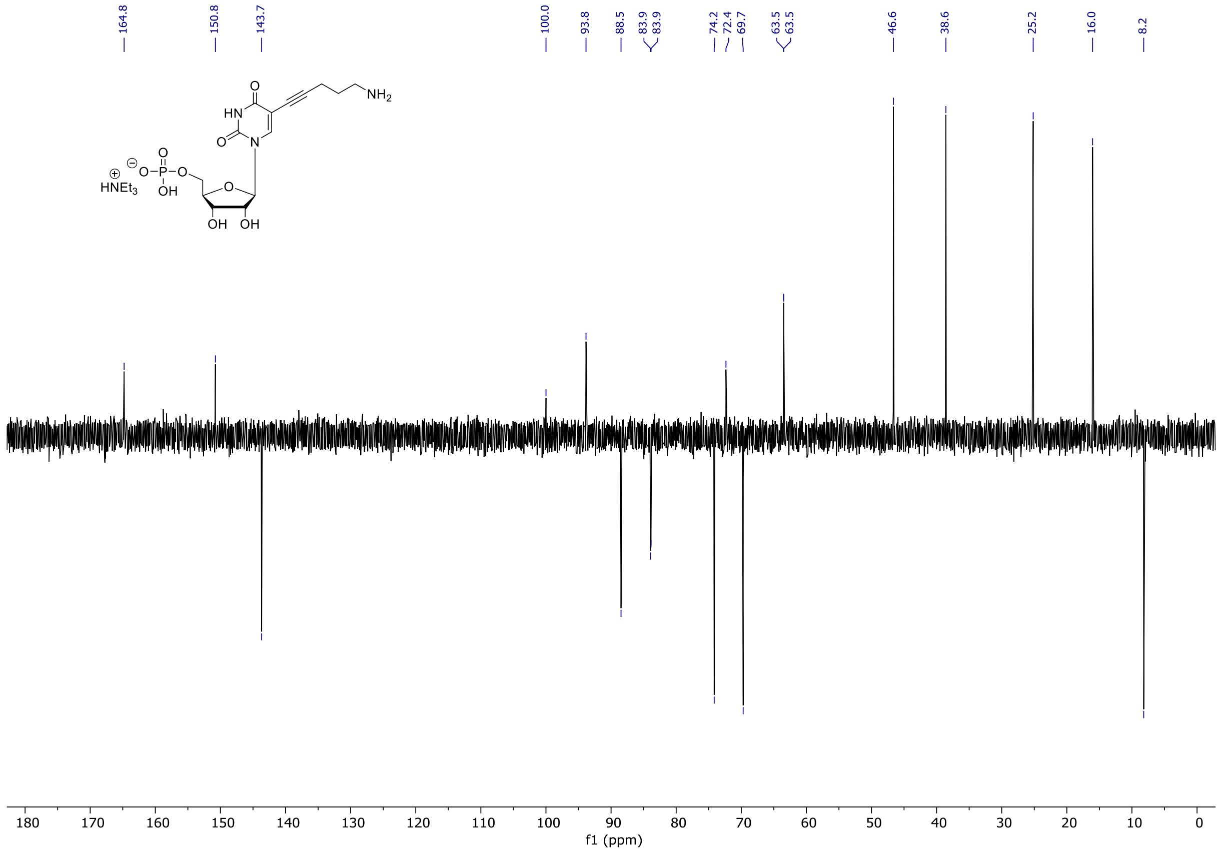
164.8
150.8
143.7
100.0
93.8
88.5
83.9
83.9
74.2
72.4
69.7
63.5
63.5
46.6
38.6
25.2
16.0
8.2
NH2
HN
O
N
O
O
O-P-O
OH
HNEt3
OH OH
f1 (ppm)

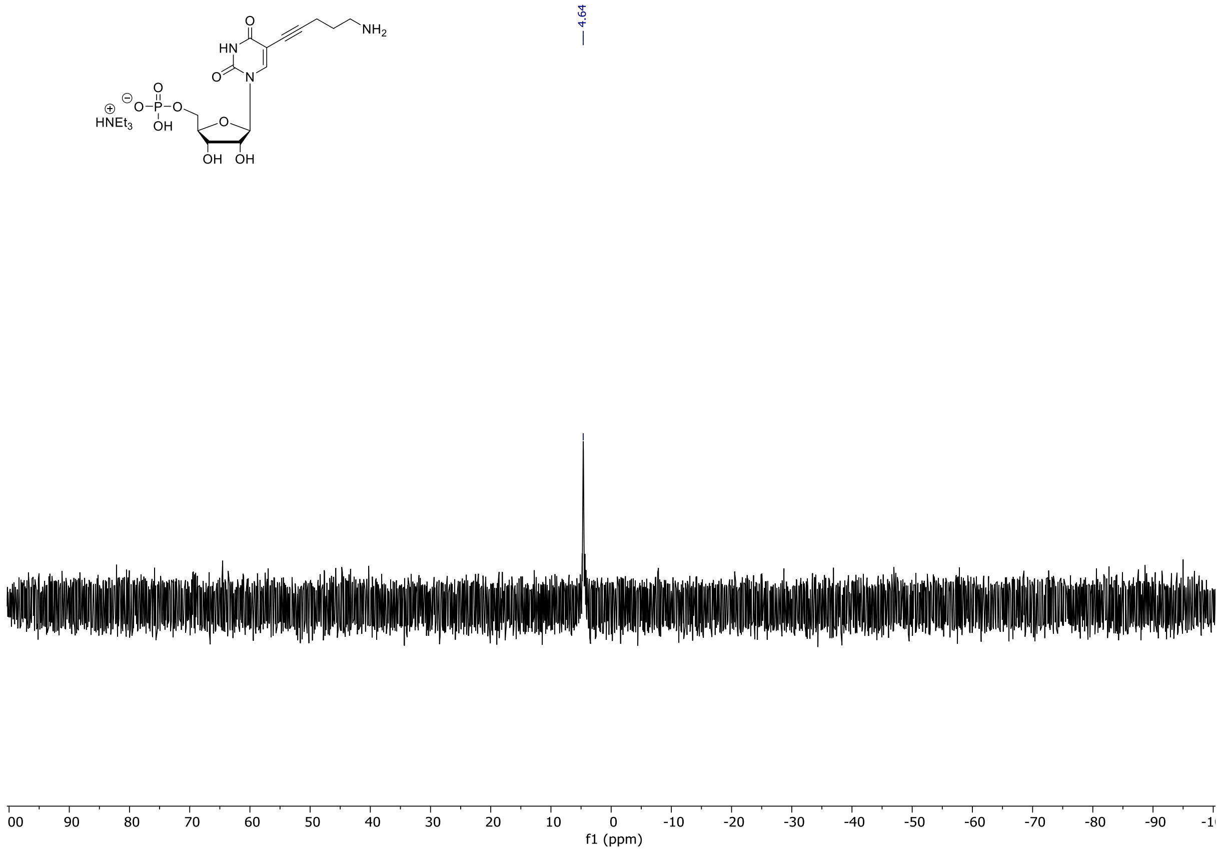
4.64
NH2
HN
O
N
O
O
O-P-O
OH
HNEt3
OH OH
f1 (ppm)

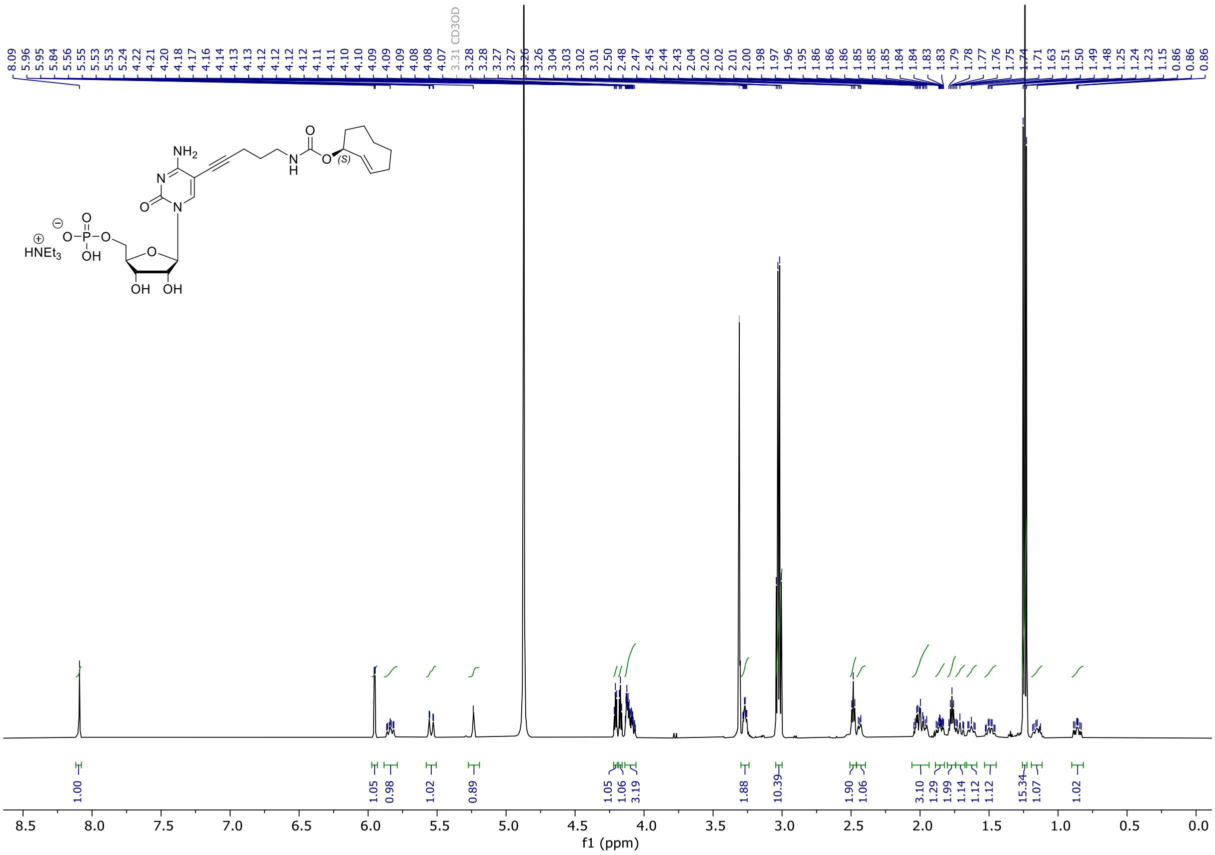

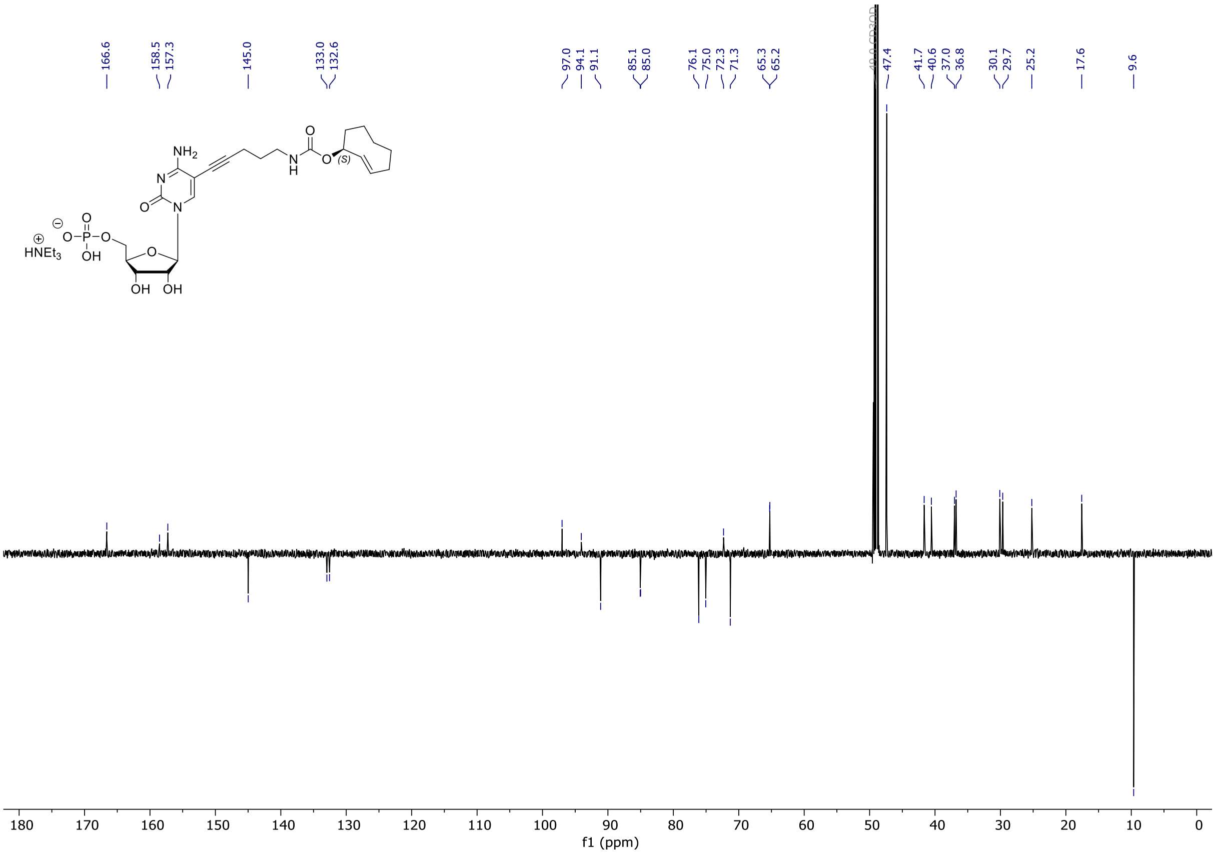

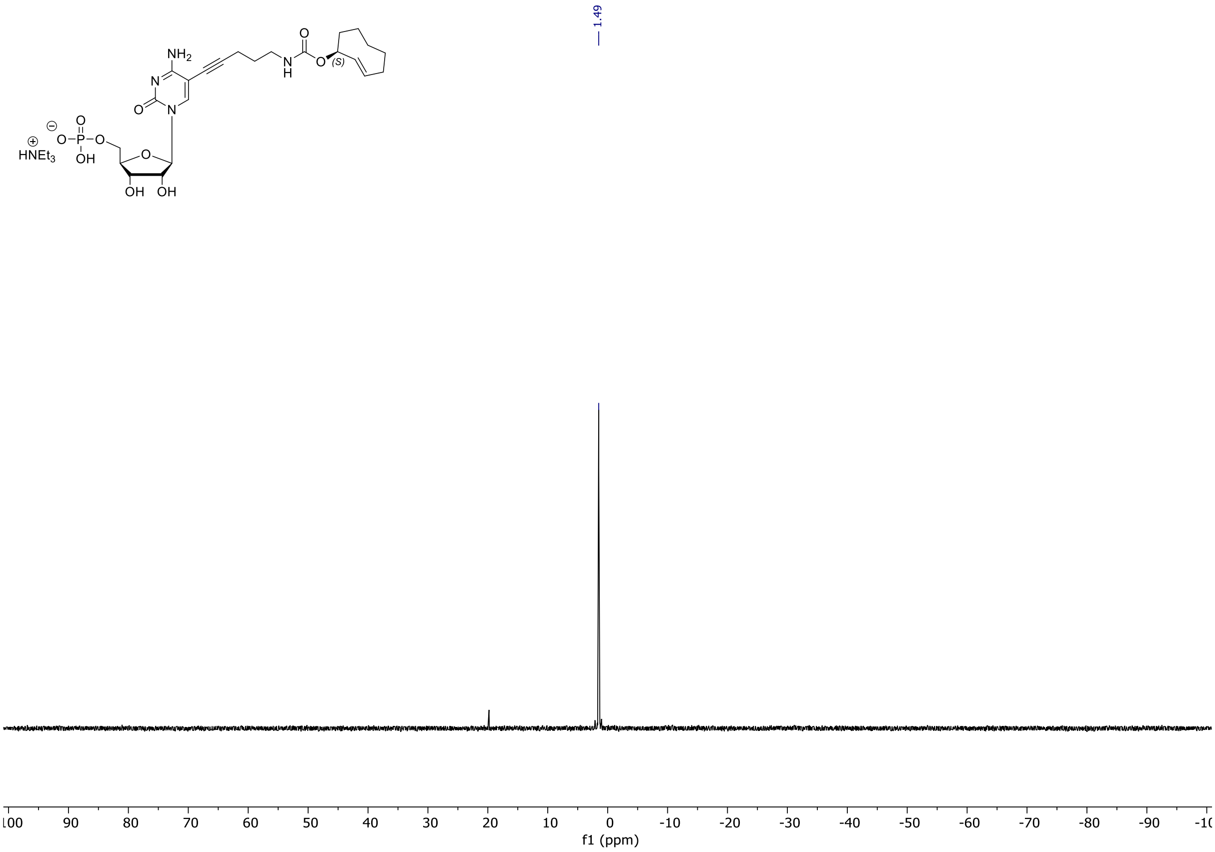

NH2
HNEt3
OH
OH OH
(S)
1.49
f1 (ppm)

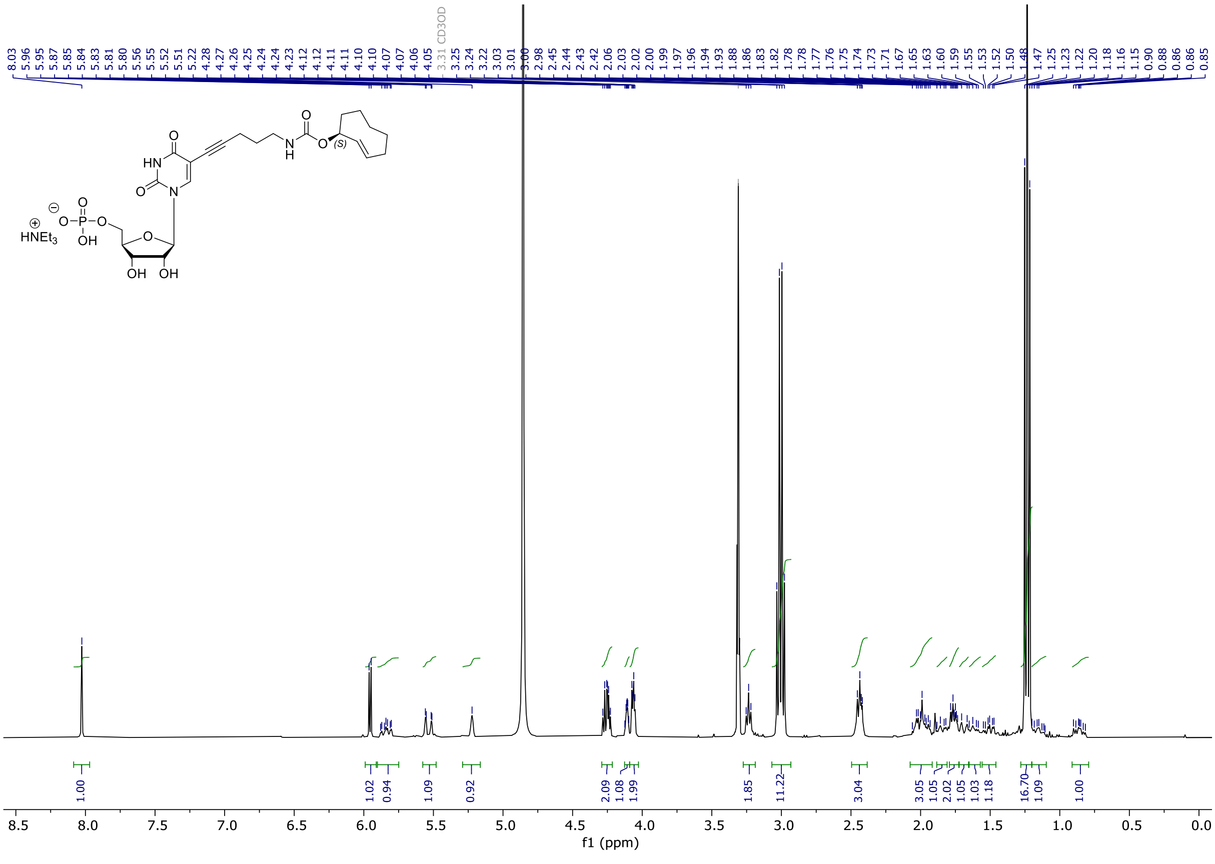

CD3OD
HN
HNEt3
OH
OH OH
(S)
f1 (ppm)

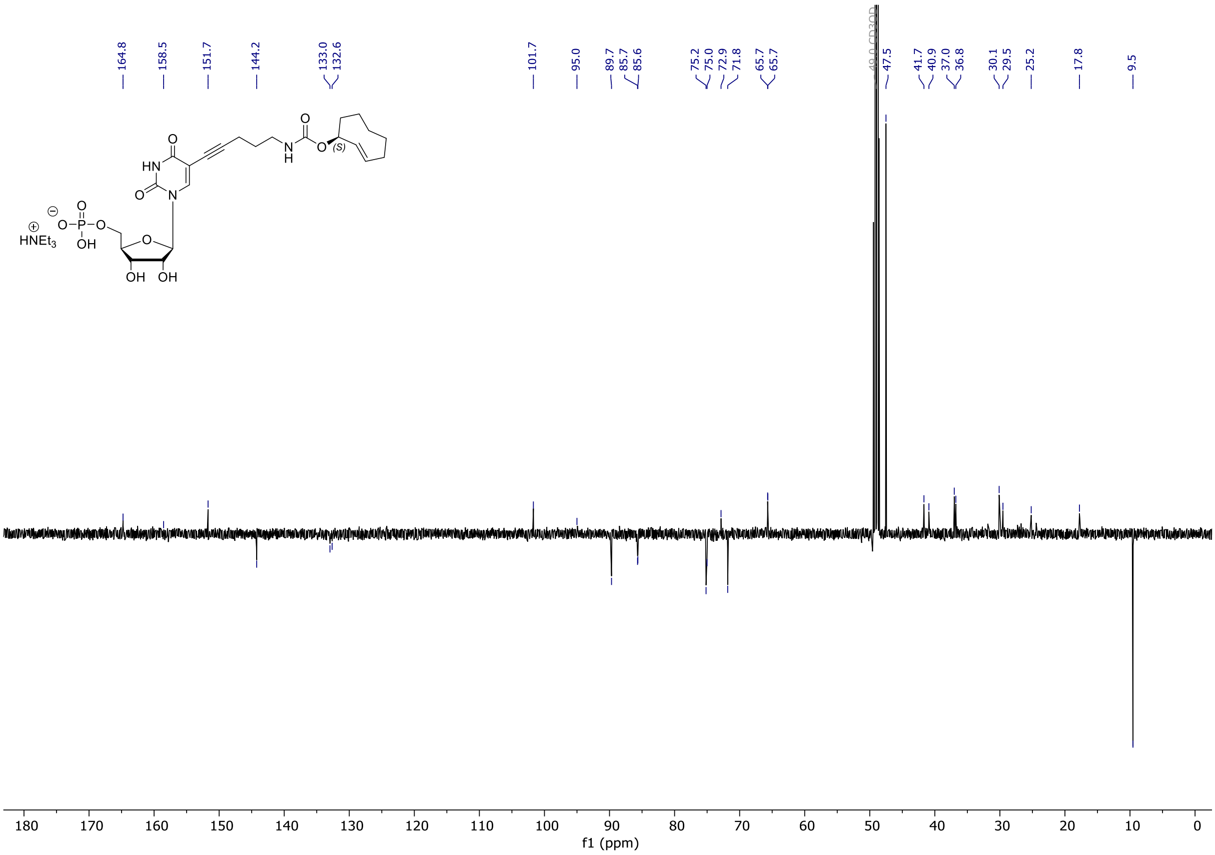
164.8
158.5
151.7
144.2
133.0
132.6
101.7
95.0
89.7
85.7
85.6
75.2
75.0
72.9
71.8
65.7
65.7
47.5
41.7
40.9
37.0
36.8
30.1
29.5
25.2
17.8
9.5
HN
O
N
H
(S)
HNEt3
O-P-O
OH
OH OH
180 170 160 150 140 130 120 110 100 90 80 70 60 50 40 30 20 10 0
f1 (ppm)

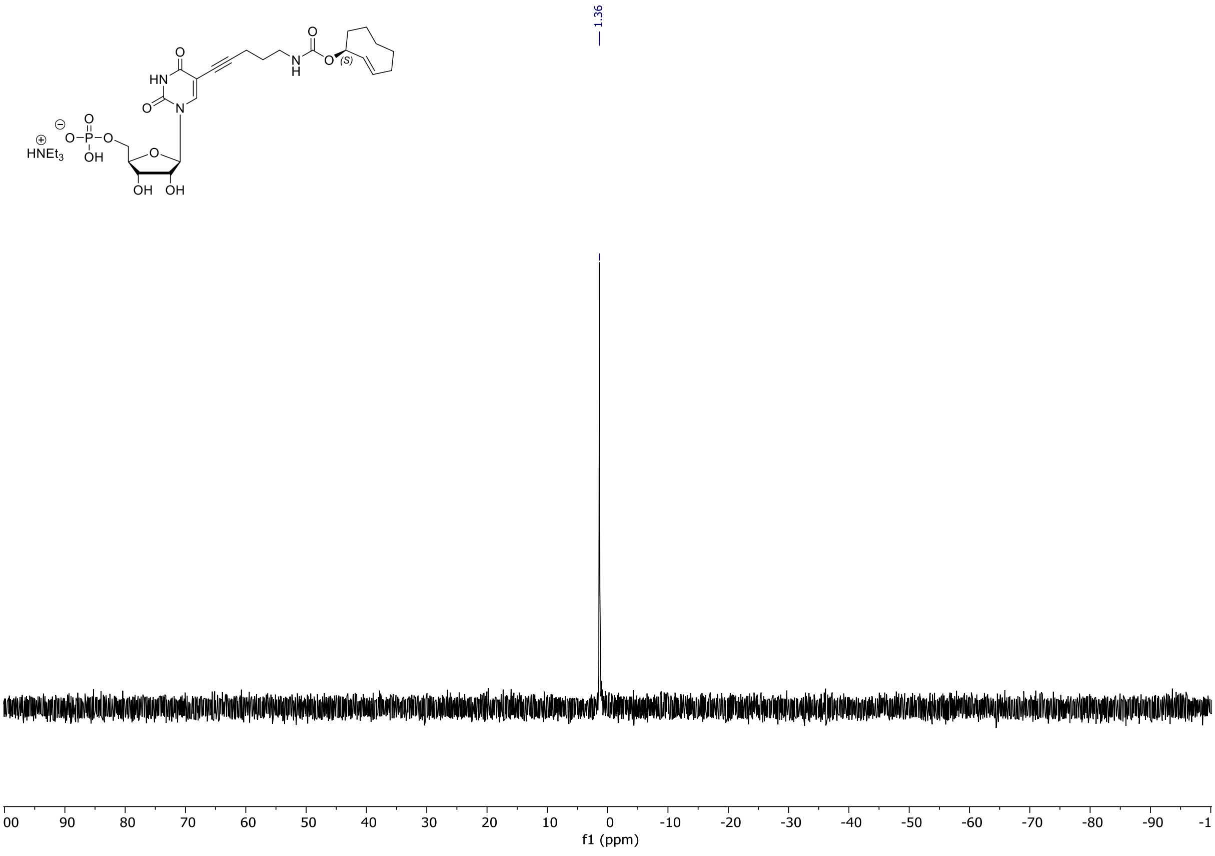
1.36
HN
O
N
H
(S)
HNEt3
O-P-O
OH
OH OH
00 90 80 70 60 50 40 30 20 10 0 -10 -20 -30 -40 -50 -60 -70 -80 -90 -1
f1 (ppm)

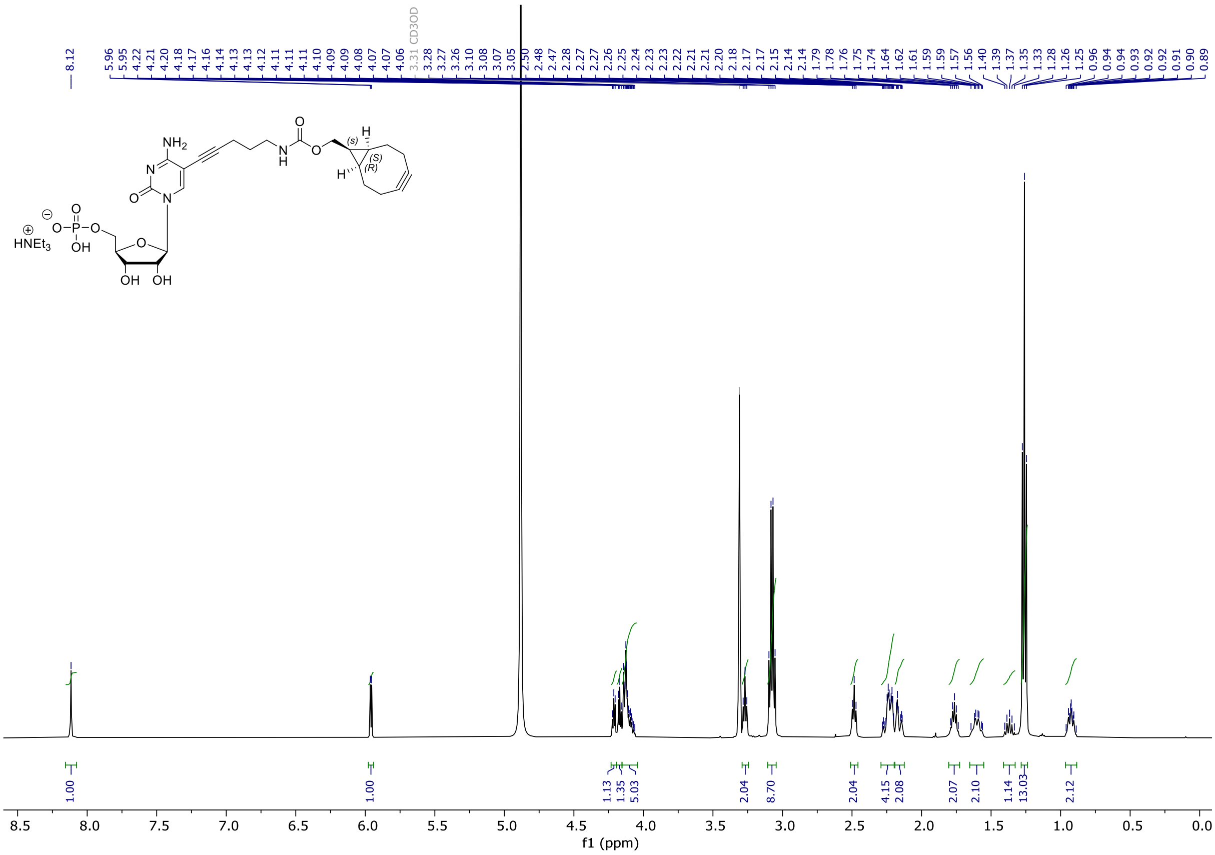
8.12
5.96 5.95 4.21 4.20 4.18 4.17 4.16 4.14 4.13 4.13 4.12 4.11 4.11 4.11 4.10 4.09 4.09 4.08 4.07 4.07 4.06
3.31 CD3OD
3.28 3.27 3.26 3.10 3.08 3.07 3.05 2.50 2.48 2.47 2.28 2.27 2.27 2.26 2.25 2.24 2.23 2.23 2.22 2.21 2.21 2.20 2.18 2.17 2.17 2.15 2.14 2.14 1.79 1.78 1.76 1.75 1.74 1.64 1.62 1.61 1.59 1.59 1.57 1.56 1.40 1.39 1.37 1.35 1.33 1.28 1.26 1.25 0.96 0.94 0.94 0.93 0.92 0.91 0.90 0.89
1.00
1.00
1.13 1.35 5.03
2.04 8.70
2.04 4.15 2.08
2.07 2.10
1.14 13.03
2.12
f1 (ppm)

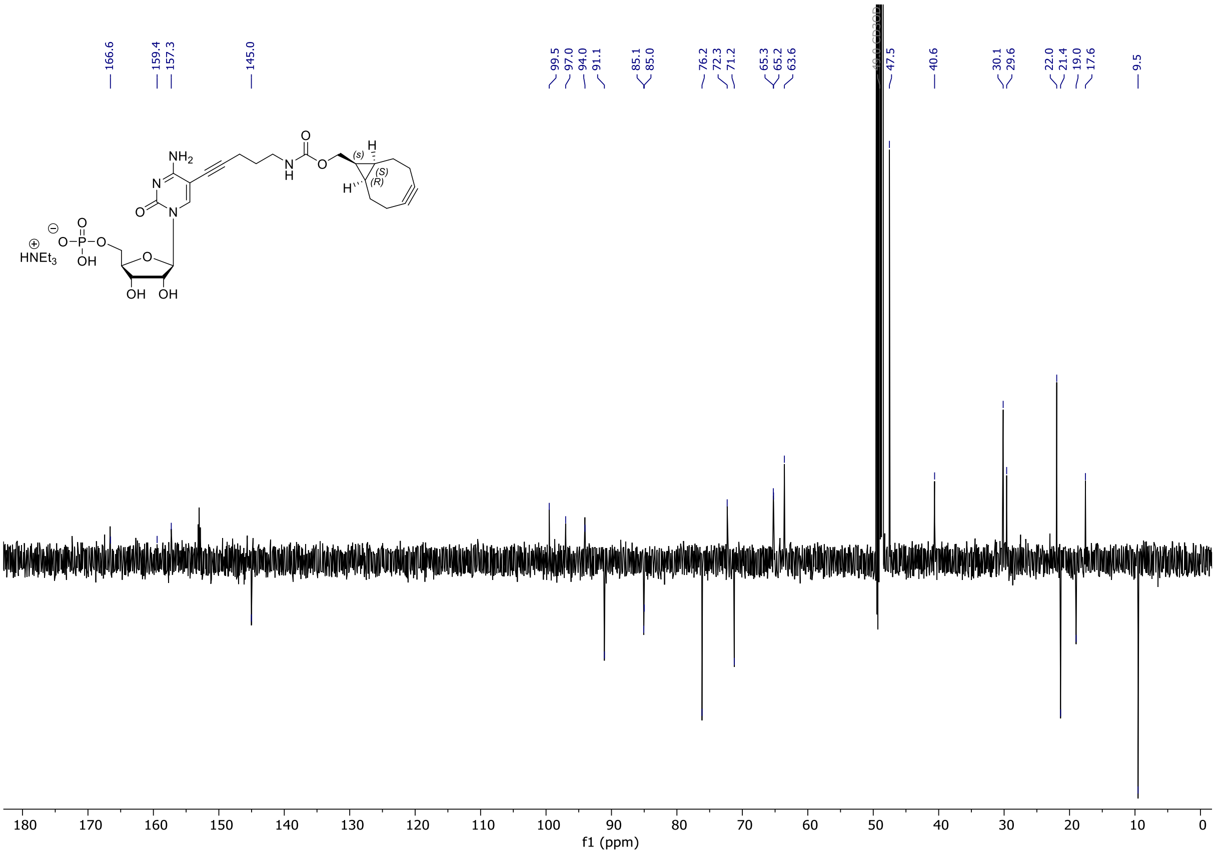
166.6
159.4 157.3
145.0
99.5 97.0 94.0 91.1
85.1 85.0
76.2 72.3 71.2
65.3 65.2 63.6
47.5
40.6
30.1 29.6
22.0 21.4 19.0 17.6
9.5
f1 (ppm)

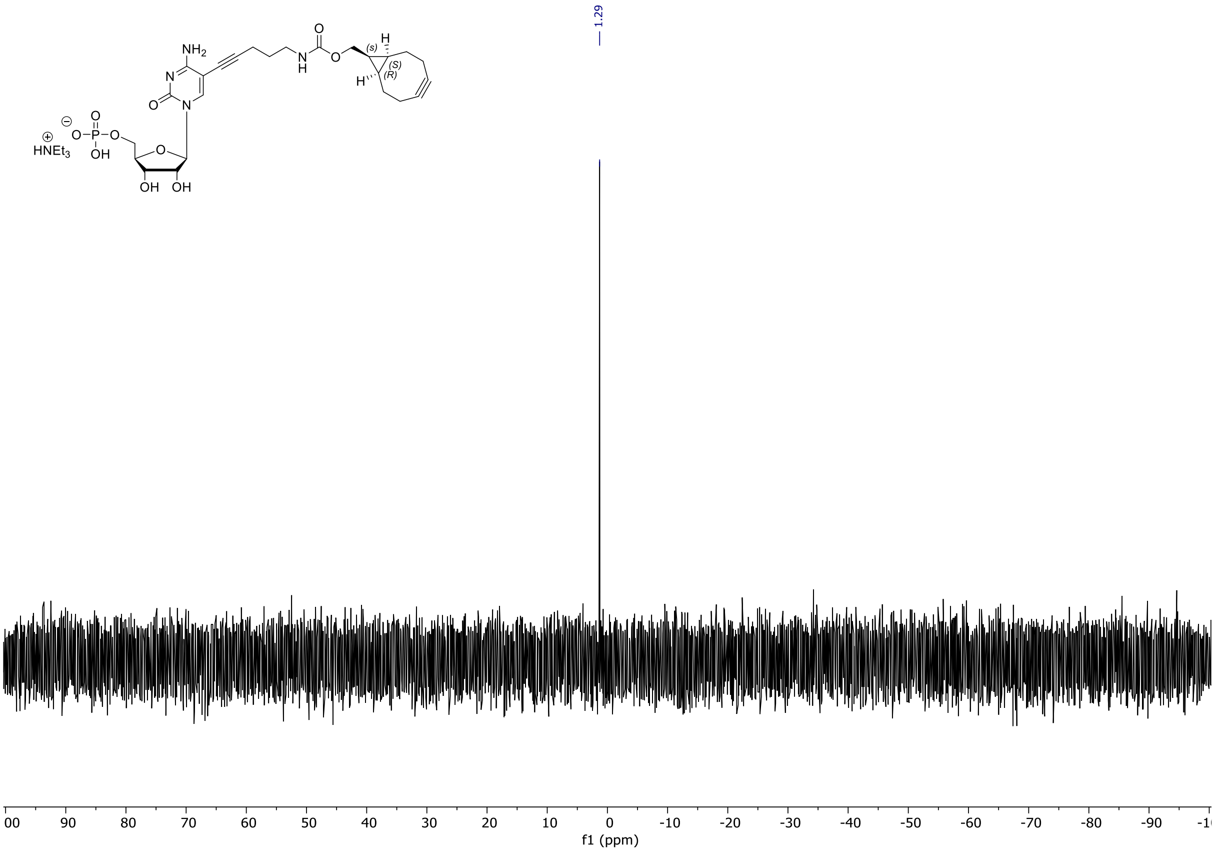
1.29
f1 (ppm)

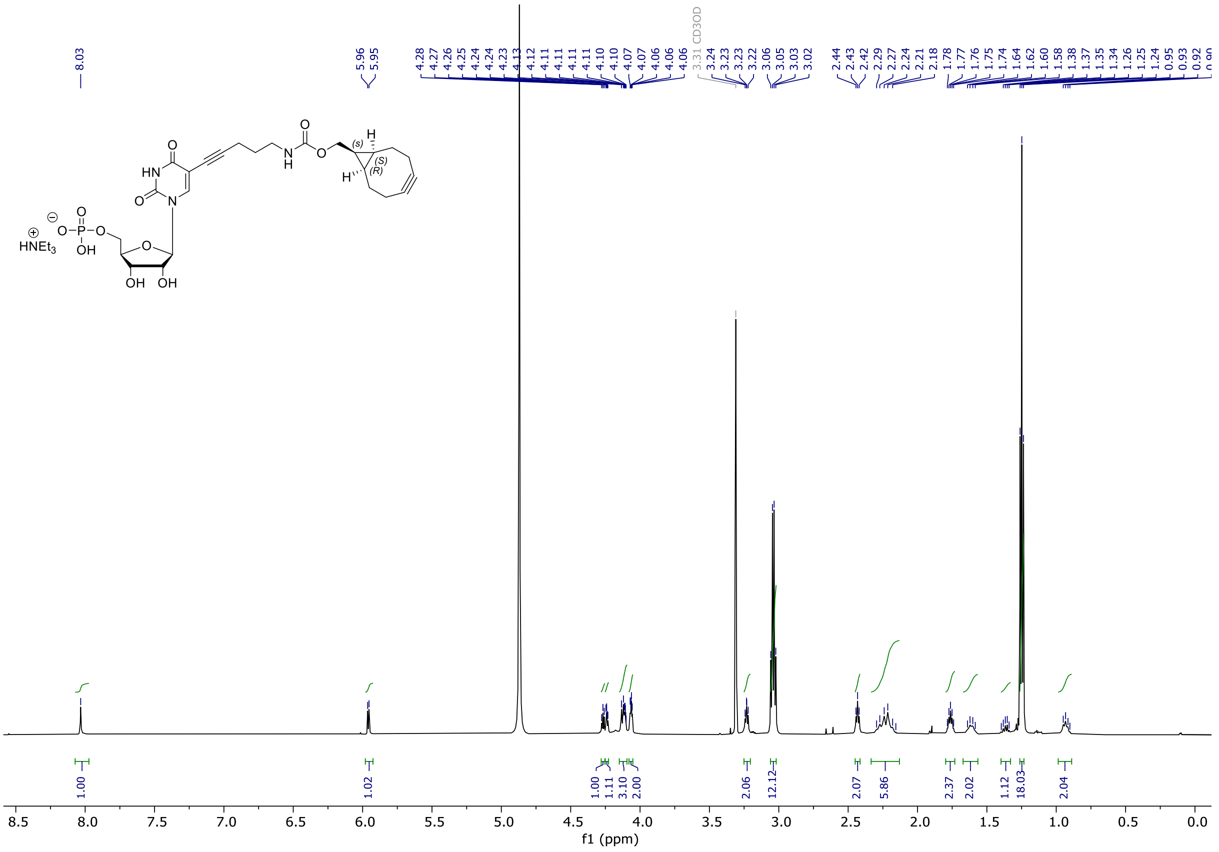
8.03
5.96
5.95
3.31 CD3OD
1.00
1.02
1.00
1.11
3.10
2.00
2.06
12.12
2.07
5.86
2.37
2.02
1.12
18.03
2.04
f1 (ppm)

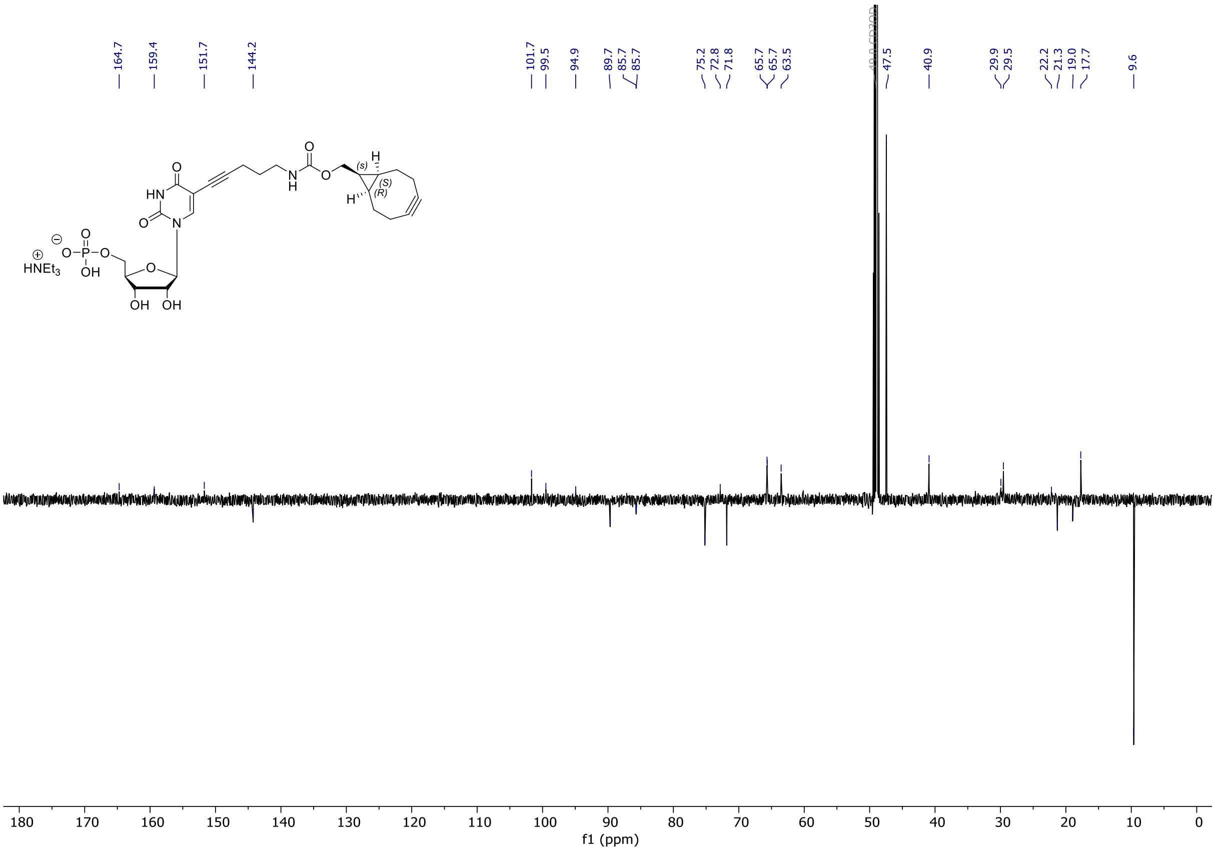
164.7
159.4
151.7
144.2
101.7
99.5
94.9
89.7
85.7
85.7
75.2
72.8
71.8
65.7
65.7
63.5
47.5
40.9
29.9
29.5
22.2
21.3
19.0
17.7
9.6
O
HN
O
N
O
N
H
O
(s)
H
(S)
(R)
H
O−P−O
O
OH
HNEt3
O
OH
OH
180
170
160
150
140
130
120
110
100
90
80
70
60
50
40
30
20
10
0
f1 (ppm)

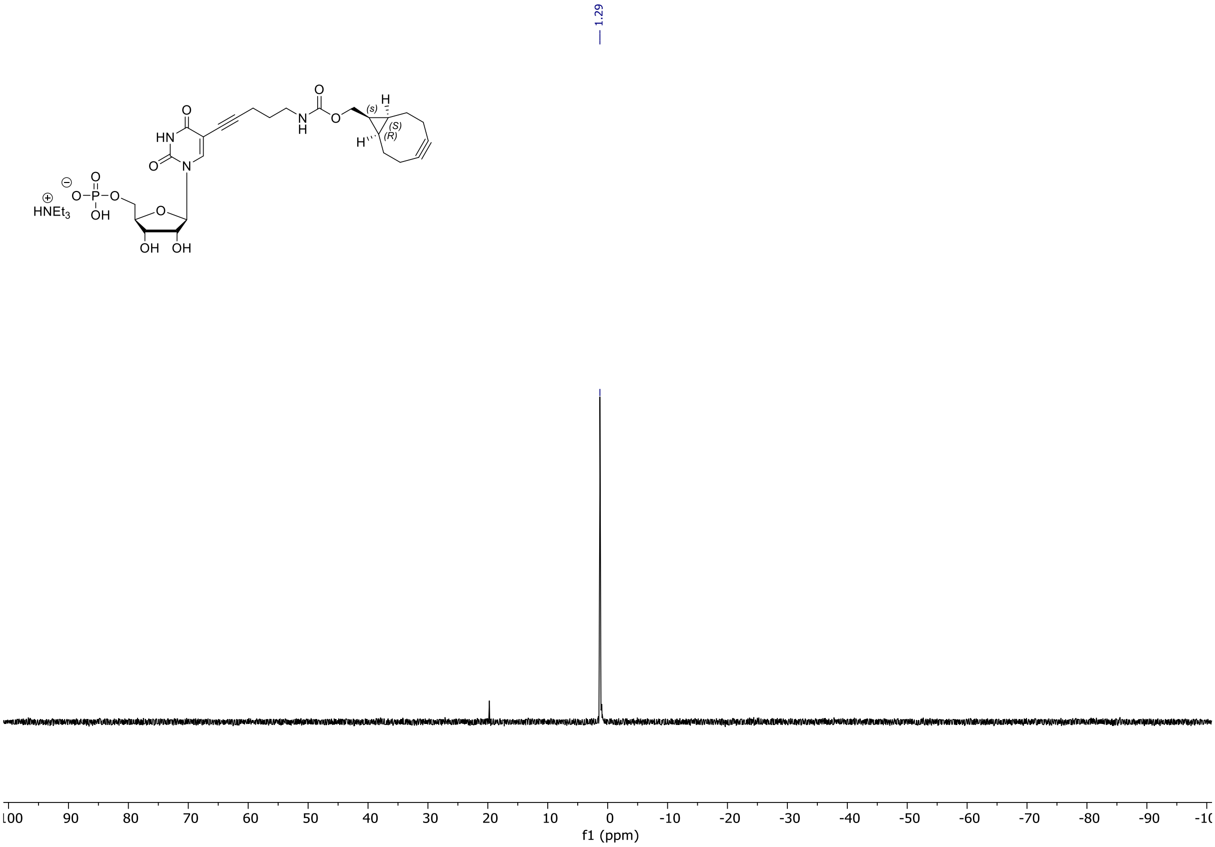
1.29
O
HN
O
N
O
N
H
O
(s)
H
(S)
(R)
H
O−P−O
O
OH
HNEt3
O
OH
OH
90
80
70
60
50
40
30
20
10
0
-10
-20
-30
-40
-50
-60
-70
-80
-90
f1 (ppm)

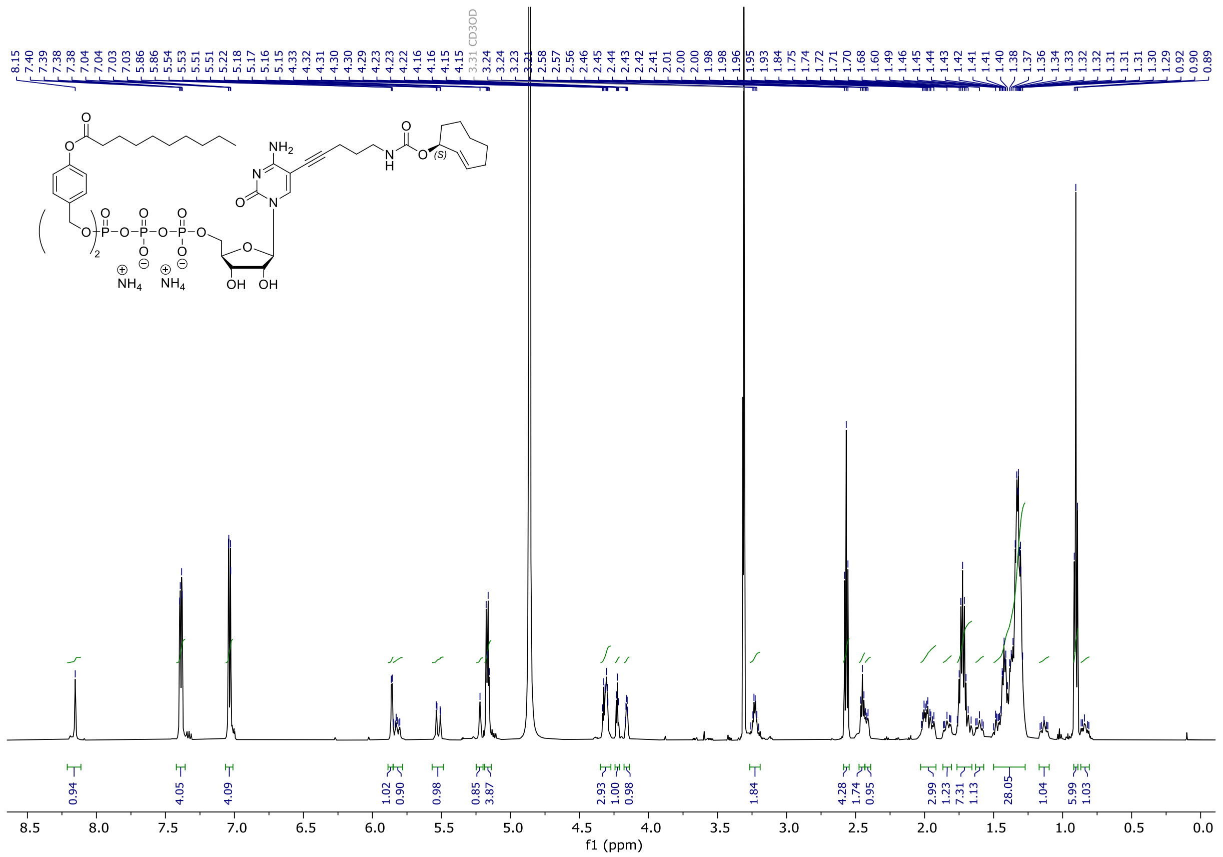

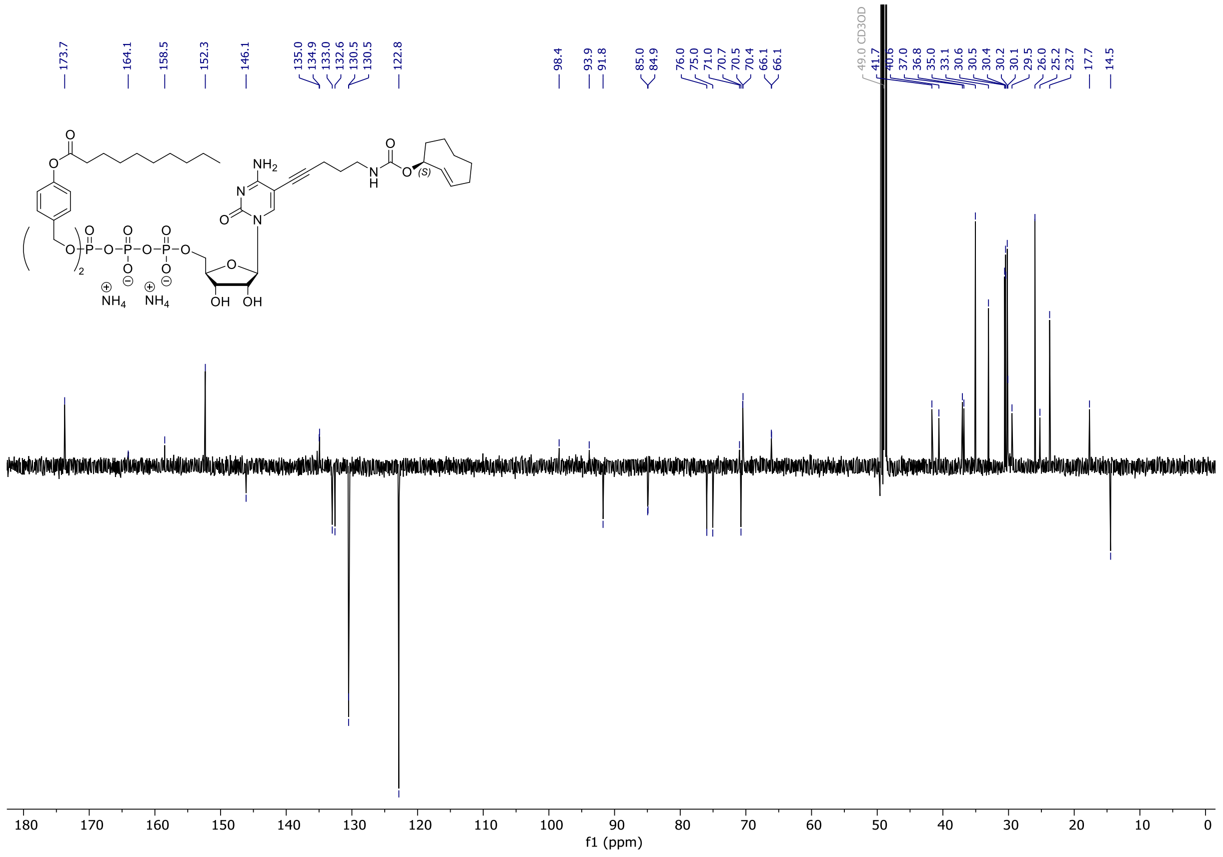

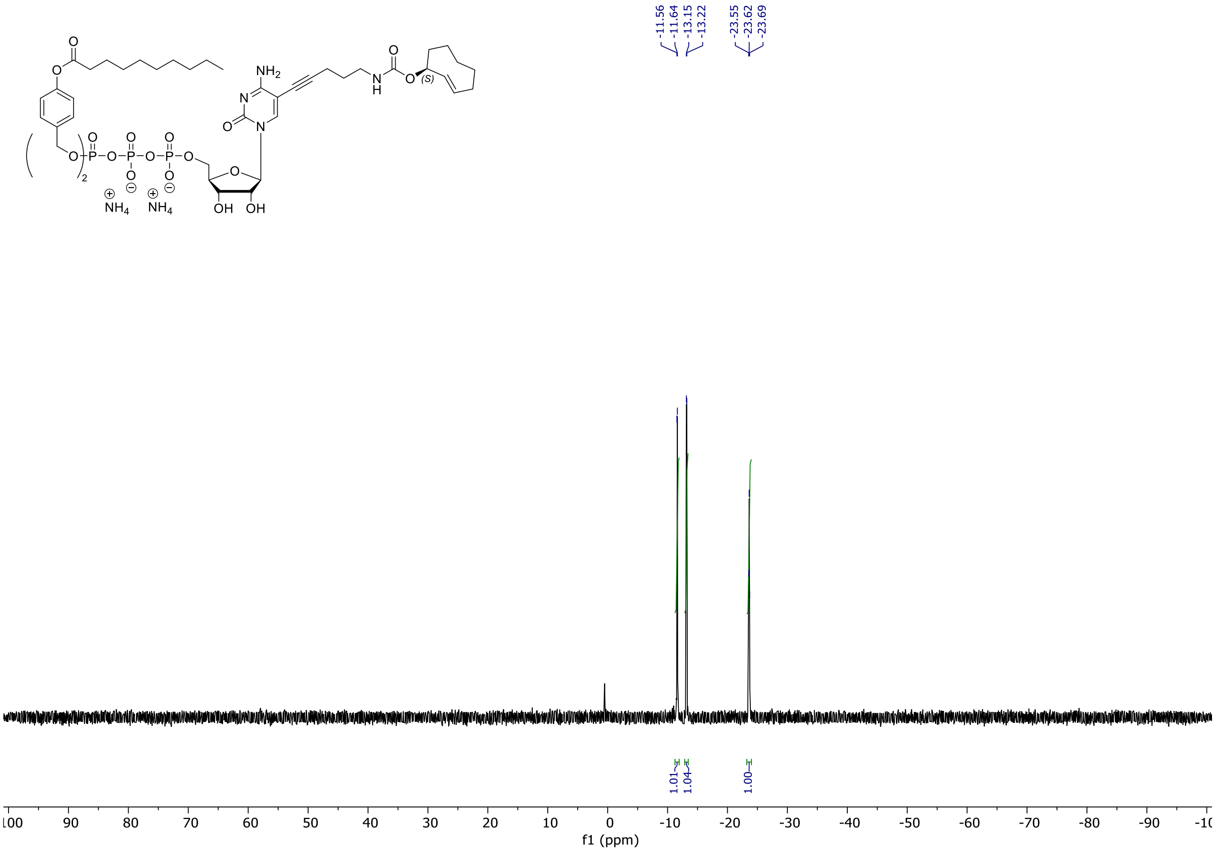

-11.56
-11.64
-13.15
-13.22
-23.55
-23.62
-23.69
NH2
N
O
N
H
(S)
O
P
NH4
OH
1.01
1.04
1.00
f1 (ppm)

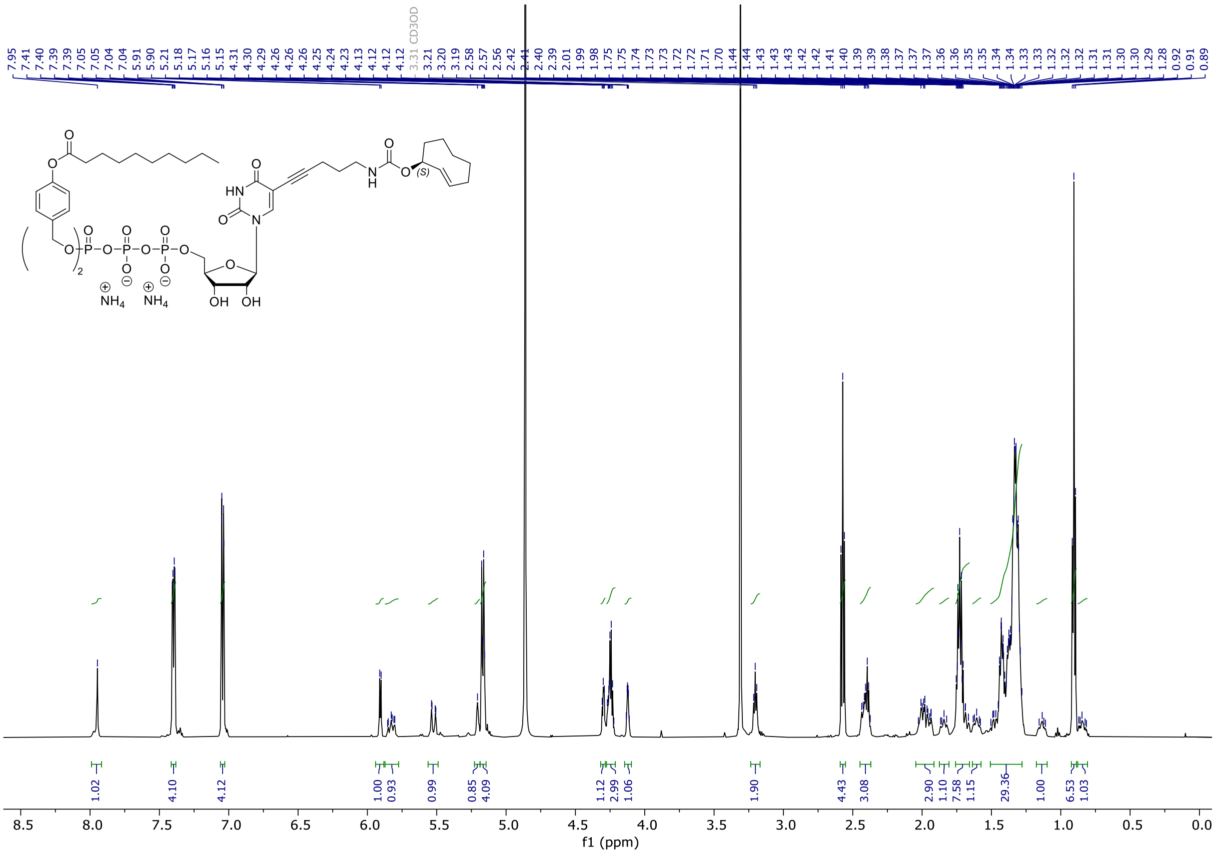

7.95 7.41 7.40 7.39 7.39 7.05 7.05 7.04 7.04 5.91 5.90 5.21 5.18 5.17 5.16 5.15 4.31 4.30 4.29 4.26 4.26 4.25 4.24 4.23 4.13 4.12 4.12 3.31 CD3OD 3.21 3.20 3.19 2.58 2.57 2.56 2.42 2.41 2.40 2.39 2.01 1.99 1.98 1.75 1.75 1.74 1.73 1.73 1.72 1.72 1.71 1.70 1.44 1.44 1.43 1.43 1.42 1.42 1.41 1.40 1.39 1.39 1.38 1.37 1.37 1.37 1.36 1.36 1.35 1.35 1.34 1.34 1.33 1.33 1.32 1.32 1.32 1.31 1.31 1.30 1.30 1.29 1.28 0.92 0.91 0.89
HN
O
N
H
(S)
NH4
OH
1.02
4.10
4.12
1.00
0.93
0.99
0.85
4.09
1.12
2.99
1.06
1.90
4.43
3.08
2.90
1.10
7.58
1.15
29.36
1.00
6.53
1.03
f1 (ppm)

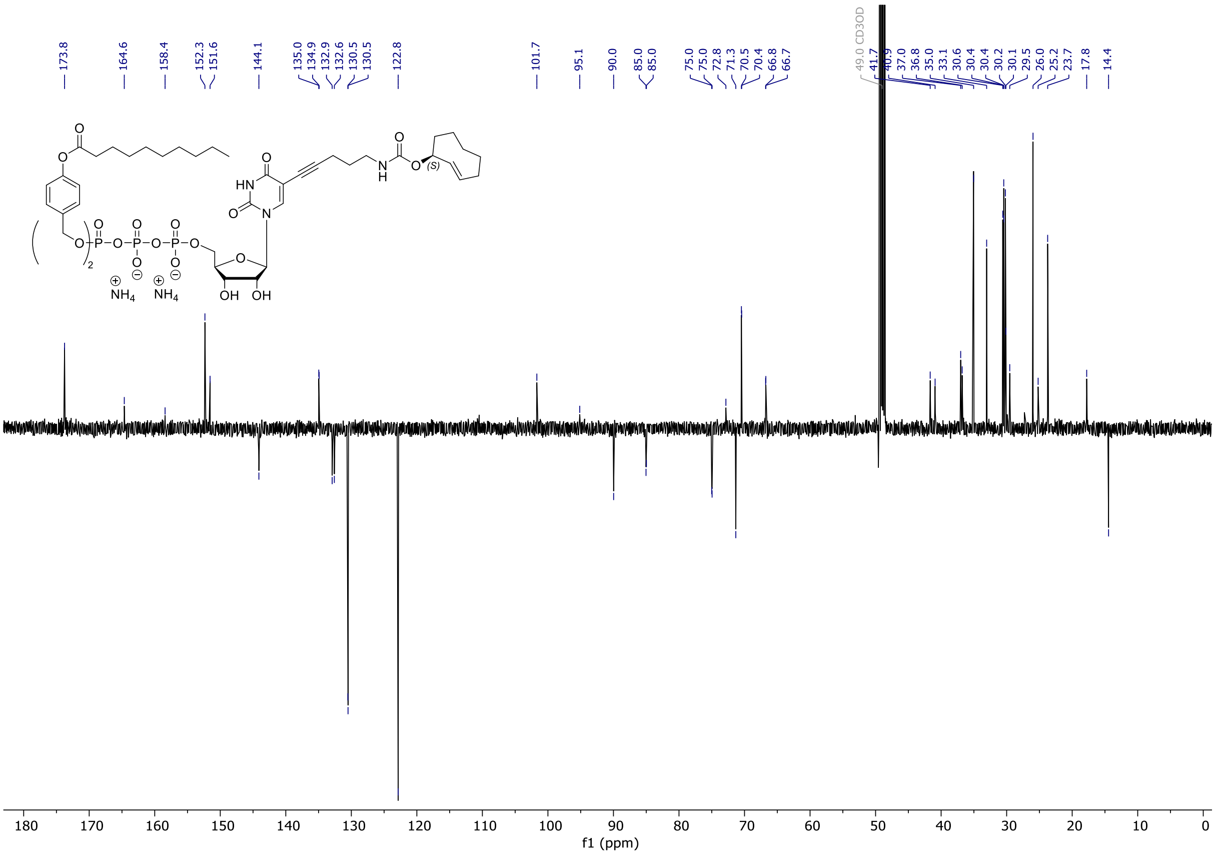
173.8
164.6
158.4
152.3
151.6
144.1
135.0
134.9
132.9
132.6
130.5
130.5
122.8
101.7
95.1
90.0
85.0
85.0
75.0
75.0
72.8
71.3
70.5
70.4
66.8
66.7
49.0 CD3OD
41.7
40.9
37.0
36.8
35.0
33.1
30.6
30.4
30.4
30.2
30.1
29.5
26.0
25.2
23.7
17.8
14.4
f1 (ppm)

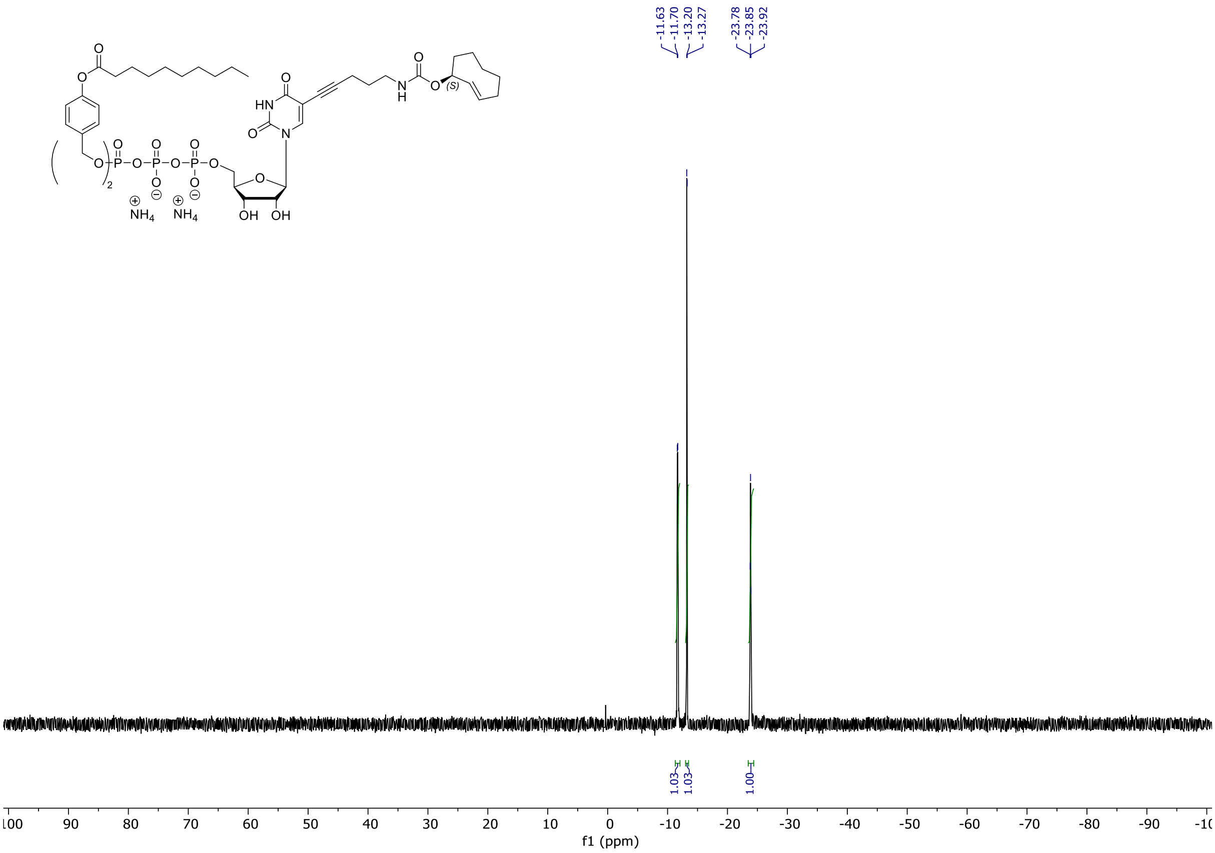
-11.63
-11.70
-13.20
-13.27
-23.78
-23.85
-23.92
1.03
1.03
1.00
f1 (ppm)

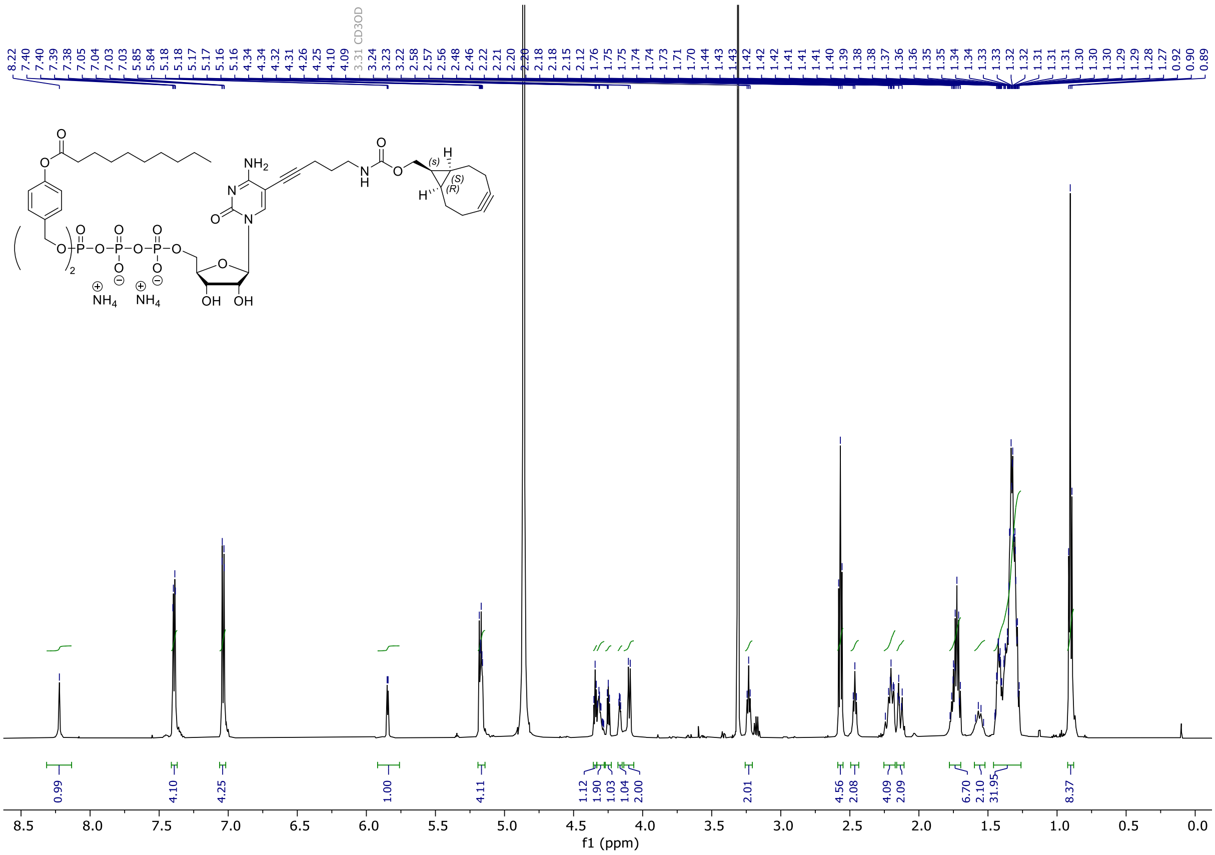

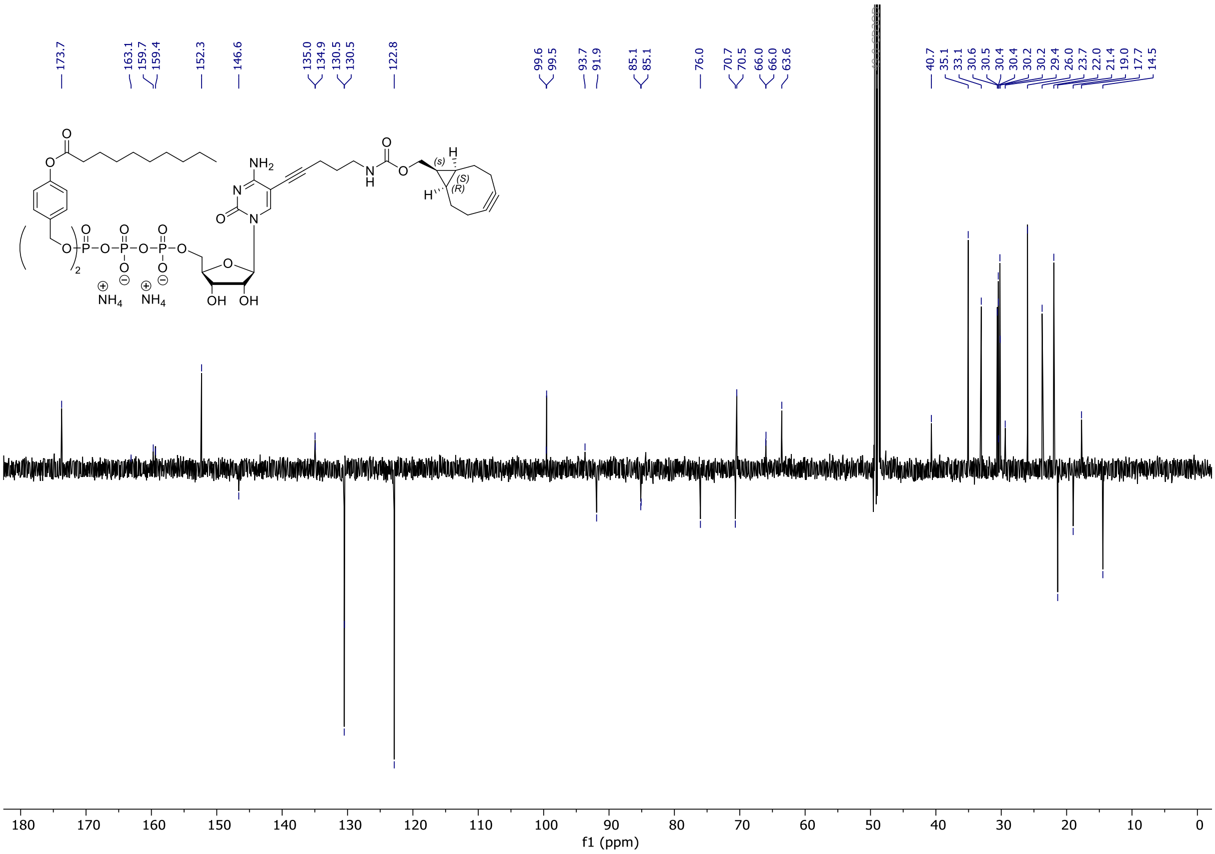

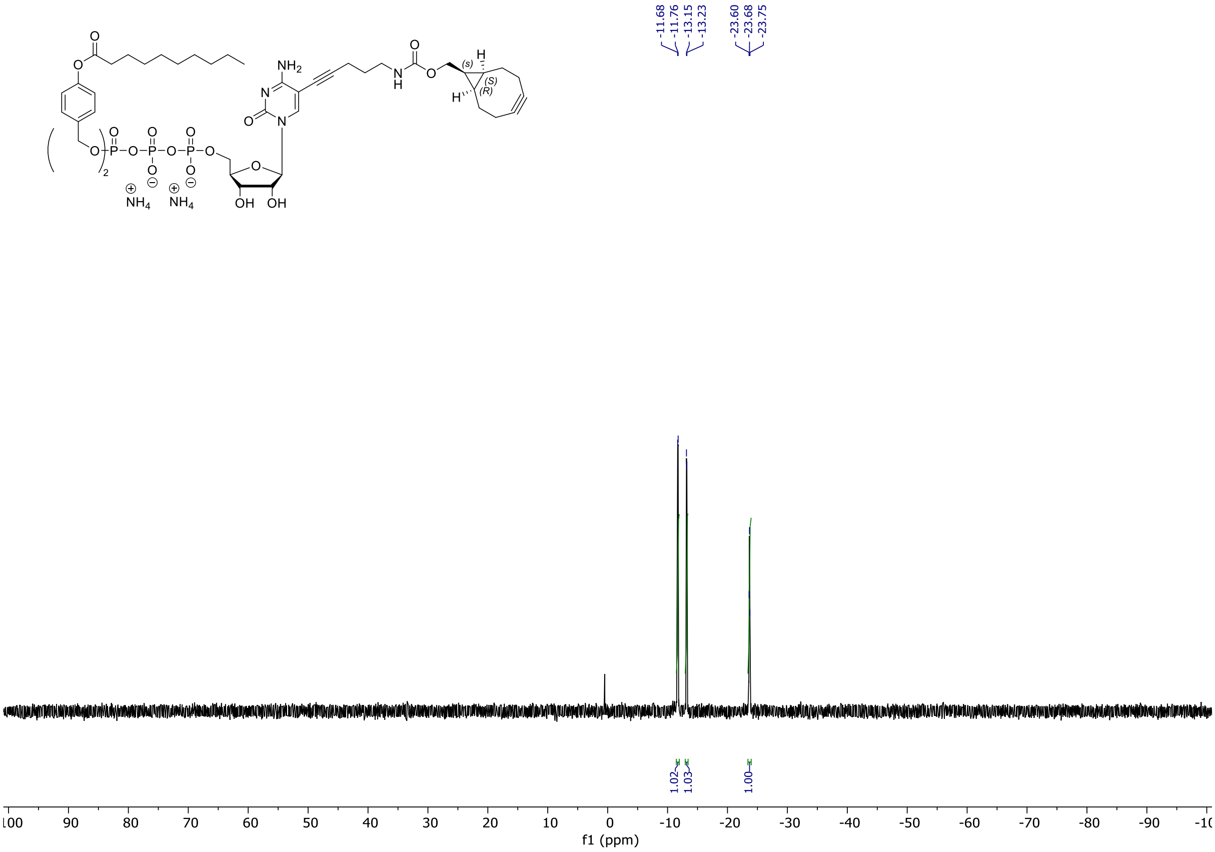
-11.68
-11.76
-13.15
-13.23
-23.60
-23.68
-23.75
NH2
N
N
O
NH4
OH OH
1.02
1.03
1.00
f1 (ppm)

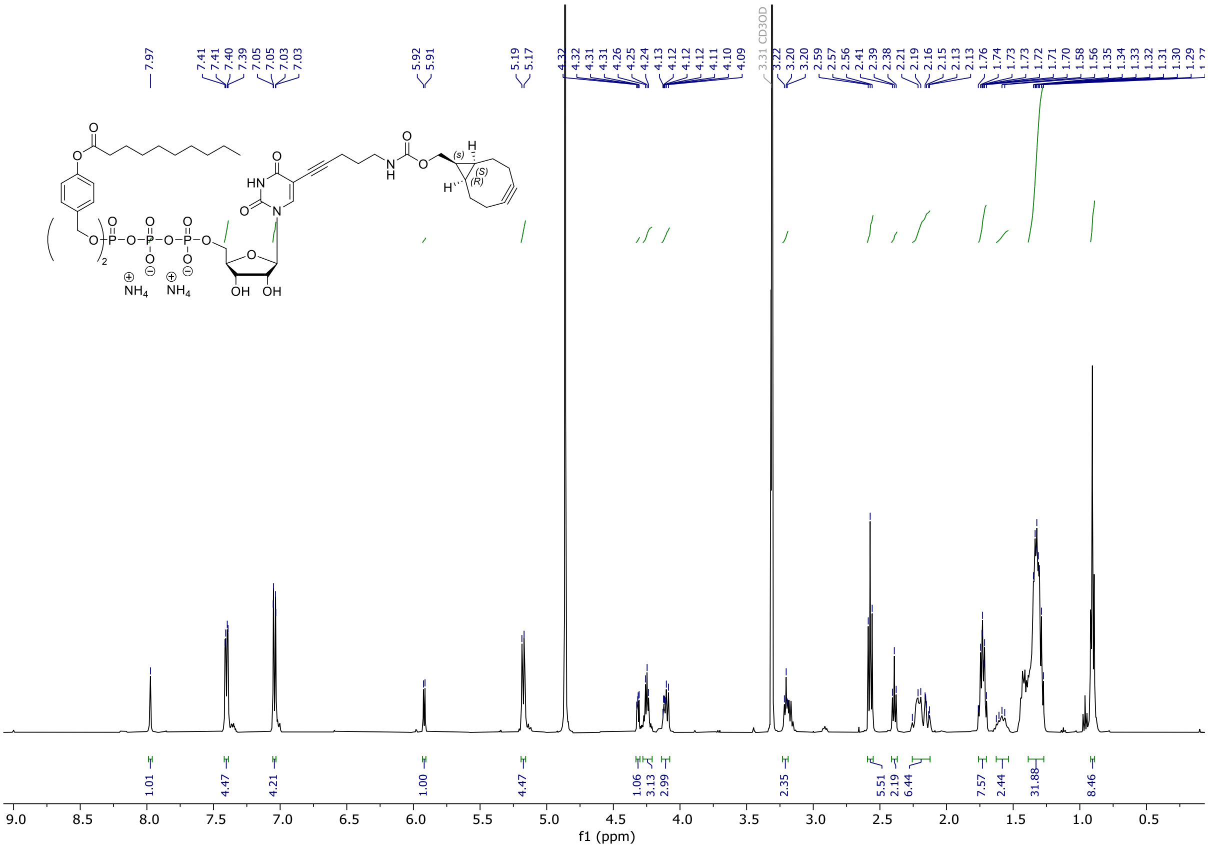
7.97
7.41 7.41 7.40 7.39 7.05 7.05 7.03 7.03
5.92 5.91
5.19 5.17
4.32 4.32 4.31 4.31 4.26 4.25 4.24 4.13 4.12 4.12 4.12 4.12 4.11 4.10 4.09
3.31 CD3OD
3.22 3.20 3.20
2.59 2.57 2.56 2.41 2.39 2.38 2.21 2.19 2.16 2.15 2.13 2.13
1.76 1.74 1.73 1.72 1.71 1.70 1.58 1.56 1.35 1.34 1.33 1.32 1.31 1.30 1.29
HN
O
NH4
OH OH
1.01
4.47
4.21
1.00
4.47
1.06
3.13
2.99
2.35
5.51
2.19
6.44
7.57
2.44
31.88
8.46
f1 (ppm)

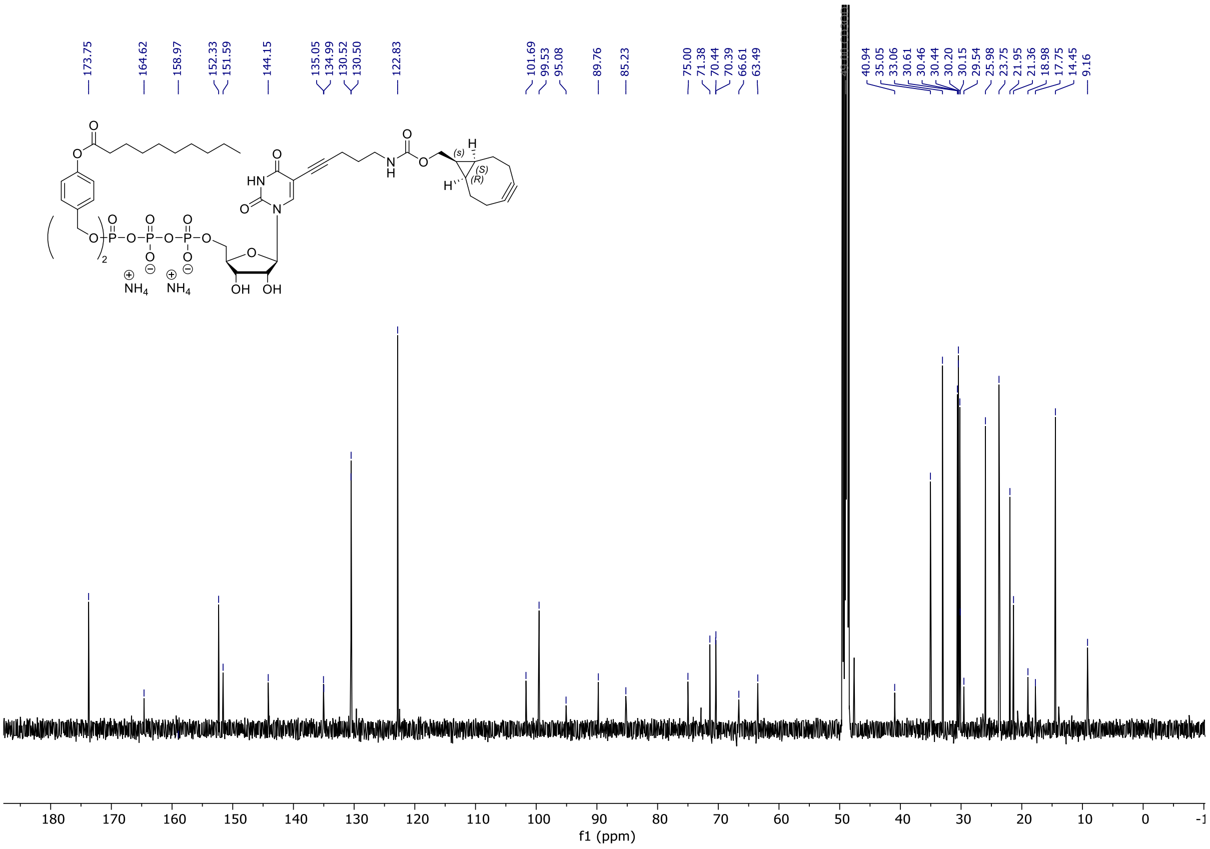
173.75
164.62
158.97
152.33
151.59
144.15
135.05
134.99
130.52
130.50
122.83
101.69
99.53
95.08
89.76
85.23
75.00
71.38
70.44
70.39
66.61
63.49
40.94
35.05
33.06
30.61
30.46
30.44
30.20
30.15
29.54
25.98
23.75
21.95
21.36
18.98
17.75
14.45
9.16
f1 (ppm)

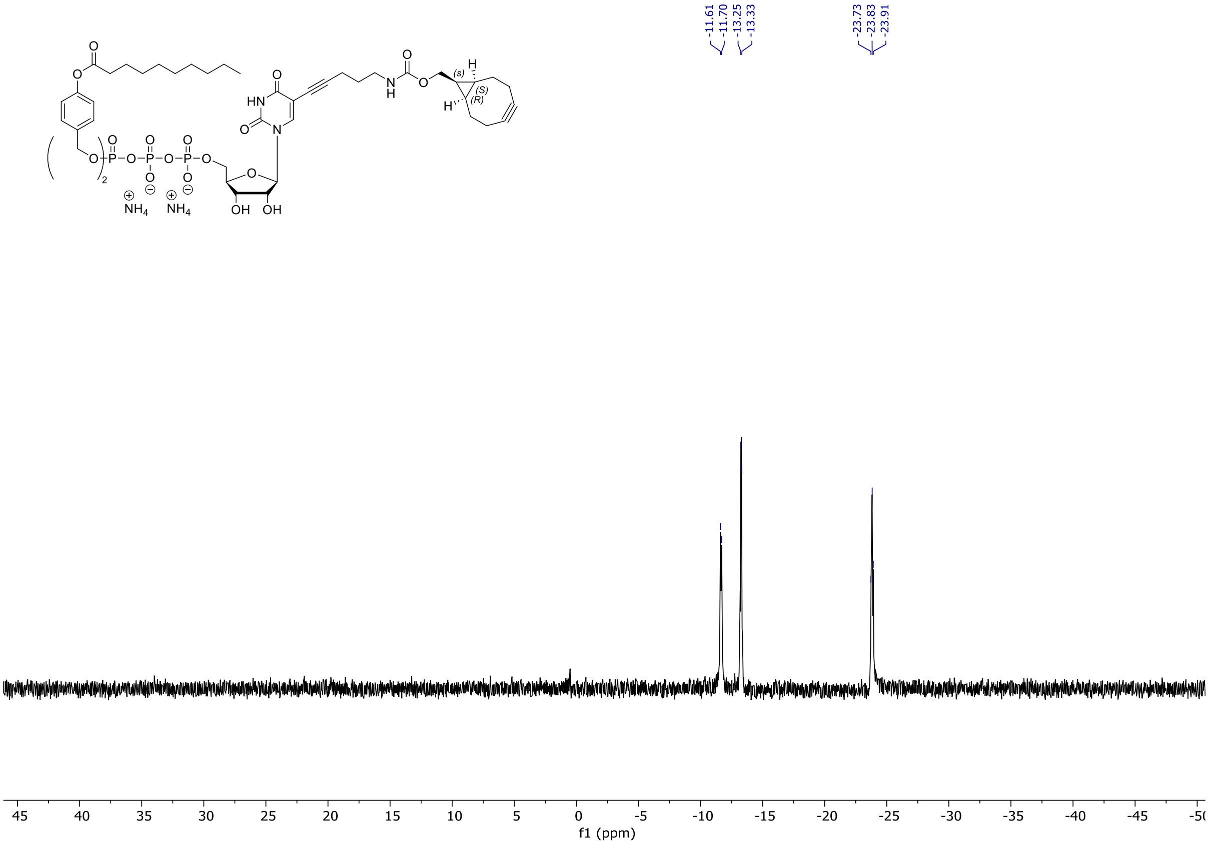
-11.61
-11.70
-13.25
-13.33
-23.73
-23.83
-23.91
f1 (ppm)

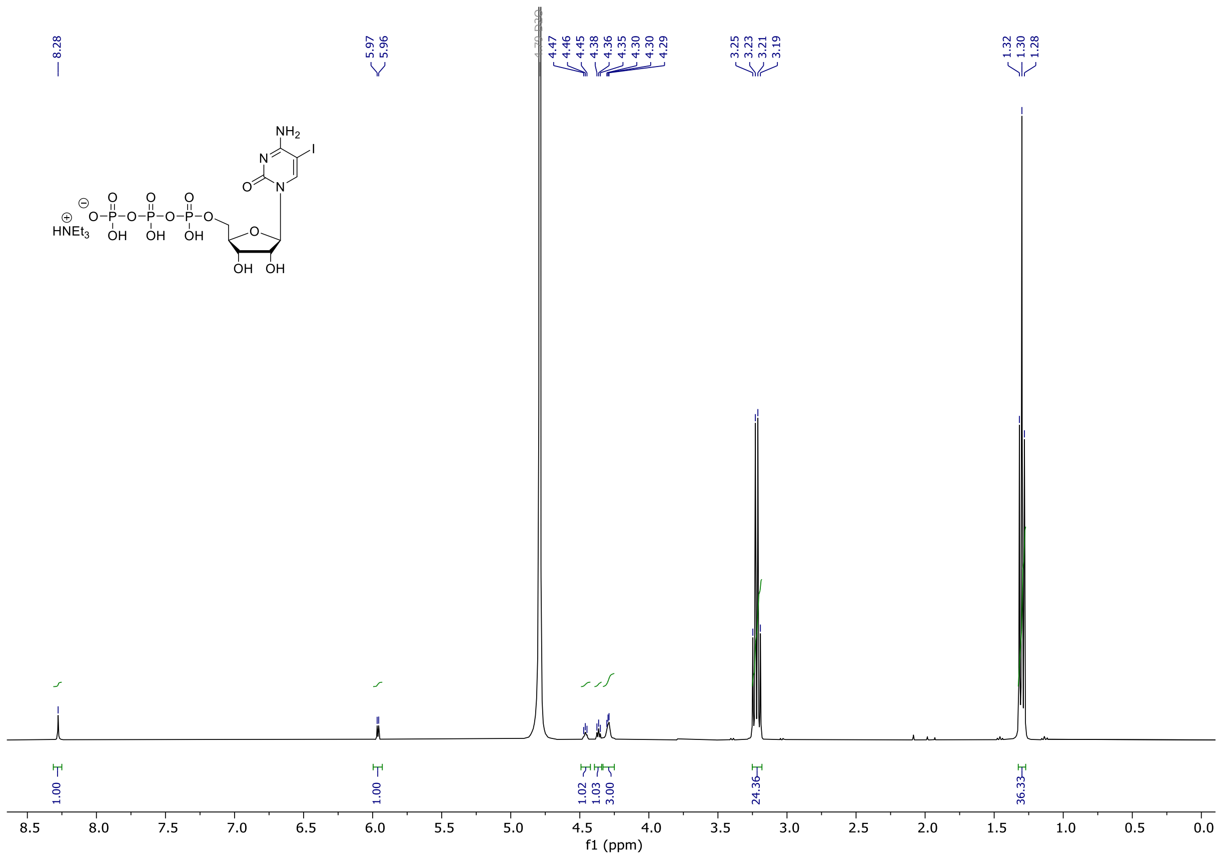
8.28
5.97
5.96
4.47
4.46
4.45
4.38
4.36
4.35
4.30
4.30
4.29
3.25
3.23
3.21
3.19
1.32
1.30
1.28
NH2
N
I
O
N
O
O
O
O
HNEt3
OH
OH
OH
OH
OH
1.00
1.00
1.02
1.03
3.00
24.36
36.33
f1 (ppm)

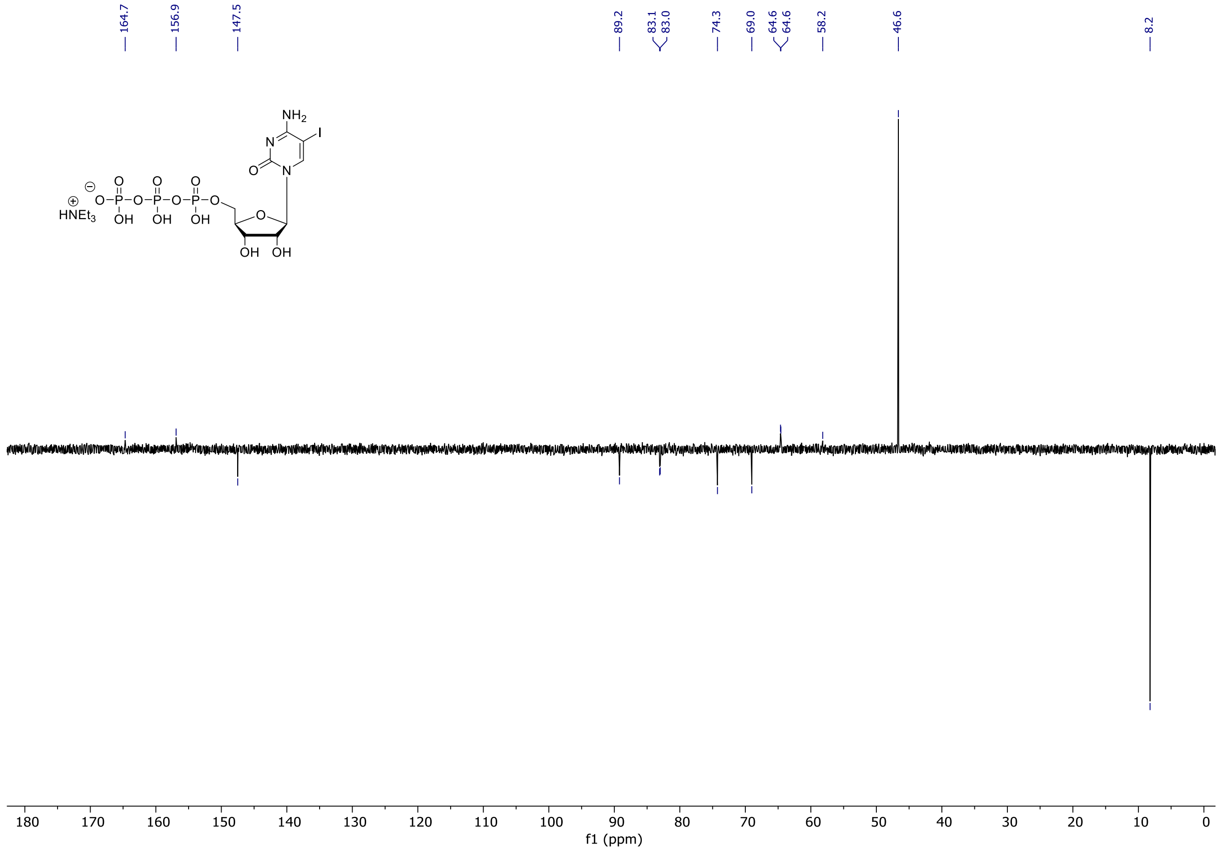
164.7
156.9
147.5
89.2
83.1
83.0
74.3
69.0
64.6
64.6
58.2
46.6
8.2
NH2
HNEt3
OH
OH
f1 (ppm)

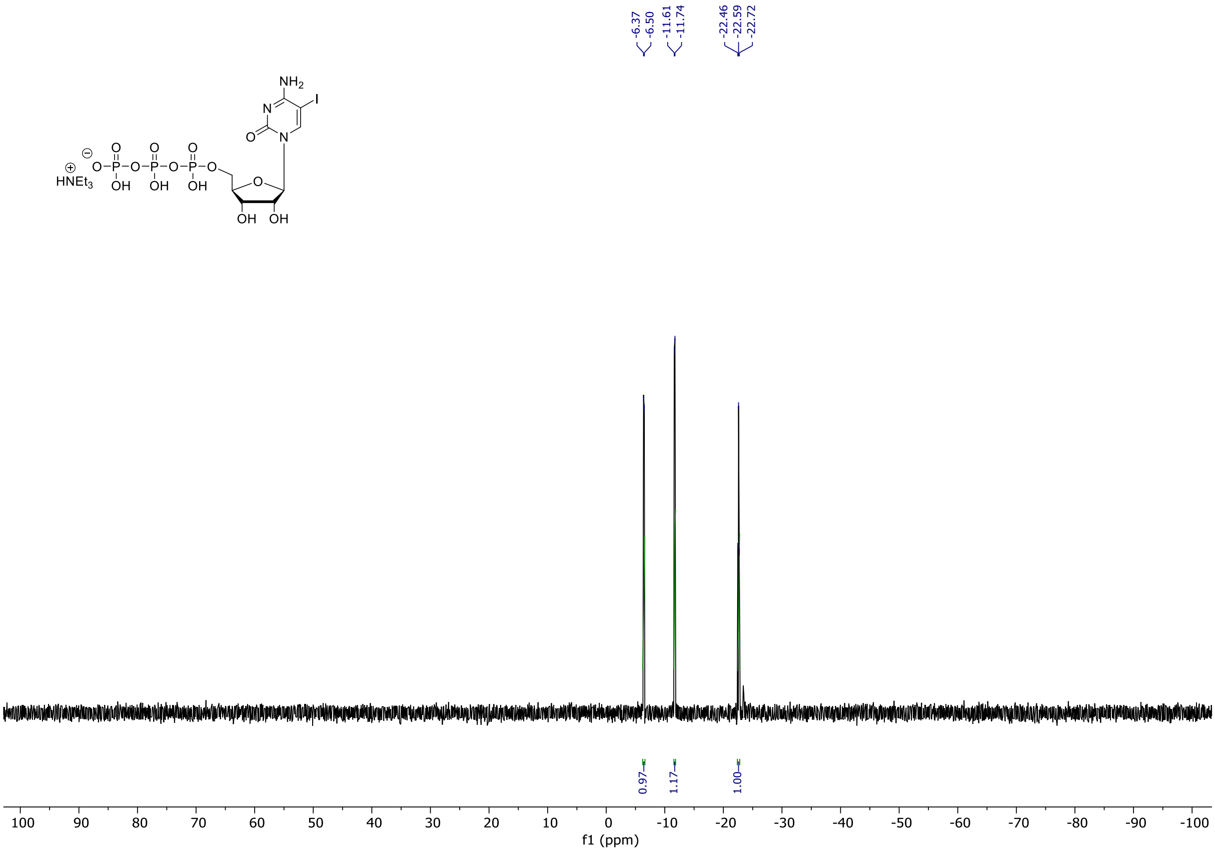
-6.37
-6.50
-11.61
-11.74
-22.46
-22.59
-22.72
0.97
1.17
1.00
f1 (ppm)

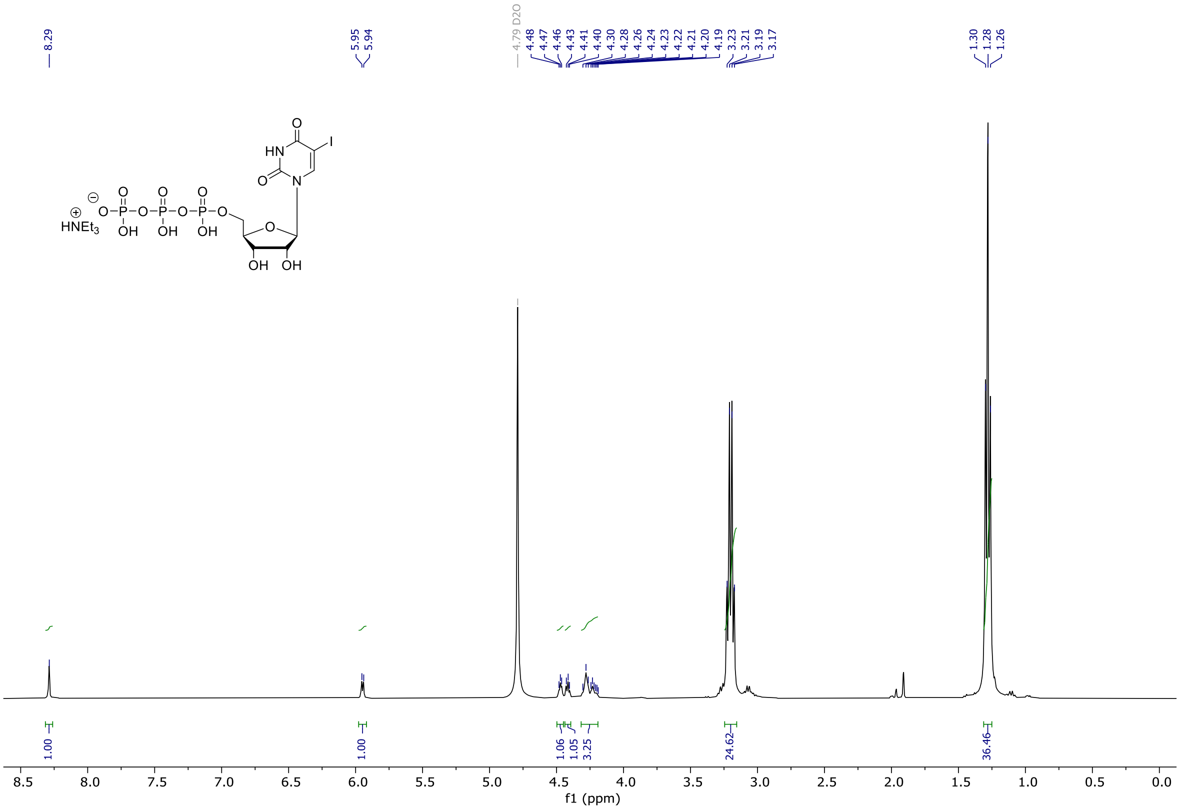
8.29
5.95
5.94
4.79 D2O
4.48
4.47
4.46
4.43
4.41
4.40
4.30
4.28
4.26
4.24
4.23
4.22
4.21
4.20
4.19
3.23
3.21
3.19
3.17
1.30
1.28
1.26
1.00
1.00
1.06
1.05
3.25
24.62
36.46
f1 (ppm)

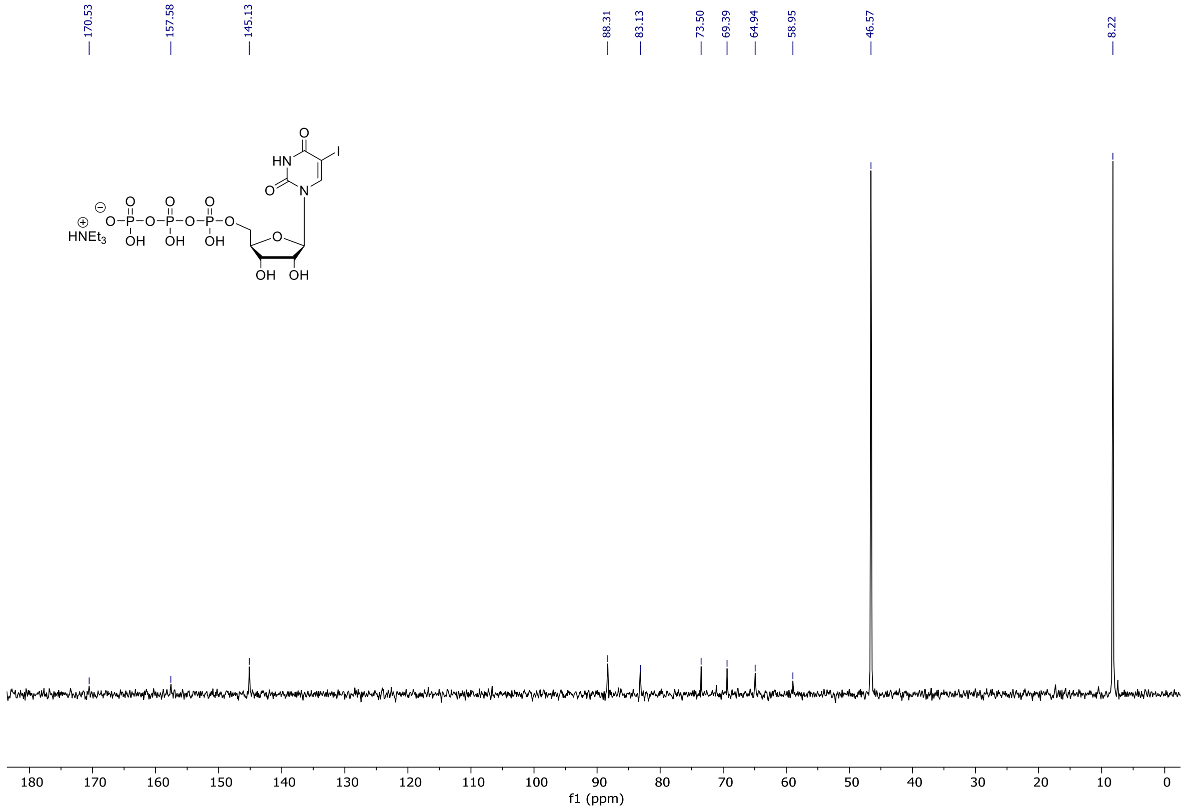

170.53
157.58
145.13
88.31
83.13
73.50
69.39
64.94
58.95
46.57
8.22
HNEt3
OH
HN
O
I
N
f1 (ppm)

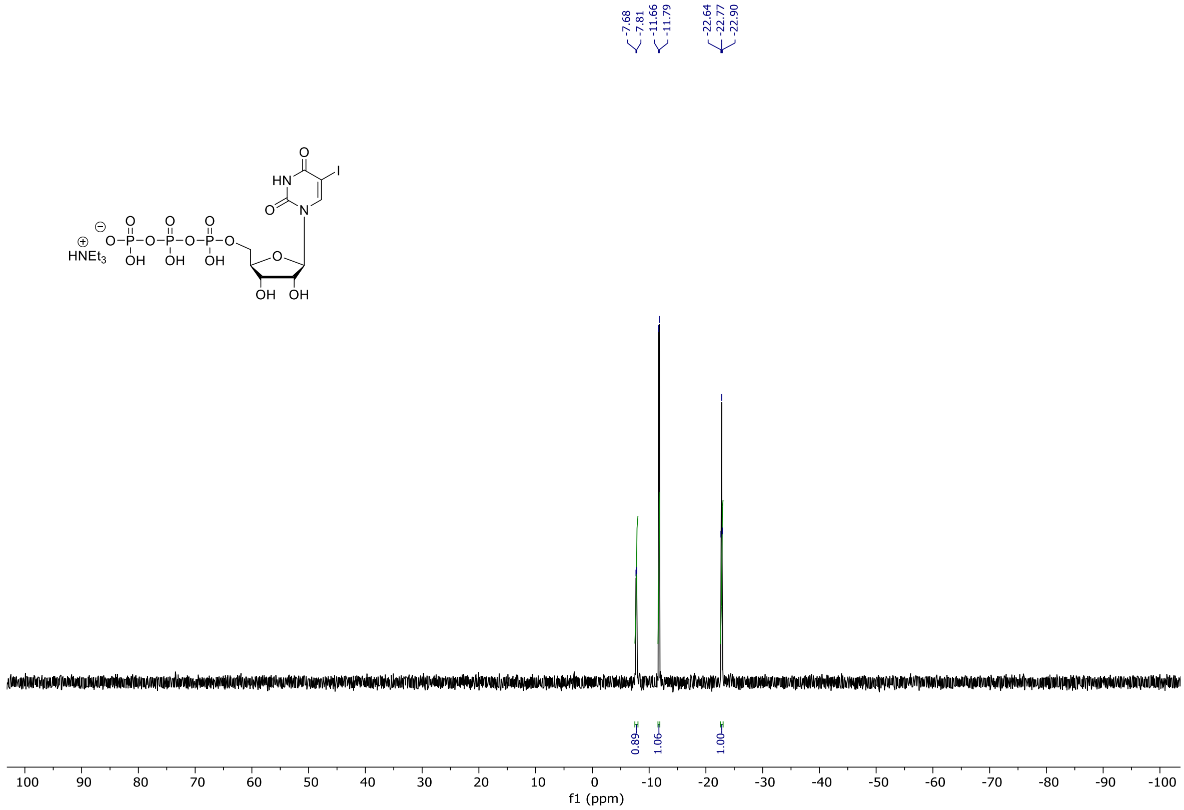

-7.68
-7.81
-11.66
-11.79
-22.64
-22.77
-22.90
HNEt3
OH
HN
O
I
N
0.89
1.06
1.00
f1 (ppm)

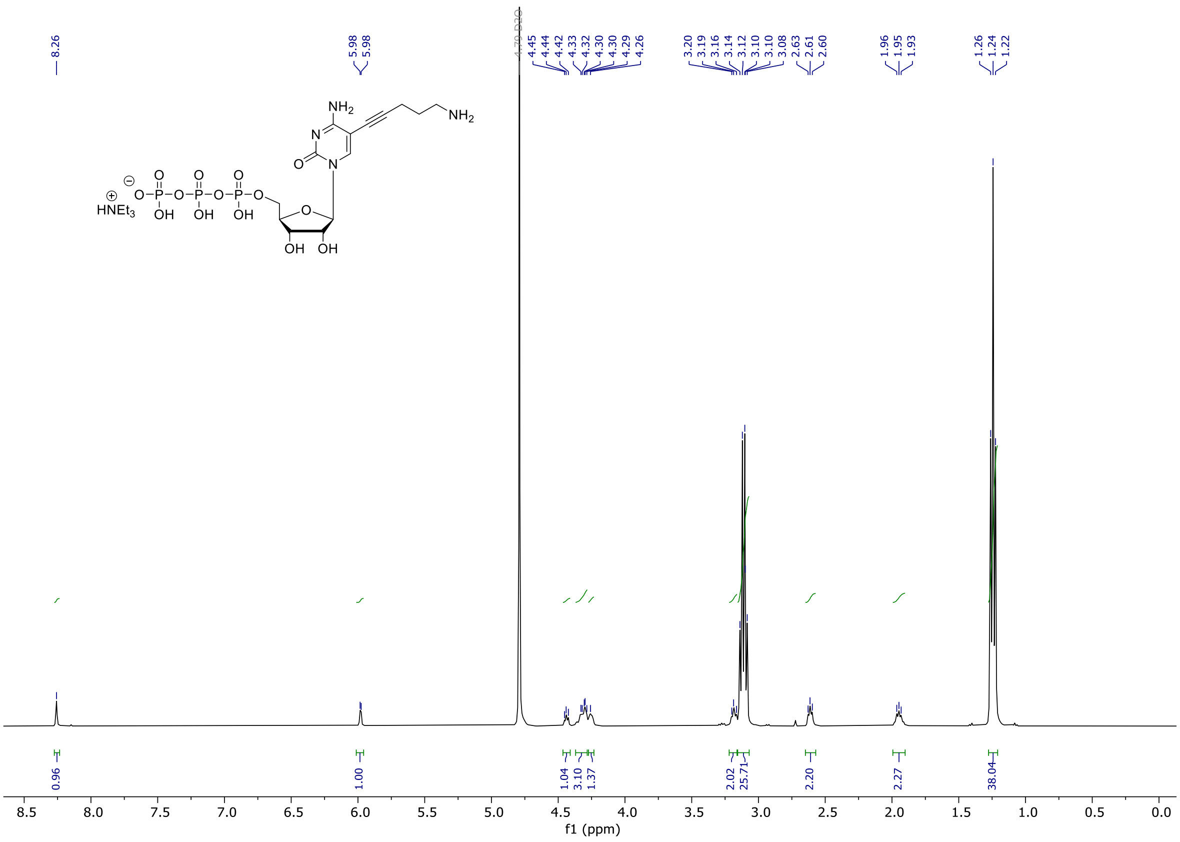

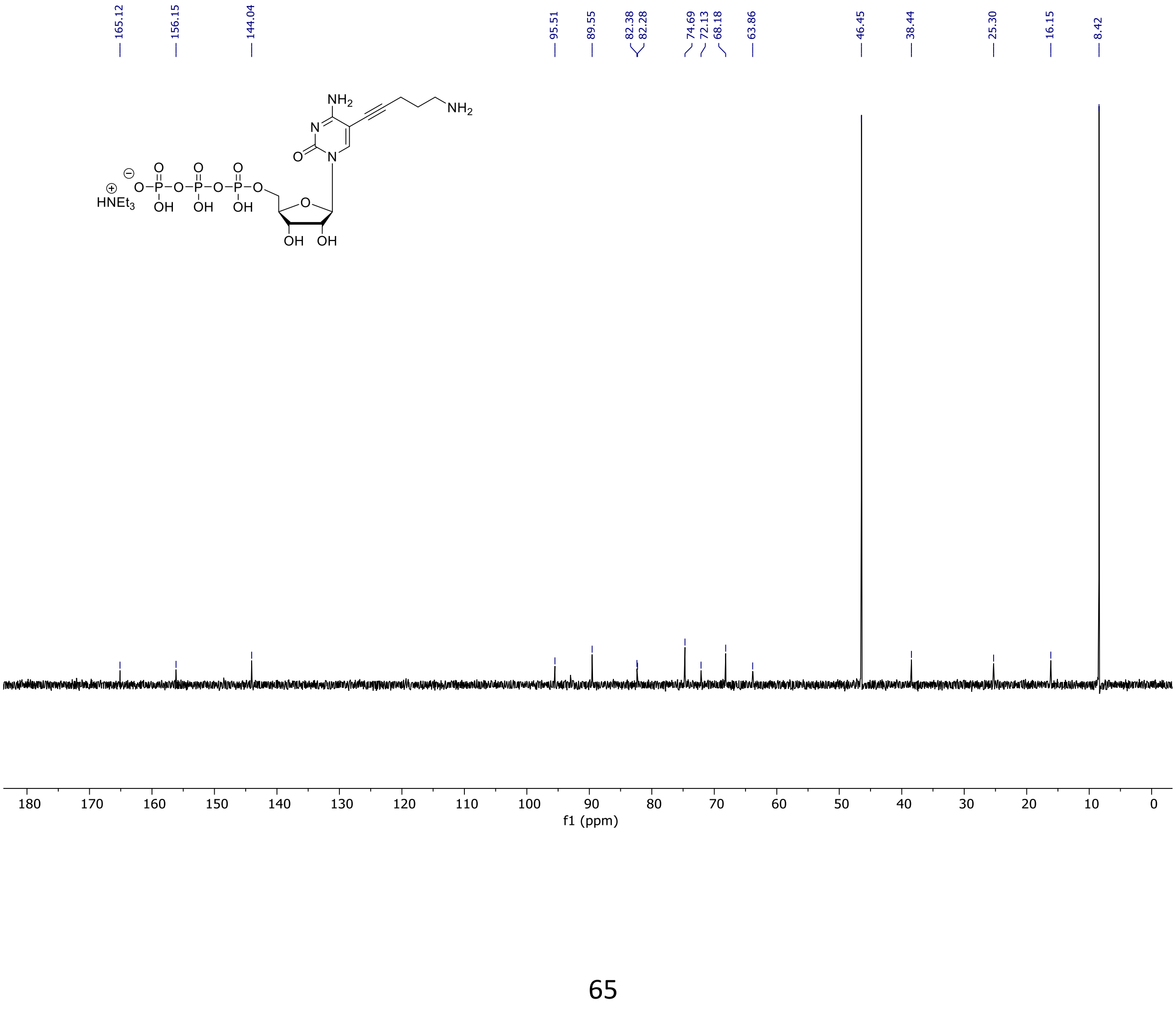

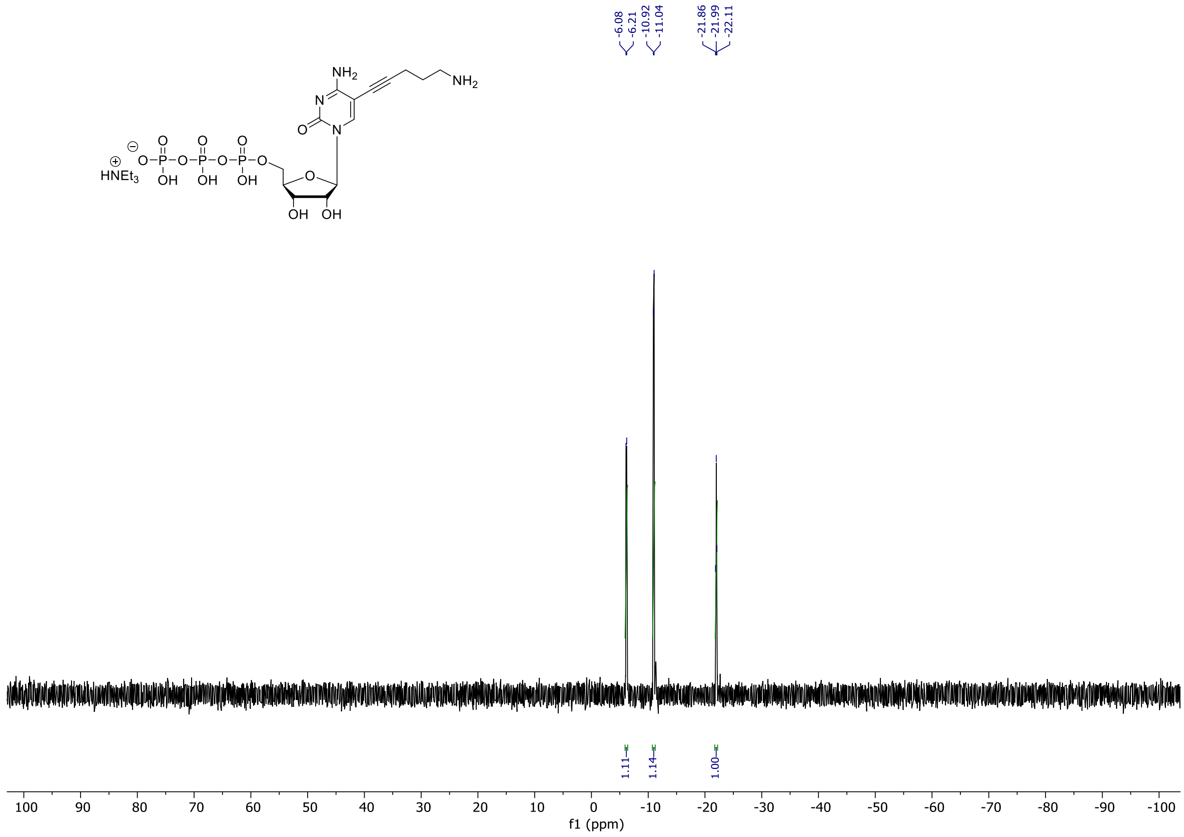

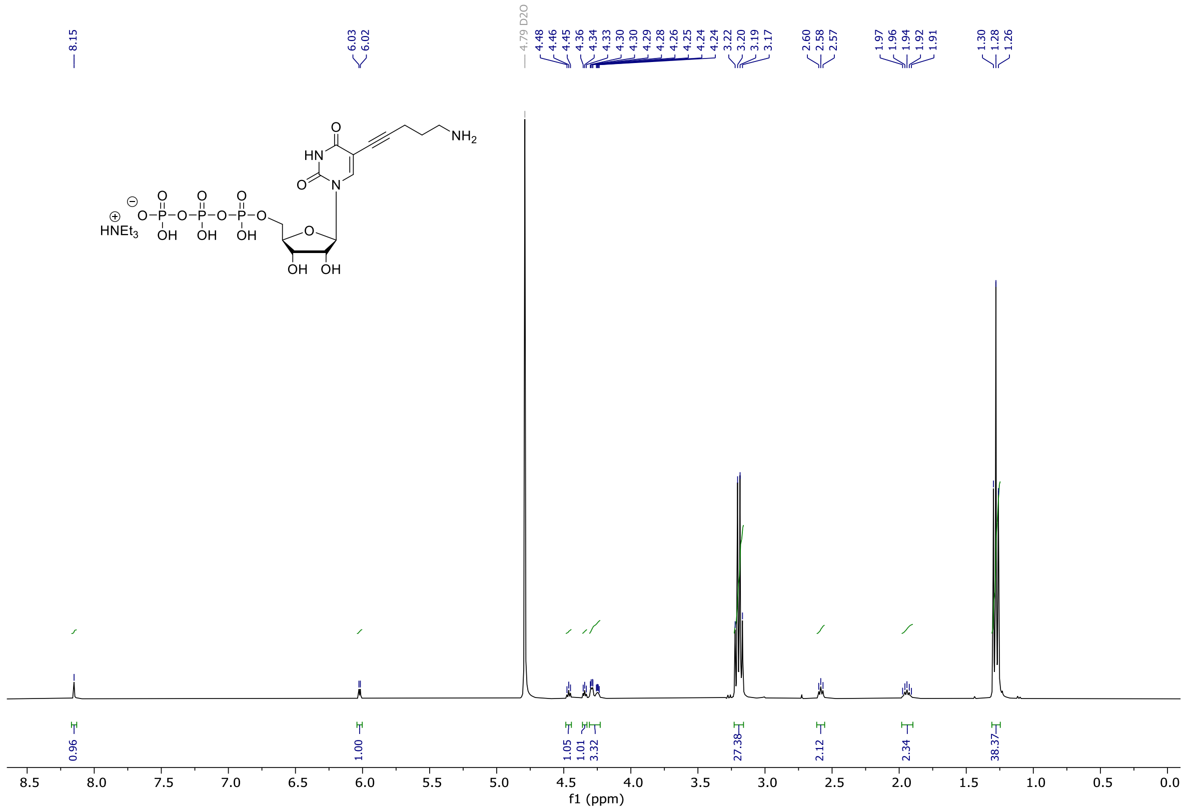

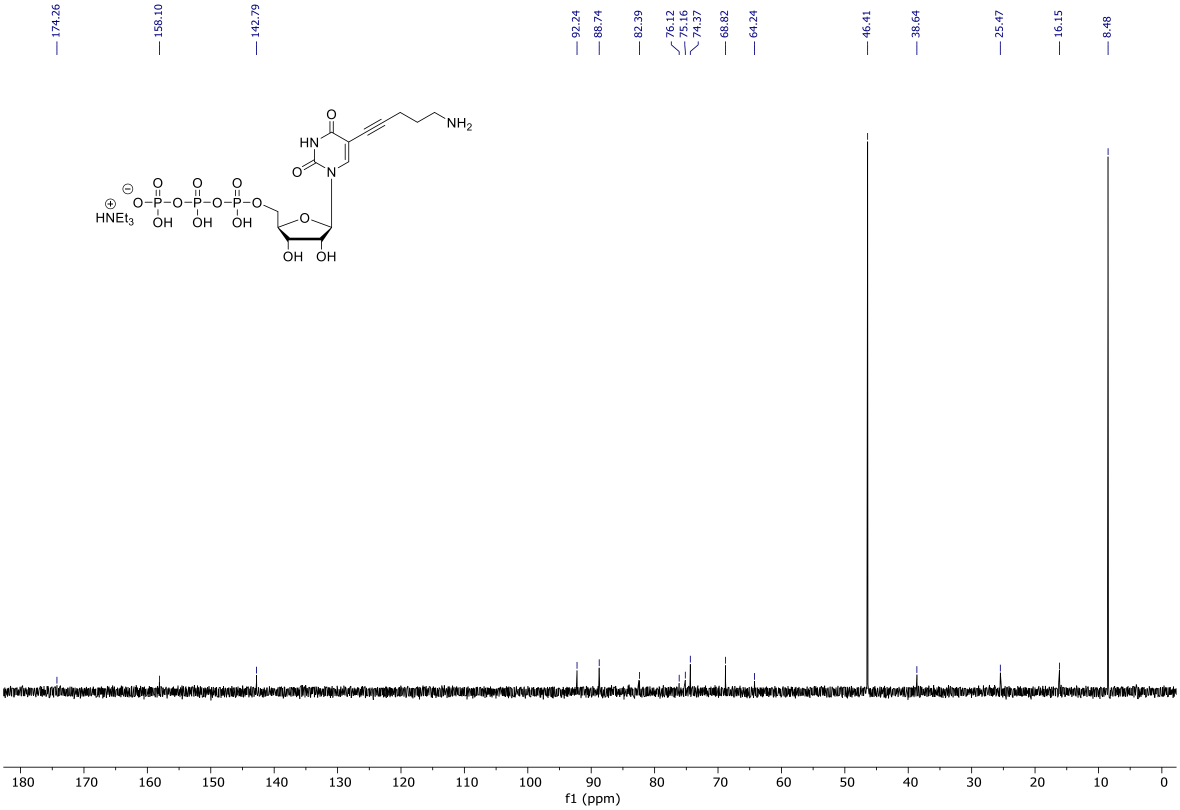

174.26
158.10
142.79
92.24
88.74
82.39
76.12
75.16
74.37
68.82
64.24
46.41
38.64
25.47
16.15
8.48
HNEt3
NH2
f1 (ppm)

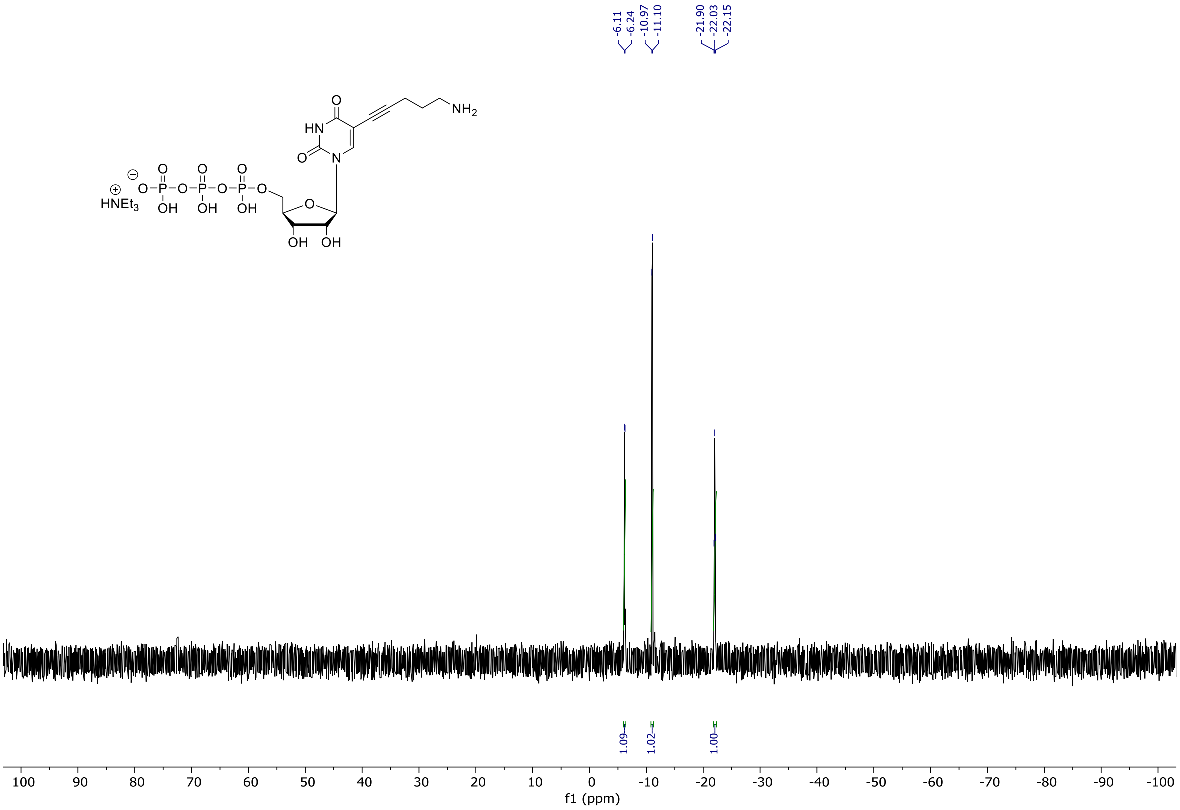

-6.11
-6.24
-10.97
-11.10
-21.90
-22.03
-22.15
HNEt3
NH2
1.09
1.02
1.00
f1 (ppm)

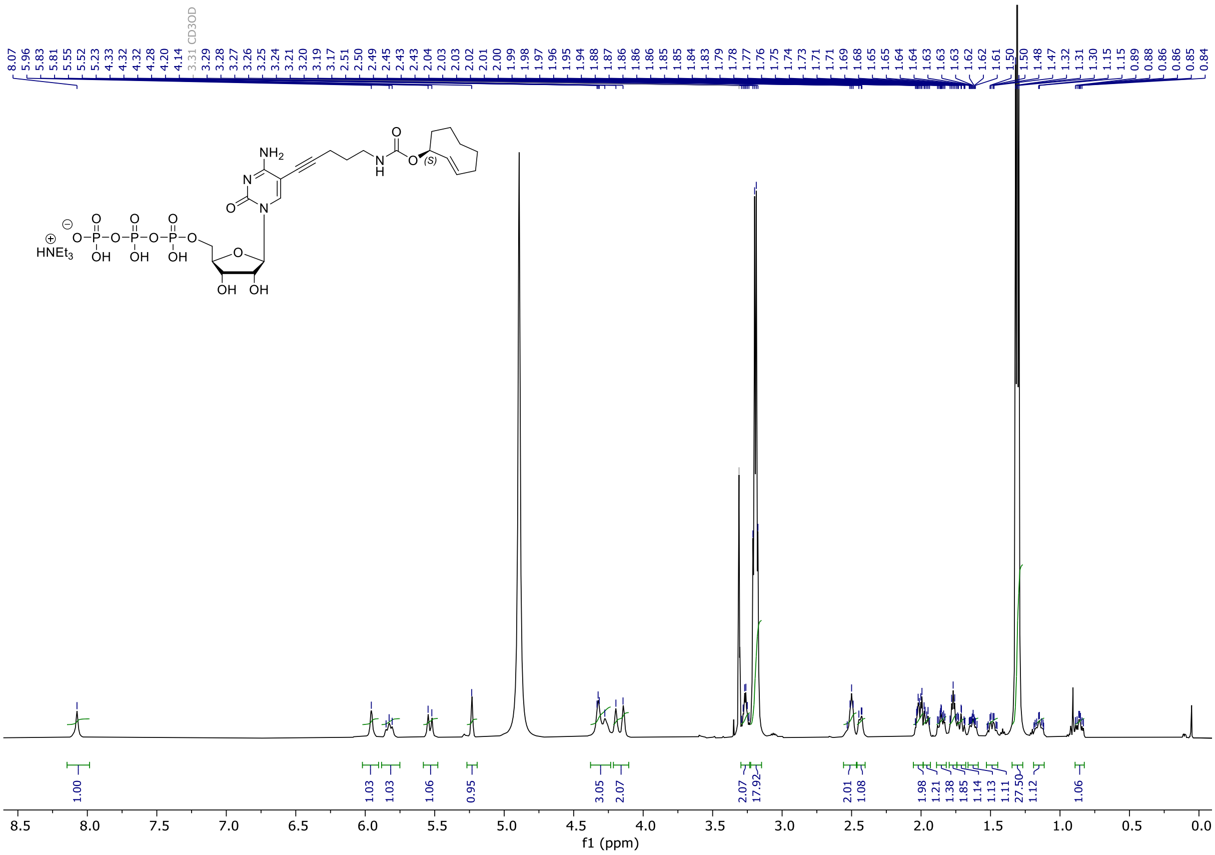

NH2
N
O
NH
(S)
HNEt3
OH
f1 (ppm)

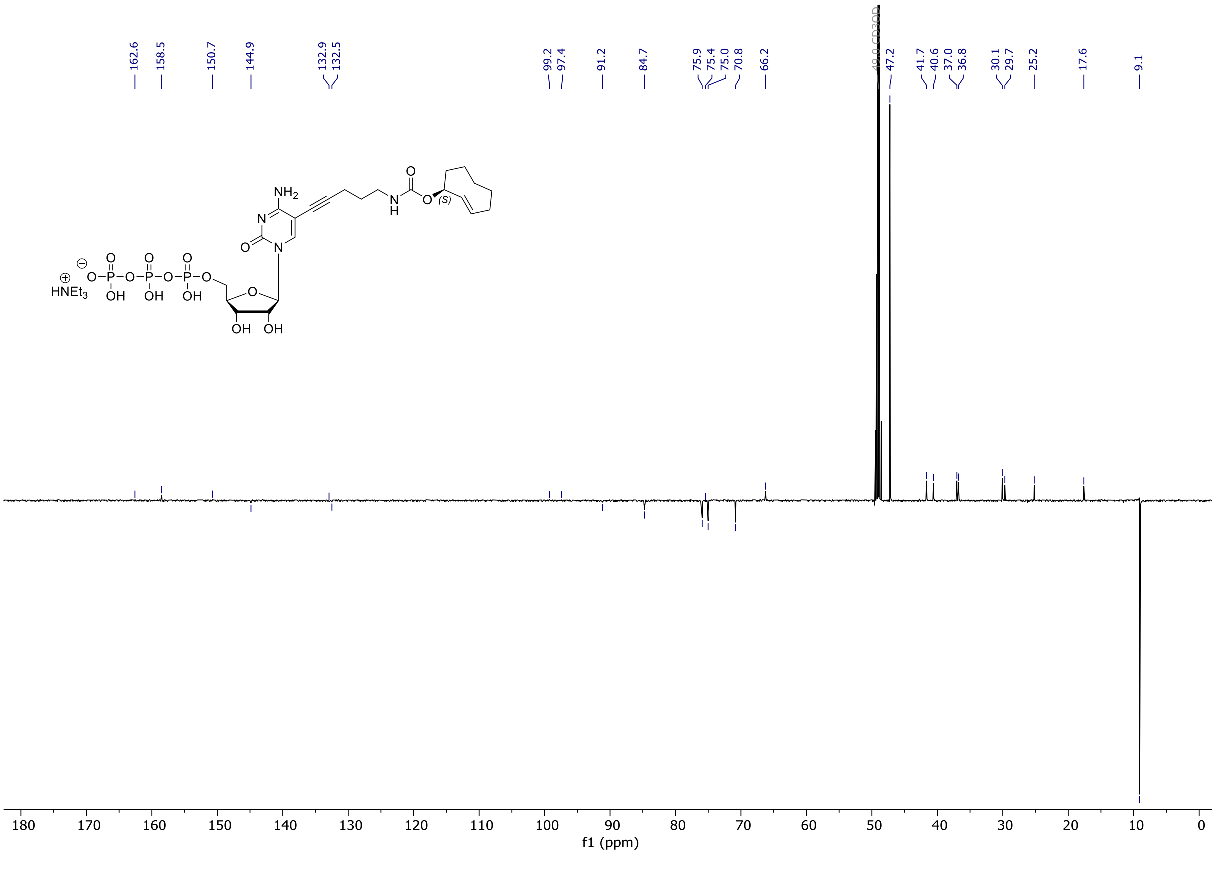

NH2
N
O
NH
(S)
HNEt3
OH
f1 (ppm)

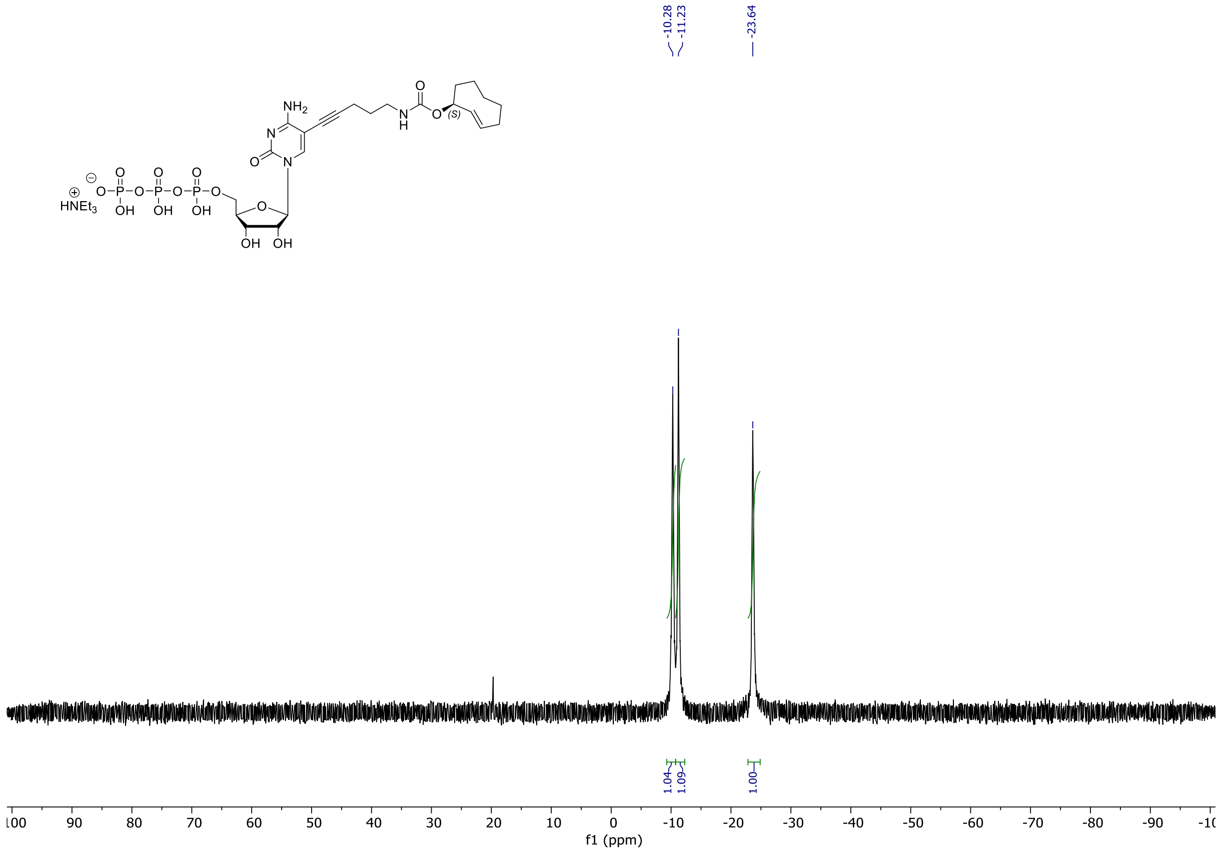

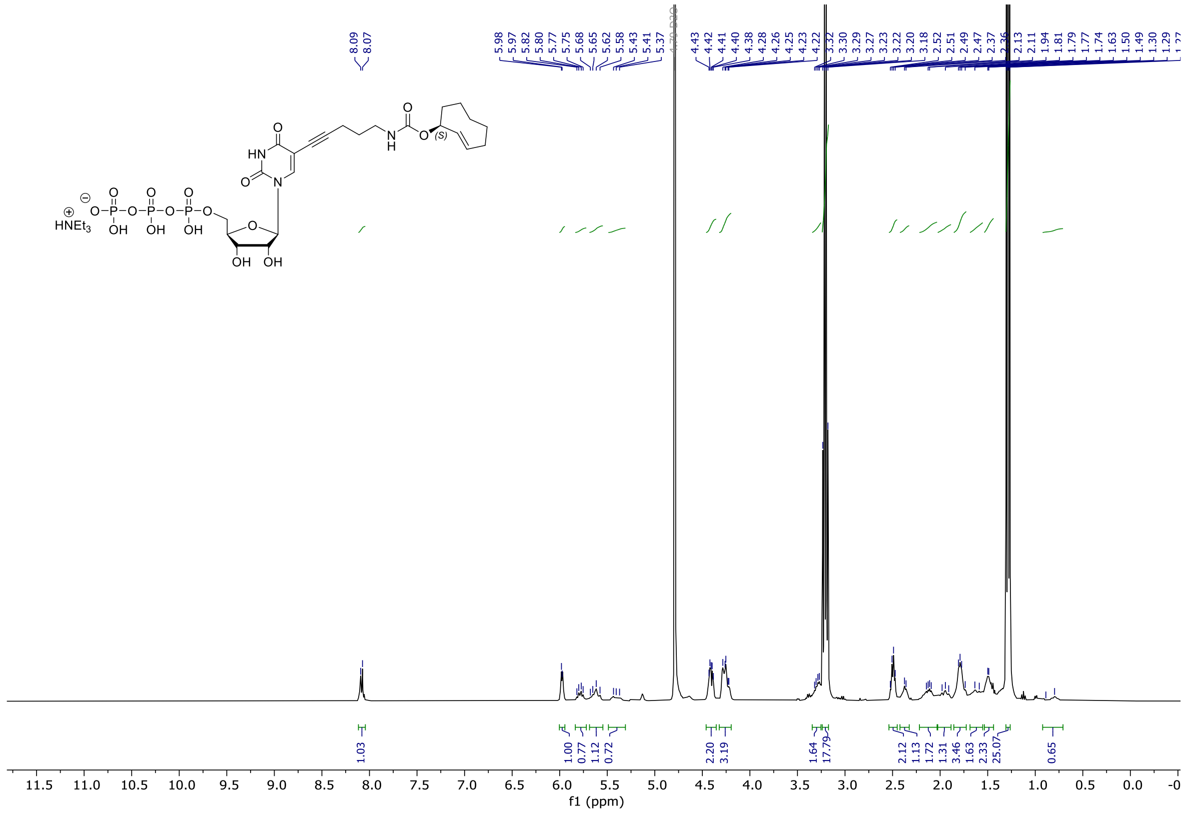

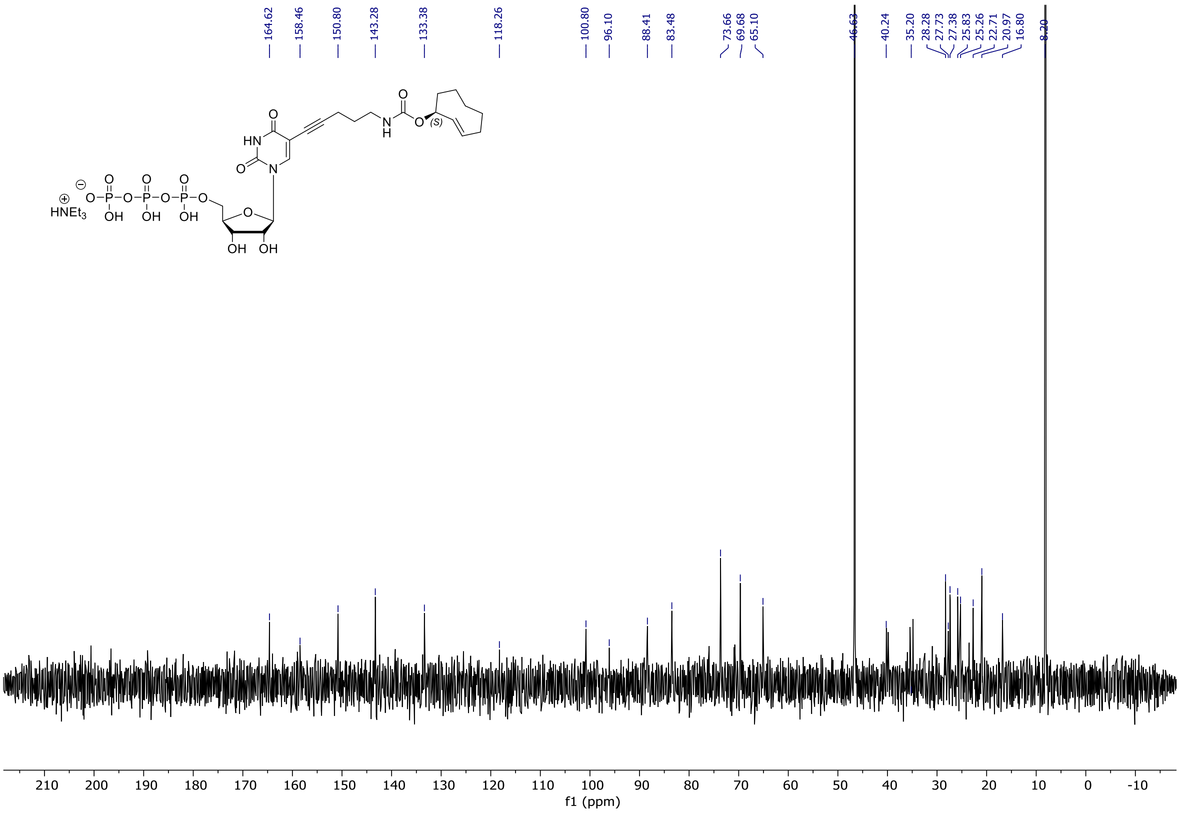
164.62
158.46
150.80
143.28
133.38
118.26
100.80
96.10
88.41
83.48
73.66
69.68
65.10
46.63
40.24
35.20
28.28
27.73
27.38
25.83
25.26
22.71
20.97
16.80
8.20
f1 (ppm)

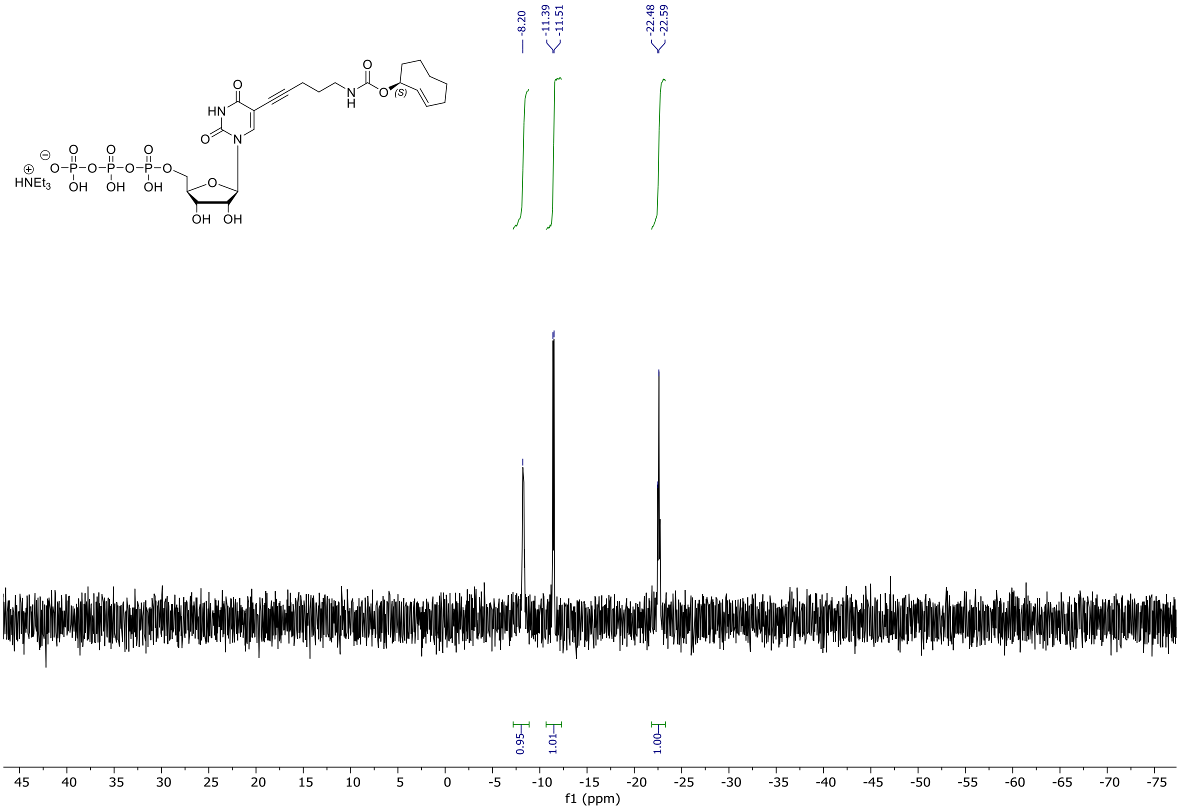
-8.20
-11.39
-11.51
-22.48
-22.59
0.95
1.01
1.00
f1 (ppm)

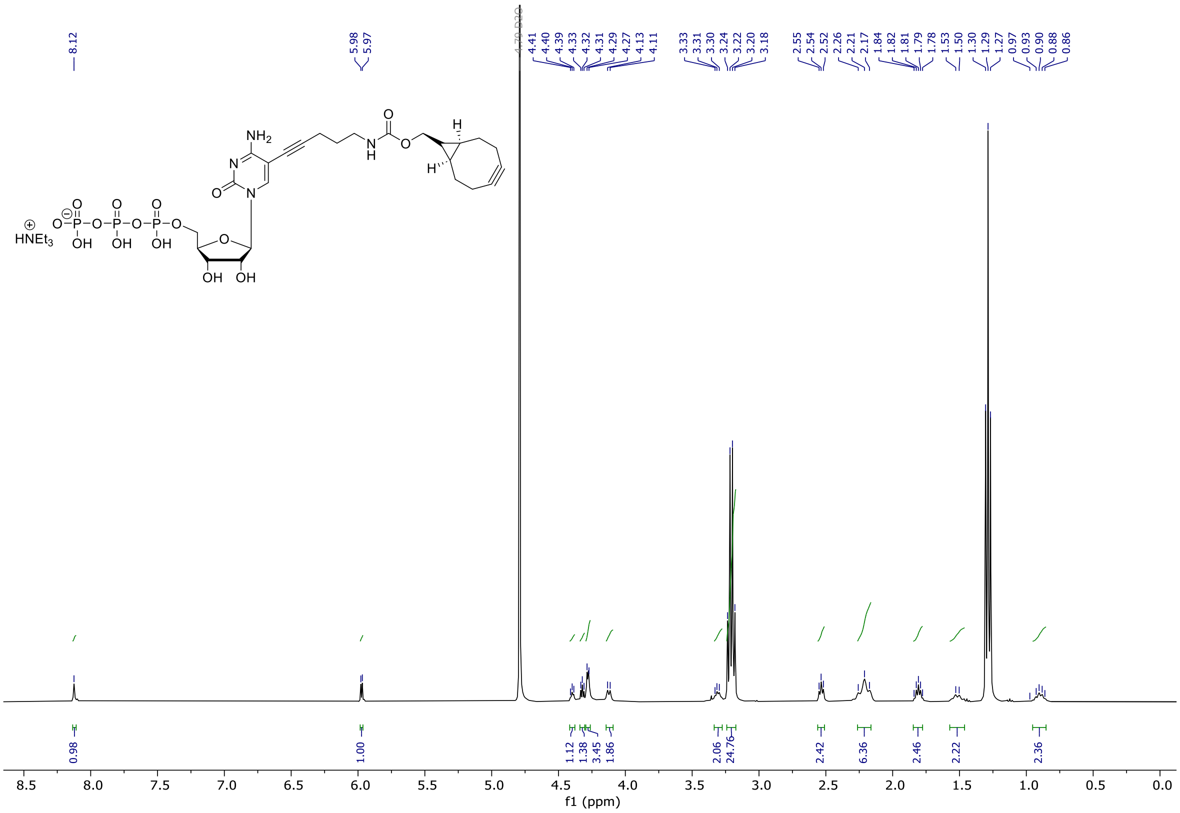

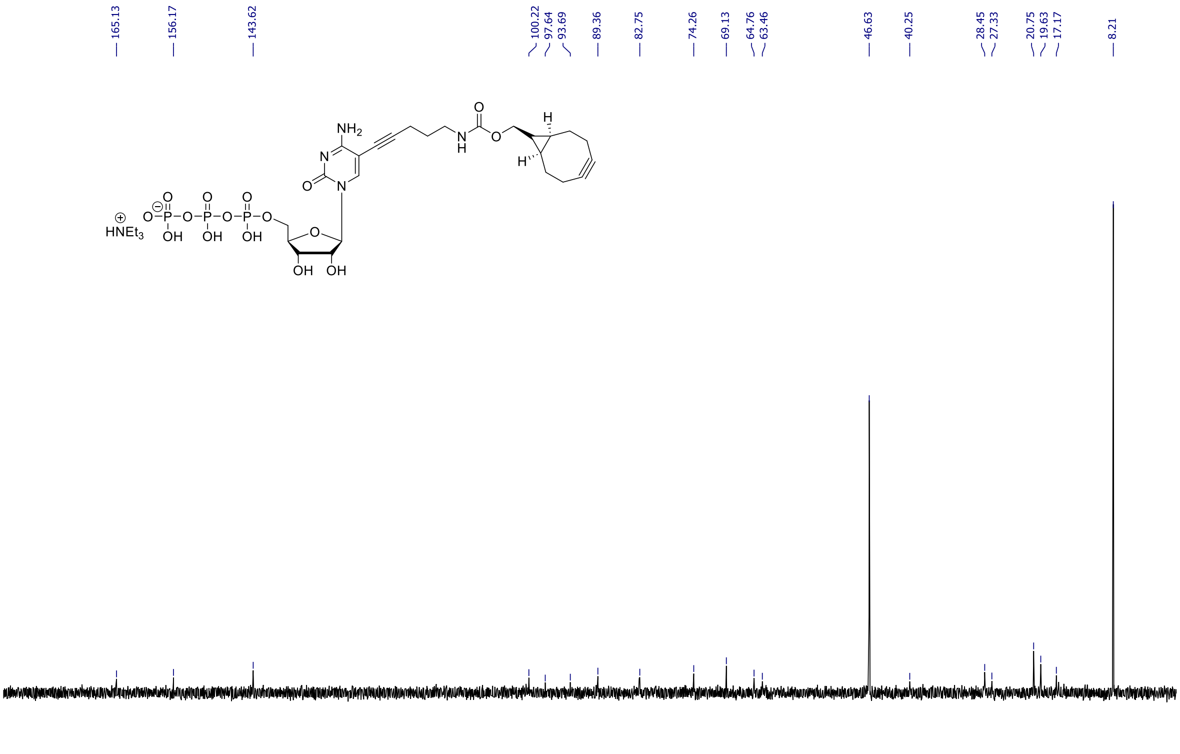

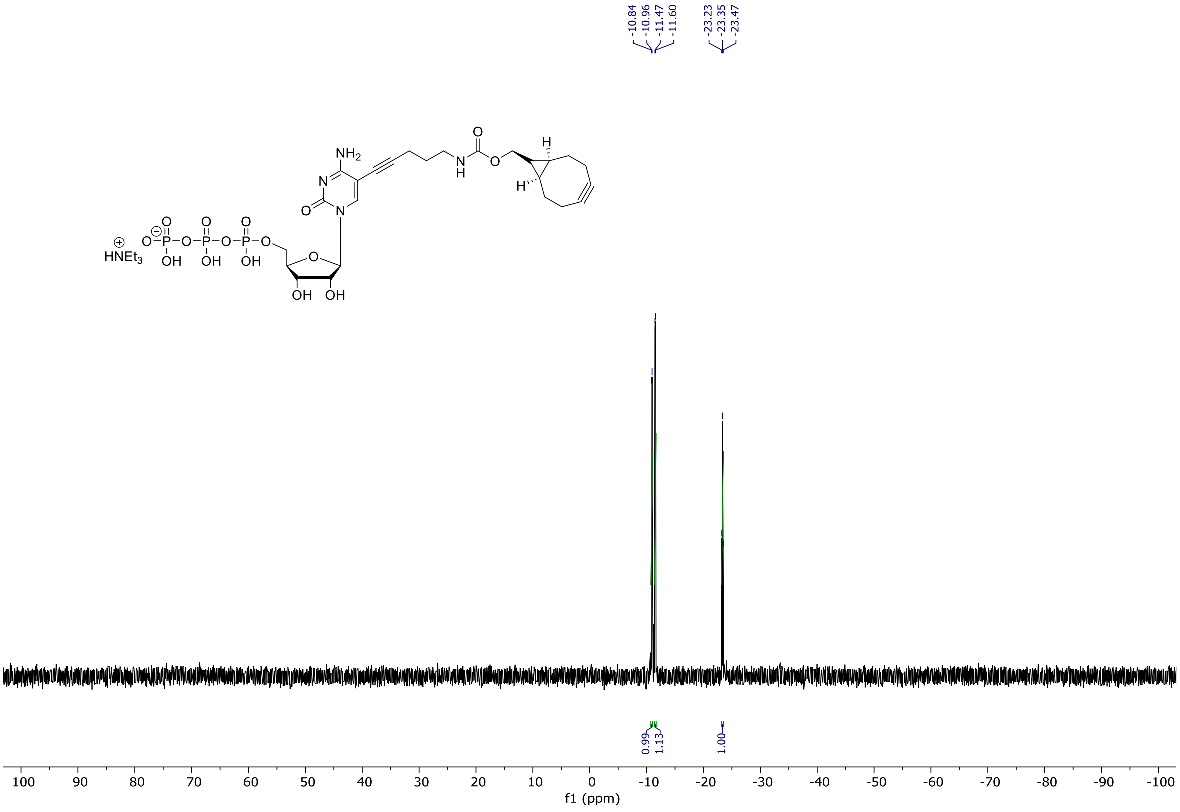

-10.84
-10.96
-11.47
-11.60
-23.23
-23.35
-23.47
NH2
O
N
H
O
HNEt3
OH OH
0.99
1.13
1.00
100 90 80 70 60 50 40 30 20 10 0 -10 -20 -30 -40 -50 -60 -70 -80 -90 -100
f1 (ppm)

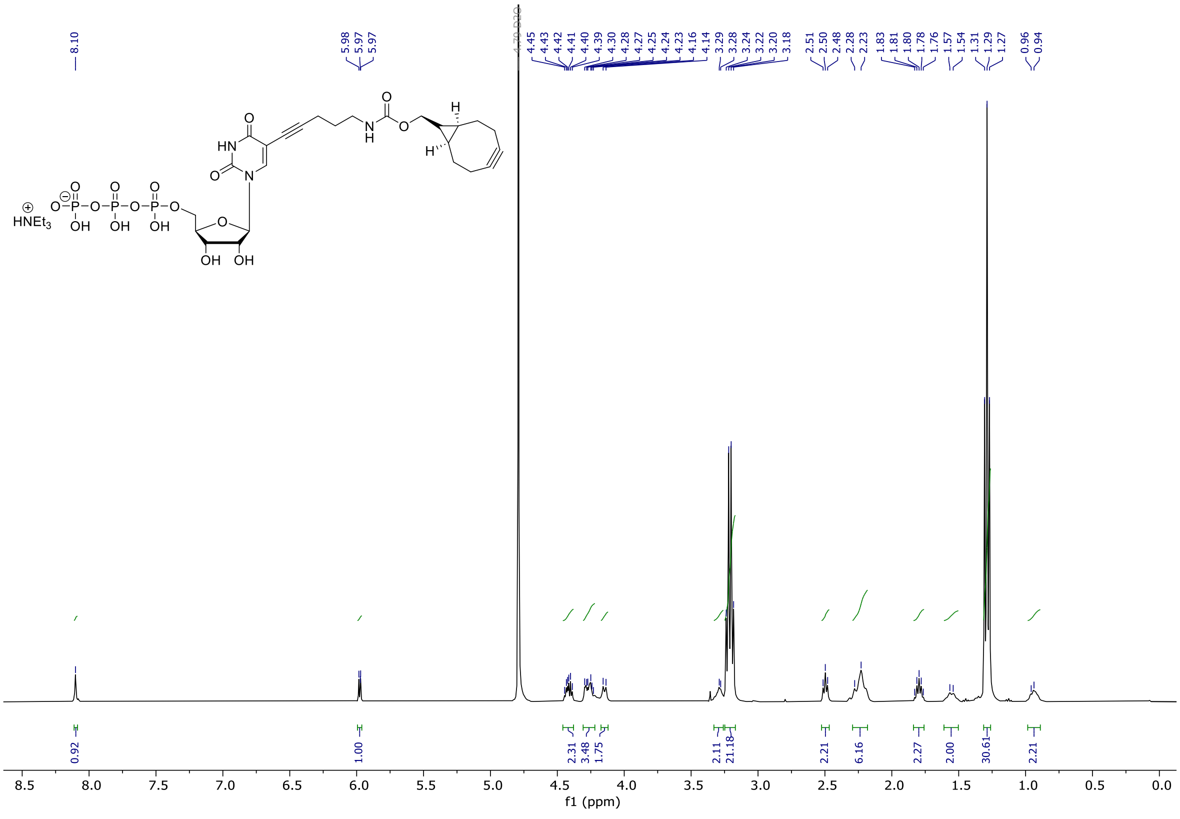

8.10
5.98
5.97
5.97
4.45 4.43 4.42 4.41 4.40 4.39 4.30 4.28 4.27 4.25 4.24 4.23 4.16 4.14
3.29 3.28 3.24 3.22 3.20 3.18
2.51 2.50 2.48 2.28 2.23
1.83 1.81 1.80 1.78 1.76 1.57 1.54 1.31 1.29 1.27
0.96 0.94
HN
O
N
H
HNEt3
OH OH
0.92
1.00
2.31
3.48
1.75
2.11
21.18
2.21
6.16
2.27
2.00
30.61
2.21
8.5 8.0 7.5 7.0 6.5 6.0 5.5 5.0 4.5 4.0 3.5 3.0 2.5 2.0 1.5 1.0 0.5 0.0
f1 (ppm)

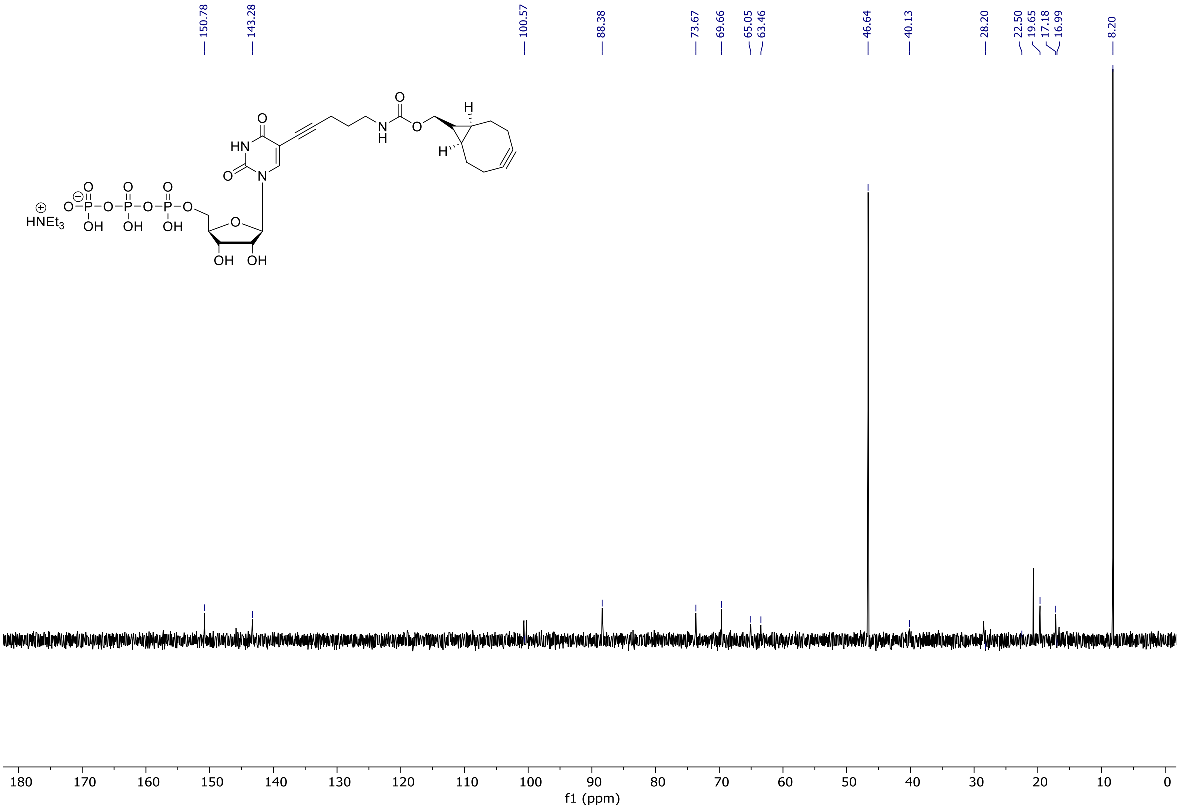
150.78
143.28
100.57
88.38
73.67
69.66
65.05
63.46
46.64
40.13
28.20
22.50
19.65
17.18
16.99
8.20
180 170 160 150 140 130 120 110 100 90 80 70 60 50 40 30 20 10 0
f1 (ppm)

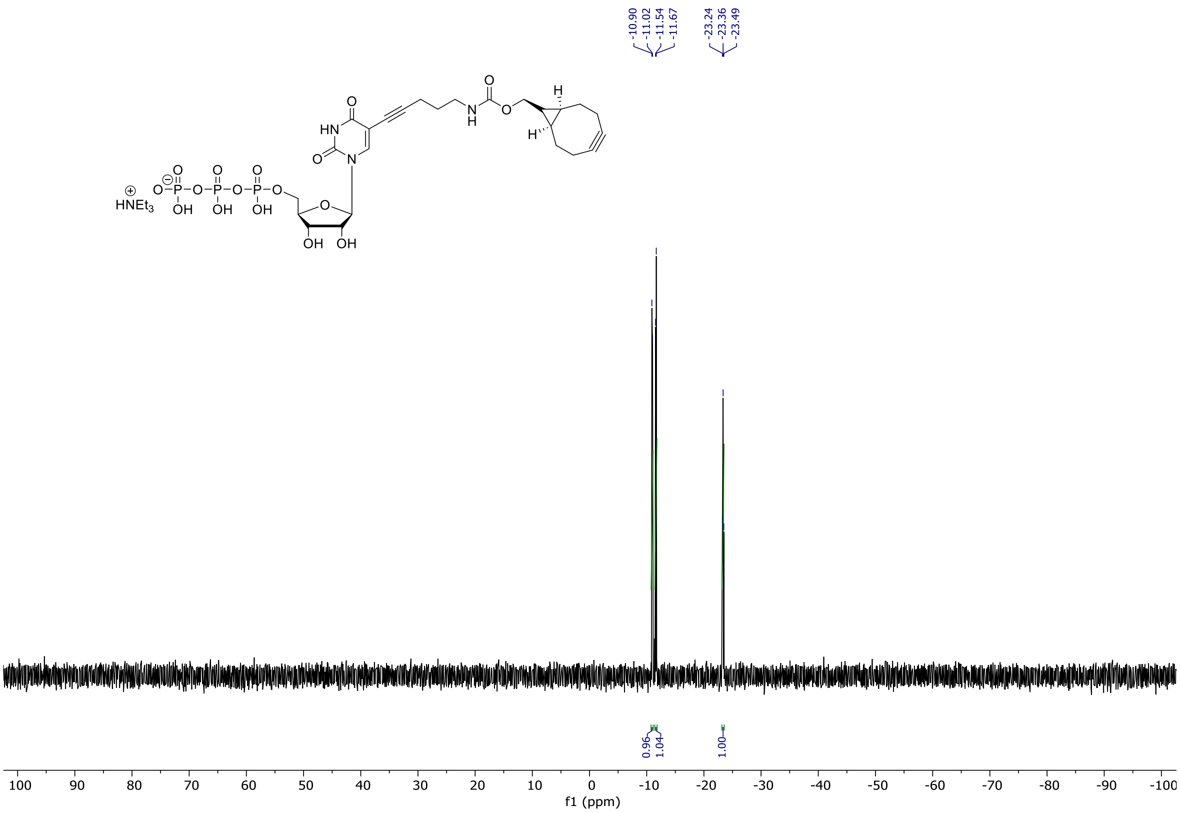
-10.90
-11.02
-11.54
-11.67
-23.24
-23.36
-23.49
0.96
1.04
1.00
100 90 80 70 60 50 40 30 20 10 0 -10 -20 -30 -40 -50 -60 -70 -80 -90 -100
f1 (ppm)

6. References

[1]T. Sowa, S. Ouchi, *Bull. Chem. Soc. Jpn.* **1975**, 48, 2084.

[2]A. R. Kore, M. Shanmugasundaram, A. Senthilvelan, B. Srinivasan, *Nucleosides Nucleotides Nucleic Acids* **2012**, 31, 423.

[3]T.-B. Yu, J. Z. Bai, Z. Guan, *Angew. Chem. Int. Ed.* **2009**, 48, 1097.

[4]V. T. Sterrenberg, D. Stalling, J. I. H. Knaack, T. K. Soh, J. B. Bosse, C. Meier, *Angew. Chem. Int. Ed.* **2023**, 62, e202308271.

[5]S. Mohamady, D. L. Jakeman, *J. Org. Chem.* **2005**, 70, 10588.

[6]X. Hu, T. Zeng, C. C. Husic, M. J. Robb, *ACS Cent. Sci.* **2021**, 7, 1216.

[7]X. Fan, Y. Ge, F. Lin, Y. Yang, G. Zhang, W. S. C. Ngai, Z. Lin, S. Zheng, J. Wang, J. Zhao, J. Li, P. R. Chen, *Angew. Chem. Int. Ed.* **2016**, 55, 14046.
